# Betrixaban activates cGAS-STING to promote antitumor immunity without pathological inflammation

**DOI:** 10.1038/s44321-026-00429-1

**Published:** 2026-05-14

**Authors:** Yang Zhao, Xingyu Chen, LiRui Tang, Hanjie Liu, Boming Kang, Shuailong Zheng, Songying Ouyang, Yunfei Xie, Fuping You

**Affiliations:** 1 Institute of Systems Biomedicine, Department of Immunology, School of Basic Medical Sciences, Beijing Key Laboratory of Tumor Systems Biology, NHC Key Laboratory of Medical Immunology, Peking University Health Science Center, Beijing, China; 2 State Key Laboratory of Membrane Biology, Tsinghua-Peking Center for Life Sciences, Beijing Frontier Research Center for Biological Structure, School of Pharmaceutical Sciences, Tsinghua University, Beijing, China; 3 Tsinghua-Peking Center for Life Sciences, Beijing, China; 4 Key Laboratory of Microbial Pathogenesis and Interventions of Fujian Province University, the Key Laboratory of Innate Immune Biology of Fujian Province, Biomedical Research Center of South China, College of Life Sciences, Fujian Normal University, Fuzhou, China; 5 Department of Endocrinology, Yuquan Hospital, School of Clinical Medicine, Tsinghua University, Beijing, China; 6 Key Laboratory of Swine Genetics and Breeding of Ministry of Agriculture and Rural Affairs, Huazhong Agricultural University, Wuhan, China

**Keywords:** Cancer, Immunology

## Abstract

Effective cancer immunotherapy requires enhancing tumor-targeted immune responses while limiting pathological inflammation, highlighting an urgent need for single agents that can achieve this balance. Here, we reported that Betrixaban (BT), an FDA-approved Factor Xa inhibitor, functioned as a dual immunomodulator that enhanced antitumor immune responses and suppressed hyperinflammation. BT enhanced innate tumor sensing and adaptive immune responses, partly via epigenetic modulation. In mouse tumor models, BT treatment inhibited tumor growth, accompanied by increased infiltration of activated CD8^+^ T cells. Combining BT with immune checkpoint blockade synergistically enhanced antitumor efficacy. Additionally, BT attenuated pathological inflammation by reducing LPS-induced proinflammatory cytokine production and improving survival in a sepsis model. Mechanistically, BT induced a noncanonical, DNA-independent activation of the cGAS-STING pathway, triggering type I interferon signaling without provoking a full inflammatory cascade. These findings highlighted BT as a strategy to promote antitumor immunity while restraining inflammation, potentially improving cancer immunotherapy with reduced inflammatory toxicity.

## Synopsis

This study reports that Betrixaban activates a noncanonical, DNA-independent activation of the cGAS-STING pathway, linking tumor IFN-I induction to inflammation restraint across tumor and sepsis models.

- Betrixaban was shown to suppress Pan02, orthotopic KPC-luc and B16F10 metastatic growth, and PD-1 efficacy was further increased in Pan02 and B16F10 models.
- cGAS dependence was demonstrated in both tumor-intrinsic and host compartments, as BT activity was lost with cGAS^−/−^ Pan02 cells or cGAS^−/−^ mice.
- Single-cell RNA-seq, bulk RNA-seq and ATAC-seq were integrated to show that CD8^+^ TIL effector programs and promoter accessibility were coordinately increased by BT.
- In the LPS challenge model, inflammatory chromatin accessibility and pro-inflammatory gene induction were reduced, and survival was improved.
- Direct biochemical activation of cGAS was detected by SPR and LC-MS, including measurable cGAMP generation without exogenous dsDNA.

## Abstract (web summary)

This study reports that Betrixaban activates a noncanonical, DNA-independent activation of the cGAS-STING pathway, linking tumor IFN-I induction to inflammation restraint across tumor and sepsis models.

## Graphical abstract

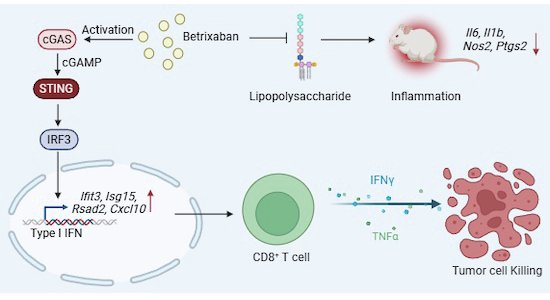

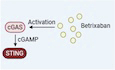

The paper explained **Problem**

A major challenge in cancer immunotherapy is how to enhance tumor-directed immune responses without causing excessive systemic inflammation. Although many immune-activating strategies improve antitumor efficacy, they often do so at the expense of inflammatory toxicity. There is therefore a need for single agents that can strengthen protective antitumor immunity while limiting pathological inflammatory responses.

**Results**

We identified Betrixaban, an FDA-approved oral Factor Xa inhibitor, as a dual immunomodulator with these properties. In multiple mouse tumor models, Betrixaban reduced tumor growth, increased the infiltration and activation of CD8⁺ T cells, and enhanced the therapeutic efficacy of PD-1 blockade. Multi-omic analyses further showed that Betrixaban reshaped the tumor–immune microenvironment toward a more immune-active state. At the same time, Betrixaban suppressed LPS-induced inflammatory gene expression, reduced inflammatory chromatin accessibility, and improved survival in a mouse sepsis model. Mechanistically, Betrixaban directly activated cGAS through a noncanonical, DNA-independent mechanism, thereby inducing type I interferon signaling without triggering a full inflammatory cascade.

**Impact**

These findings revealed a previously unrecognized immunoregulatory function of Betrixaban and suggested a strategy to dissociate beneficial antitumor immunity from harmful hyperinflammation. More broadly, the study supported the idea that noncanonical pharmacological activation of cGAS-STING could be tuned toward interferon-dominant immune protection with limited inflammatory toxicity. This work may provide a basis for repurposing clinically approved drugs and for developing safer immunomodulatory therapies for cancer.

## Introduction

Cancer immunotherapy has transformed outcomes for many cancers, yet durable benefit remains inconsistent across patients and tumor types (Berraondo et al, 2025; Rejeski et al, 2025; Zhang et al, 2025). Effective control typically requires an inflamed, T-cell-infiltrated tumor microenvironment with competent antigen presentation (Beasley et al, 2022; Kortekaas et al, 2021). Type I interferon promotes cross-priming and T-cell trafficking (Mandula et al, 2022; Tu et al, 2022). In contrast, broad inflammation beyond the tumor can trigger immune-related toxicities and constrain dosing. Current strategies, including checkpoint blockade, cytokine therapies, STING agonists, and epigenetic agents, often raise antitumor immunity and systemic inflammation in parallel (Patel and Minn, 2018; Pollack et al, 2018; Xiong et al, 2023). For example, STING agonists can trigger systemic cytokine surges even with intratumoral dosing (Luke et al, 2023). Similarly, epigenetic viral-mimicry agents enhance immunity but carry hematologic and inflammatory toxicities (Patel et al, 2022; Scheller et al, 2021). Thus, strategies that selectively amplify tumor-targeted immunity while restraining pathological inflammation remain a critical unmet need.

Immune checkpoint inhibitors illustrate this challenge, despite inducing durable tumor remissions, they often trigger diverse immune-related toxicities across multiple organs (Reschke et al, 2025; Triantafyllou et al, 2025). Managing such inflammatory side effects, such as colitis, dermatitis, and hepatitis, usually requires immunosuppressive interventions, and severe cases may even force discontinuation of life-saving therapy (Sangro et al, 2020). Clinicians therefore have to balance the need to maximize antitumor immunity against the risk of intolerable inflammatory damage.

Current approaches frequently rely on combining immunotherapies with anti-inflammatory agents, an approach that increases treatment complexity and can compound toxicity. For example, combining anti-CTLA-4 with anti-PD-1 improves tumor control but causes high rates of immune-related adverse events, limiting its use to select patients (Kungwankiattichai et al, 2025; O’Leary et al, 2024). Conversely, mitigating immunotherapy-induced inflammation often requires systemic corticosteroids or cytokine antagonists, such as anti-TNFα, which may dampen antitumor immunity and cause additional adverse effects (Badran et al, 2019; Horvat et al, 2015). In essence, approaches that can dissociate antitumor effects from inflammation are essential to improve the safety profile of immunotherapy (Wang et al, 2023; Wu et al, 2025). An ideal solution would be a single agent capable of simultaneously potentiating antitumor immunity and suppressing pathological inflammation, thereby avoiding the need for multi-drug regimens.

Here, we reported that Betrixaban (BT), an FDA-approved oral Factor Xa inhibitor, unexpectedly fulfilled these criteria. We found that BT enhanced antitumor immunity in mouse tumors while limiting hyperinflammation. BT also showed modest epigenetic effects consistent with increased chromatin accessibility at immune effector genes. BT engaged innate immune sensing pathways and epigenetic modulation to achieve this balance. Through these mechanisms, BT stimulated type I interferon production and adaptive immunity in tumors without provoking a cytokine storm. Guided by these observations, we tested BT with PD-1 blockade and in LPS-driven inflammation, aiming to evaluate whether one small molecule could expand antitumor immunity while restraining systemic inflammatory toxicity. This dual immunomodulatory profile highlighted BT as a potential single-agent strategy to promote antitumor immunity with restrained pathological inflammation, addressing a critical gap in current cancer immunotherapy.

## Results

### Betrixaban promotes antitumor immune responses to enhance PD-1 therapy

Since our previous study revealed that Betrixaban (BT), an FDA-approved Factor Xa inhibitor, directly activated cGAS and derepressed endogenous retroviruses (ERVs) to engage the RIG-I/MDA5 pathway (Kang et al, 2025), we hypothesized that BT might also stimulate innate immune responses against tumor cells.

**Figure 1:**
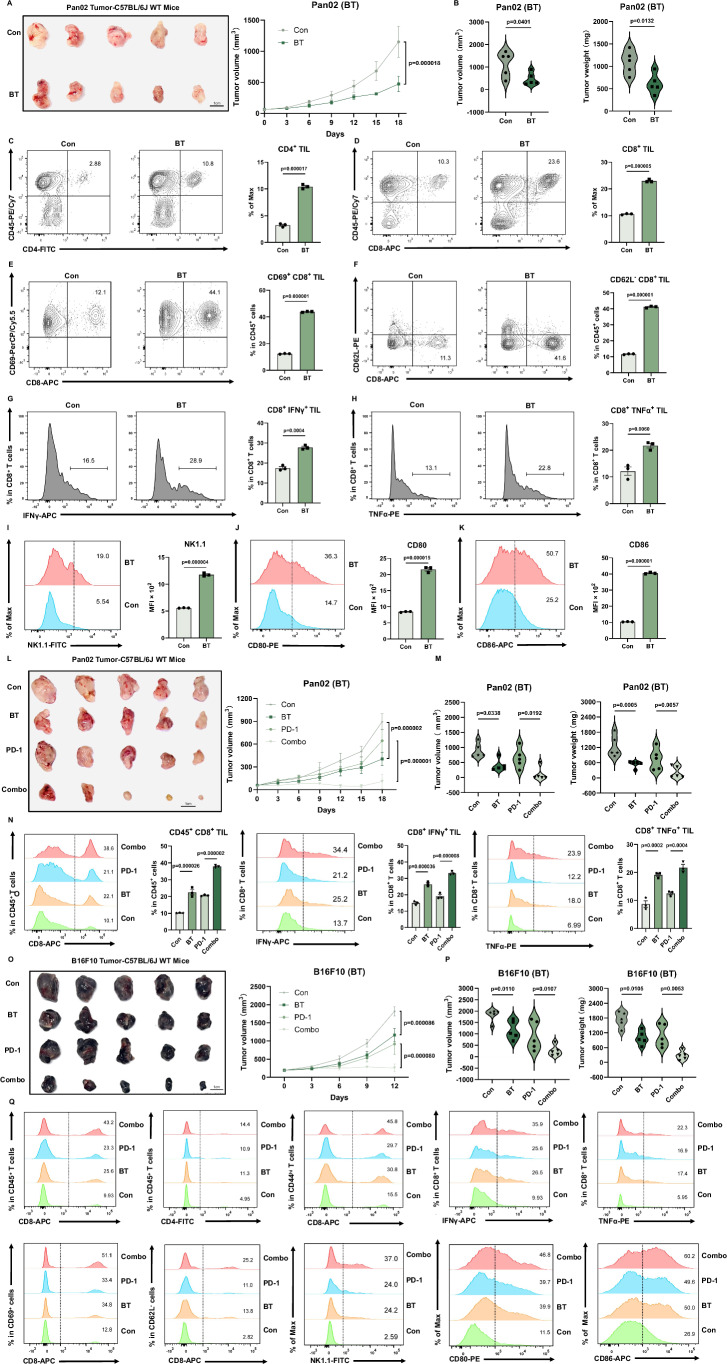
Betrixaban promotes antitumor immune responses to enhance PD-1 therapy. (**A**, **B**) Wild-type (WT) mice were inoculated with the indicated numbers of Pan02 cells subcutaneously. Tumorigenesis was monitored every other day for 18 days. Representative images of tumors, tumor sizes, and tumor weights in PBS-treated control (Con) and BT-treated group. Two-way ANOVA test (**A**) and Unpaired *t* test (**B**) (*n* = 5). (**C**–**K**) Representative FACS data and quantification of tumor-infiltrating CD45^+^ CD4^+^ TILs (**C**), CD45^+^ CD8^+^ TILs (**D**), CD69^+^ CD8^+^ TILs (**E**), CD62L^-^ CD8^+^ TILs (**F**), CD8^+^ IFNγ^+^ TILs (**G**), CD8^+^ TNFα^+^ TILs (**H**), NK1.1^+^ TILs (**I**), CD80^+^ TILs (**J**), and CD86^+^ TILs (**K**) of mice as in (**A**). Unpaired *t* test (*n* = 3). (**L**, **M**) Tumor sizes of subcutaneous Pan02 implanted in mice treated with the isotype antibody (200 μg/mouse i.p.), BT (50 mg/kg i.p.), anti-PD-1 antibody (200 μg/mouse i.p.), or BT plus anti-PD-1 antibody (Combo, combined treatment with BT and anti-PD-1 antibody). Tumorigenesis was monitored every other day for 18 days. Representative images of tumors, tumor sizes, and tumor weights. Two-way ANOVA test (**L**) and Ordinary one-way ANOVA test (**M**) (*n* = 5). (**N**) Representative FACS data and quantification of tumor-infiltrating CD45^+^ CD8^+^ TILs, CD8^+^ IFNγ^+^ TILs, and CD8^+^ IFNγ^+^ TILs of mice as in (**L**). Unpaired *t* test (*n* = 3). (**O**, **P**) Tumor sizes of subcutaneous B16F10 implanted in mice treated with the isotype antibody (200 μg/mouse i.p.), BT (50 mg/kg i.p.), anti-PD-1 antibody (200 μg/mouse i.p.), or BT plus anti-PD-1 antibody (Combo, combined treatment with BT and anti-PD-1 antibody). Tumorigenesis was monitored every other day for 12 days. Representative images of tumors, tumor sizes, and tumor weights. Two-way ANOVA test (**O**) and Ordinary one-way ANOVA test (**P**) (*n* = 5). (**Q**) Representative FACS data and quantification of tumor-infiltrating CD45^+^ CD4^+^ TILs, CD45^+^ CD8^+^ TILs, CD44^hi^ CD8^+^ TILs, CD69^+^ CD8^+^ TILs, CD62L^-^ CD8^+^ TILs, CD8^+^ IFNγ^+^ TILs, CD8^+^ TNFα^+^ TILs, CD80^+^ TILs, CD86^+^ TILs, and NK1.1^+^ TILs of mice as in (**O**). Data are shown as mean ± SEM. Source data are available online for this figure.

**Figure EV1:**
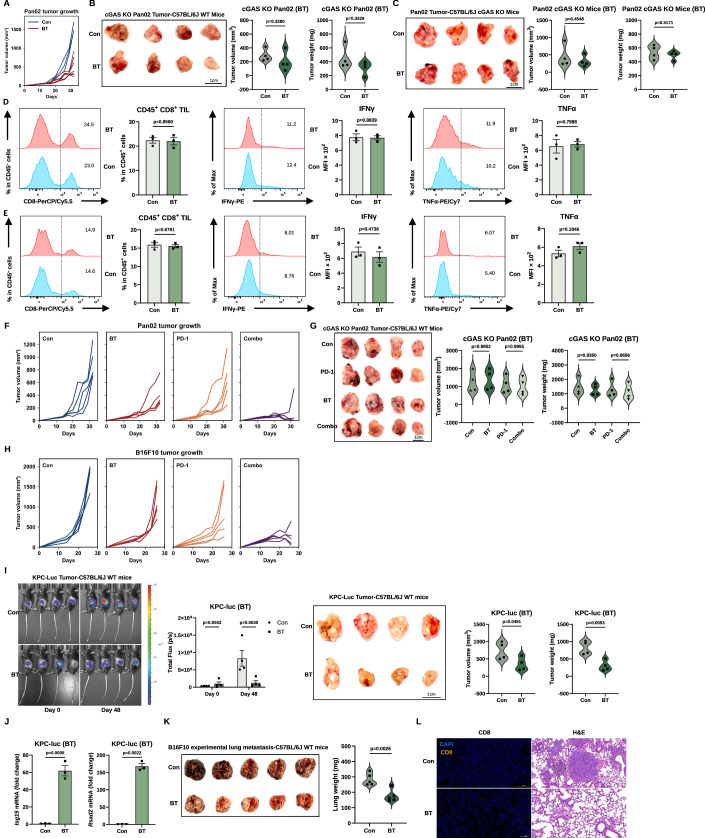
The antitumor ability of Betrixaban. (**A**) Individual tumor growth curves for each mouse, corresponds to Fig. 1A. (**B**) Wild-type (WT) mice were inoculated with the indicated numbers of cGAS^−/−^ Pan02 cells subcutaneously. Tumorigenesis was monitored every other day for 18 days. Representative images of tumors, tumor sizes, and tumor weights in PBS-treated control (Con) and BT-treated group. Unpaired *t* test (*n* = 4). (**C**) cGAS knockout mice were inoculated with the indicated numbers of Pan02 cells subcutaneously. Then treated with PBS or BT every two days. Representative images of tumors, tumor sizes, and tumor weights in PBS-treated control (Con) and BT-treated group. Unpaired *t* test (*n* = 4). (**D**) Representative FACS data and quantification of tumor-infiltrating CD45^+^ CD8^+^ TILs, IFNγ^+^ TILs, TNFα^+^ TILs of mice as in (**B**). Unpaired *t* test (*n* = 3). (**E**) Representative FACS data and quantification of tumor-infiltrating CD45^+^ CD8^+^ TILs, IFNγ^+^ TILs, TNFα^+^ TILs of mice as in (**C**). Unpaired *t* test (*n* = 3). (**F**) Individual tumor growth curves for each mouse, corresponds to Fig. 1L. (**G**) Tumor sizes of subcutaneous Pan02 implanted in cGAS knockout mice treated with the isotype antibody (200 μg/mouse i.p.), BT (50 mg/kg i.p.), anti-PD-1 antibody (200 μg/mouse i.p.), or BT plus anti-PD-1 antibody (Combo, combined treatment with BT and anti-PD-1 antibody). Tumorigenesis was monitored every other day for 18 days. Representative images of tumors, tumor sizes, and tumor weights. Ordinary one-way ANOVA test (*n* = 4). (**H**) Individual tumor growth curves for each mouse, corresponds to Fig. 1O. (**I**) KPC-luc cells were implanted orthotopically into the pancreas of C57BL/6 mice to establish an orthotopic KPC-luc pancreatic tumor model. Tumor burden was longitudinally monitored using bioluminescent imaging. Representative bioluminescent images as well as tumor photographs, tumor volume, and tumor weight were shown. (**J**) BT upregulates *Isg15* and *Rsad2* mRNA in KPC-Luc cells. (**K**) Representative images of the B16F10 experimental lung metastasis model and lung weights. (**L**) Representative CD8 immunofluorescence images and H&E staining images from (**K**). Data are shown as mean ± SEM.

To test this hypothesis, we subcutaneously inoculated Pan02 murine pancreatic cancer cells into wild-type C57BL/6J mice and treated them with BT every other day. Representative images showed markedly reduced tumor sizes following BT treatment compared to controls (Fig. 1A and Fig. EV1A). Tumor growth curves confirmed significant inhibition of tumor progression, as indicated by reduced tumor volume and weight in BT-treated mice (Fig. 1B).

Given the critical role of tumor-infiltrating lymphocytes (TILs) in determining antitumor immunity, we next assessed the immune cell populations within Pan02 tumors. Flow cytometry analysis showed that BT significantly increased infiltration of CD4⁺ TILs and CD8⁺ TILs compared to untreated controls (Fig. 1C,D). Among these CD8⁺ TILs, BT markedly increased the proportion of cells expressing CD69 (Fig. 1E), an early activation marker indicating recent T-cell receptor stimulation (Bendelac et al, 1992). BT treatment also increased the percentage of CD62L⁻CD8⁺ TILs (Fig. 1F), a phenotype representing effector T cells that have lost lymph node-homing capacity and are associated with active antitumor responses (Biktasova et al, 2015). Functionally, BT enhanced the production of the cytotoxic cytokines IFN-γ and TNF-α by CD8⁺ TILs (Fig. 1G,H), further confirming increased antitumor immune activity.

We also evaluated innate immune cell activation within the tumor microenvironment. BT treatment significantly elevated the infiltration of NK1.1⁺ NK cells, which were innate lymphoid cells critical for initial tumor control (Fig. 1I). In addition, the expression levels of CD80 and CD86, two critical co-stimulatory molecules involved in antigen presentation and T-cell activation, were significantly increased (Fig. 1J,K). Collectively, these results indicated that BT not only promoted adaptive T cell responses but also boosted innate immunity and antigen presentation, thus shaping a more activated tumor–immune microenvironment.

To further validate the cGAS-dependent mechanism of BT, we used four experimental groups, including both *cGAS*^−/−^ tumor cell and *cGAS*^−/−^ host models. First, we implanted *cGAS*^−/−^ Pan02 cells in WT mice and treated them with BT or PBS. Tumor images showed no significant reduction in tumor size compared to PBS-treated controls (Fig. EV1B). Similarly, in *cGAS*^−/−^ mice bearing WT Pan02 tumors, BT also failed to reduce tumor growth. Consistently, tumor volume and tumor weight confirmed that BT treatment had no significant effect (Fig. EV1C). Flow cytometry analysis of tumors showed BT failed to increase infiltration of CD8⁺ TILs or their IFN-γ/TNF-α production (Fig. EV1D,E). These results confirmed that both tumor and host cGAS were essential for the full antitumor effect of BT.

To further explore the therapeutic potential of BT in combination with immune checkpoint blockade therapy, we treated mice bearing Pan02 tumors with BT, PD-1 antibody, or their combination. Combination therapy significantly inhibited tumor growth compared to either single treatment or control (Fig. 1L,M and Fig. EV1F). Flow cytometry analysis of tumors showed a marked increase in total CD8⁺ TILs, as well as IFN-γ⁺CD8⁺ and TNF-α⁺CD8⁺ populations (Fig. 1N), demonstrating enhanced cytotoxic function and effector differentiation induced by combined treatment. Consistently, BT failed to inhibit tumor growth in *cGAS*^−/−^ mice, and the BT plus anti-PD-1 combination did not provide additional benefit beyond anti-PD-1 alone (Fig. EV1G).

We validated these findings using the B16F10 melanoma model, known for its low immunogenicity. Similarly, the combination of BT and PD-1 antibody markedly delayed tumor growth (Fig. 1O,P and Fig. EV1H). Flow cytometry analysis showed increased infiltration of both CD8⁺ and CD4⁺ TILs after treatment. The combination therapy (Combo) significantly enhanced the proportion of activated (CD69⁺) and effector (CD62L^−^) CD8⁺ TILs compared to the PD-1 and BT groups. Similarly, CD8⁺ TILs expressing CD44, a marker for antigen-experienced T cells, were higher in the combination group, indicating improved antitumor T-cell responses. Cytokine production by CD8⁺ TILs, including IFN-γ and TNF-α, was elevated in the combination group, confirming enhanced cytotoxic activity. NK1.1⁺ NK cells were also elevated in the tumor microenvironment, particularly in the combination group, along with increased CD80 and CD86 expression, which are key co-stimulatory molecules. These results confirm improved innate immune activation and antigen-presenting functions in the tumor. (Fig. 1Q).

To evaluate BT in a more physiological pancreatic tumor setting, we established an orthotopic KPC-luc model. BT treatment markedly reduced orthotopic tumor burden, as shown by lower bioluminescent signals and decreased pancreatic tumor weight compared with vehicle (Fig. EV1I). Consistent with activation of an interferon response, qPCR analysis of orthotopic tumors showed significant upregulation of the interferon-stimulated genes *Isg15* and *Rsad2* in BT-treated mice (Fig. EV1J). To assess the effect of BT on metastatic disease, we further used a B16F10 experimental lung metastasis model via tail-vein injection. Compared with vehicle-treated mice, BT-treated animals developed markedly fewer pulmonary metastatic nodules and had reduced lung weight at endpoint (Fig. EV1K). To further validate the observed phenotype at the histological level, we performed immunofluorescence staining for CD8 and hematoxylin and eosin (H&E) staining on lung tissue sections. CD8 immunofluorescence revealed increased infiltration of CD8⁺ T cells in the lungs of BT-treated mice, suggesting that the reduction in metastatic burden was accompanied by enhanced antitumor immune responses. H&E staining showed that control lungs were extensively infiltrated by tumor cells, with thickened alveolar septa and disrupted lung architecture. In contrast, BT-treated lungs exhibited smaller metastatic foci and markedly preserved lung structure (Fig. EV1L). Taken together, these results demonstrated that BT exerted a clear anti-metastatic effect in the B16F10 lung metastasis model, which was accompanied by enhanced CD8⁺ T cell-associated immune features.

Together, these results demonstrated that BT effectively potentiated PD-1 blockade by enhancing both innate immune activation and adaptive T cell function, and exerted robust antitumor activity across subcutaneous, orthotopic, and metastatic tumor models.

### Betrixaban enhances CD8⁺ TIL effector programs via transcriptional and epigenetic remodeling

**Figure EV2:**
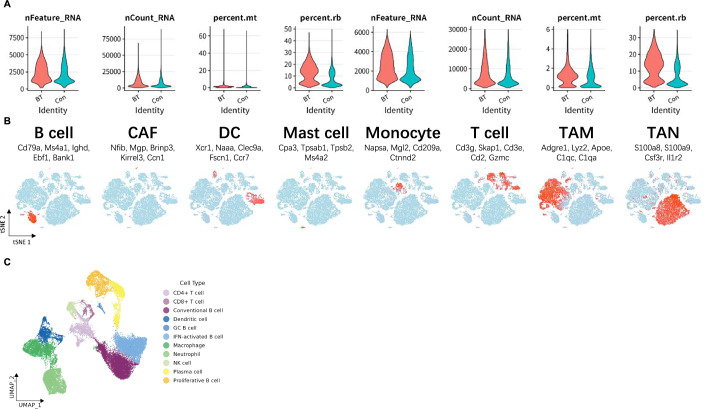
Betrixaban enhances CD8⁺ TIL effector programs via transcriptional and epigenetic remodeling. (**A**) The panel showed pre-QC distributions of key quality metrics and the corresponding post-QC distributions following application of our filtering criteria. (**B**) Key marker gene expression across cell clusters. (**C**) UMAP plot illustrating cell population clustering and annotation in CD45^+^ Pan02 tumor samples treated with PBS (Con) and BT, with clusters color-coded by cell type.

**Figure 2:**
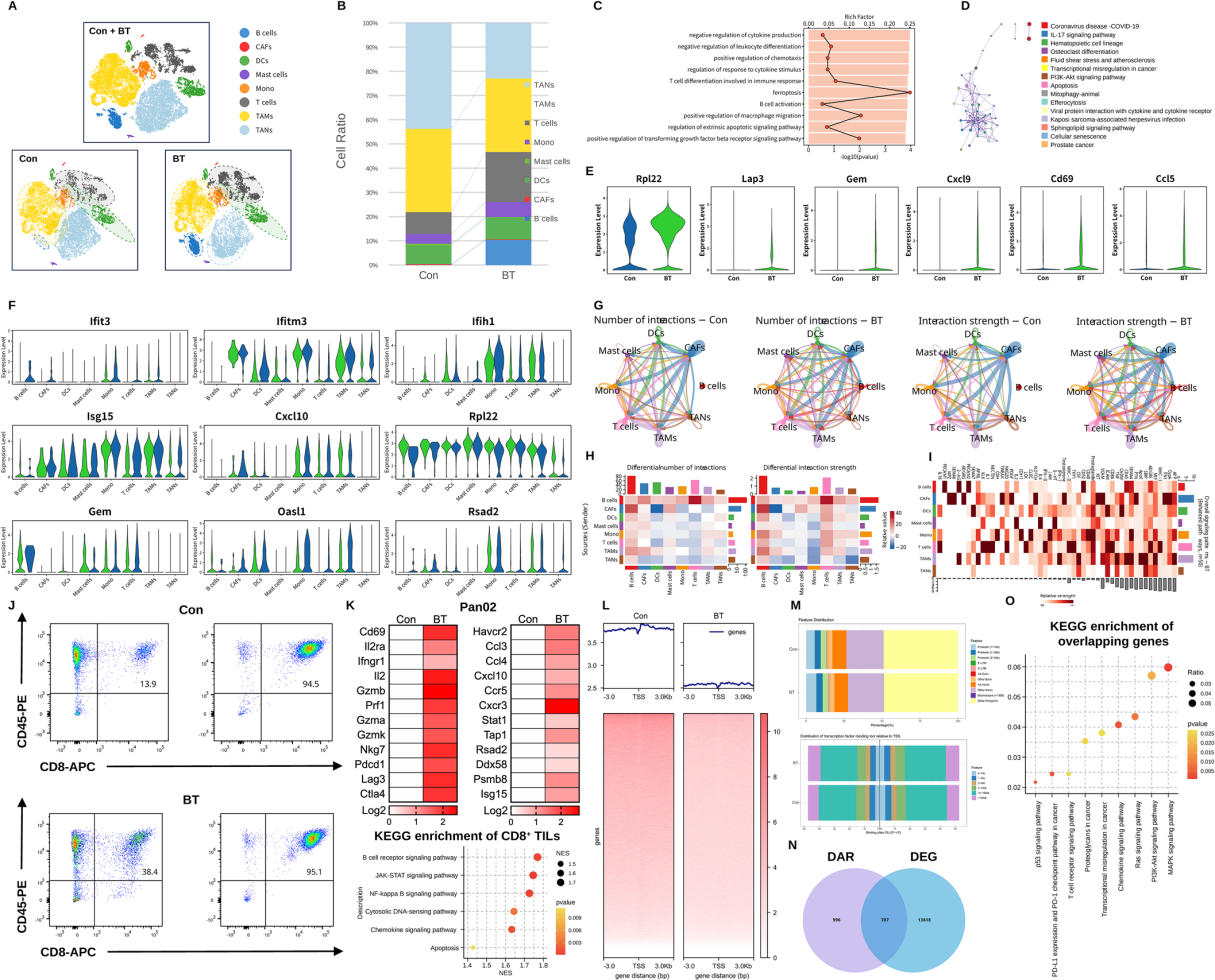
Betrixaban enhances CD8⁺ TIL effector programs via transcriptional and epigenetic remodeling. (**A**) t-SNE plot illustrating cell population clustering and annotation in CD45^+^ Pan02 tumor samples treated with PBS (Con) or BT, with clusters color-coded by cell type. (**B**) Bar chart comparing the proportions of major cell types between BT- and PBS-treated groups in CD45^+^ Pan02 tumors. (**C**) Gene Ontology (GO) enrichment analysis of differentially expressed genes (DEGs) in CD45^+^ cells following BT treatment compared to Con. Fisher’s exact test. (**D**) KEGG pathway analysis of differentially expressed genes (DEGs) in CD45^+^ cells following BT treatment compared to Con. (**E**) Violin plots comparing the expression of critical genes in BT- and PBS-treated CD45^+^ Pan02 tumor cells (*n* = 1). (**F**) Violin plots showing the expression of *Ifit3*, *Ifitm3*, *Ifih1*, *Isg15*, *Cxcl10*, *Rpl22*, *Gem*, *Oasl1*, and *Rsad2* in different cell types, which were significantly upregulated in BT-treated groups compared to Con (*n* = 1). (**G**) Interaction network between different cell types in the tumor microenvironment, showing predicted interactions. Line thickness indicated the number of interactions or the strength of interactions. (**H**) Heatmap of the differential number of interactions across various cell types in the BT and Con groups. (**I**) Heatmap of gene expression across various cell types in the BT- and PBS-treated groups. (**J**) Representative FACS data of tumor-infiltrating CD45^+^ CD8^+^ TILs in the BT and Con, before and after sorting CD8^+^ T cells. (**K**) Heatmap of DEGs in CD8^+^ TILs of Pan02 tumors and KEGG pathway analysis of DEGs. (**L**) ATAC-seq metaprofile (top) and heatmap (bottom) of chromatin accessibility ± 3 kb around peak centers in DMSO- and BT-treated groups. (**M**) Distance-to-TSS annotation and genomic feature distribution of ATAC peaks in BT and DMSO samples. (**N**) Venn diagram showed overlap of upregulated genes between ATAC-seq and bulk RNA-seq. (**O**) GO and KEGG enrichment analysis of overlap genes in (**N**). Source data are available online for this figure.

To comprehensively understand how Betrixaban (BT) potentiated antitumor immunity, we performed single-cell RNA sequencing (scRNA-seq) on CD45⁺ tumor-infiltrating leukocytes (TILs) from BT-treated and PBS-treated (Con) tumors. Initially, we conducted sub-clustering and annotation of these cell populations, which enabled us to assess the impact of BT on gene expression and pathway alterations within each subpopulation (Fig. EV2A). The gene markers used for cell annotation are shown in Fig. EV2B. Unsupervised clustering using t-distributed stochastic neighbor embedding (t-SNE) of TILs analysis identified eight major immune cell populations, including T cells, B cells, dendritic cells (DCs), mast cells, monocytes (Mono), tumor‑associated macrophages (TAMs), tumor‑associated neutrophils (TANs), and cancer‑associated fibroblasts (CAFs) (Fig. 2A). Consistent results were obtained when visualizing the same data with UMAP, which showed the same separation of major immune cell lineages (Fig. EV2C).

In BT-treated tumors, we observed significant compositional changes in immune cell populations compared to controls (Fig. 2B). Notably, the proportion of T cells, DCs, and B cells was significantly increased compared to controls, whereas TANs were relatively declined. These data indicated that BT therapy remodeled the tumor microenvironment to favor antitumor effector. Gene Ontology and KEGG pathway enrichment analysis of differentially expressed genes (DEGs) revealed an upregulation of immune activation-related pathways (Fig. 2C,D). Moreover, the BT-treated group markedly increased expression of *Cd69*, *Cxcl9*, and *Ccl5*, indicating stronger T cell activation and recruitment signals, while *Rpl22*, *Lap3*, and *Gem* also presented obvious upregulations (Fig. 2E). In addition, BT treatment selectively upregulated key interferon-stimulated genes (ISGs) across various cell subsets. These ISGs included *Ifit3*, *Ifitm3*, *Ifih1*, *Isg15*, *Cxcl10*, *Rpl22*, *Gem*, *Oasl1*, and *Rsad2*, as could be observed from the expression level distributions across different cell types (B cells, CAFs, DCs, etc.) in the corresponding plots (Fig. 2F).

CellChat analysis revealed enhanced intercellular communication within the tumor–immune microenvironment following BT treatment. The interaction network diagrams clearly illustrated increased numbers and strengths of cellular interactions compared to controls, predominantly driven by T cells, dendritic cells (DCs), and monocytes (Fig. 2G,H). The differential heatmap confirmed these increases, highlighting elevated outgoing signals from these immune populations. Signaling pathway analysis indicated notable upregulation of pathways including APP (amyloid precursor protein), CypA (Cyclophilin A), and FN1 (Fibronectin 1) in the BT-treated group (Fig. 2I). Collectively, these results demonstrated that BT treatment promoted robust immune cell interactions and enhanced signaling pathways relevant to antitumor immunity.

To further validate these findings at the cell population level, we sorted CD8⁺ TILs from Pan02 tumors and performed bulk RNA‑seq to confirm their transcriptional activation (Fig. 2J). BT-treated CD8⁺ T cells exhibited significantly higher expression of genes associated with T cell activation and effector function, including *Cd69*, *Il2ra*, *Ifngr1*, *Il2*, *Gzma*, and *Prf1*, compared to controls. Meanwhile, interferon‑stimulated genes *Stat1* and *Isg15* were elevated, consistent with a robust antiviral and antitumor response. Furthermore, *Pdcd1*, *Lag3*, and *Ctla4* also increased, showing engagement of inhibitory checkpoints to balance the response. KEGG enrichment analysis of CD8^+^ TILs sorted from Pan02 tumors highlighted JAK-STAT signaling pathway, NF-κB signaling pathway, and chemokine signaling pathway (Fig. 2K).

We next examined whether the transcriptional enhancements in BT-treated T cells were accompanied by epigenetic remodeling (Fig. 2L). Assay for transposase-accessible chromatin using sequencing (ATAC-seq) on CD8^+^ TILs demonstrated that BT treatment broadly increased chromatin accessibility across the genome, with a marked enrichment of open chromatin at gene promoter regions (Fig. 2M). Integrative analysis intersecting the ATAC-seq data with the bulk RNA-seq results revealed that 787 upregulated genes in BT-treated CD8^+^ T cells also displayed increased promoter accessibility (Fig. 2N). KEGG enrichment of these overlapping genes with concomitant chromatin opening showed significant enrichment in immune-related pathways, including the chemokine signaling pathway, the PD-1 checkpoint pathway, and T cell receptor signaling (Fig. 2O).

Collectively, these multi-omic results demonstrated that BT therapy led to a multi-level enhancement of antitumor immunity in the tumor microenvironment. BT treatment not only reshaped the cellular composition of TILs and boosted T-cell functional gene expression but also established an epigenetic landscape conducive to robust immune activation. In summary, BT exerted broad immune regulatory effects, promoting a more immunologically active tumor microenvironment through coordinated changes at the cellular, molecular, and chromatin levels.

### Betrixaban suppresses LPS-induced inflammation

**Figure 3:**
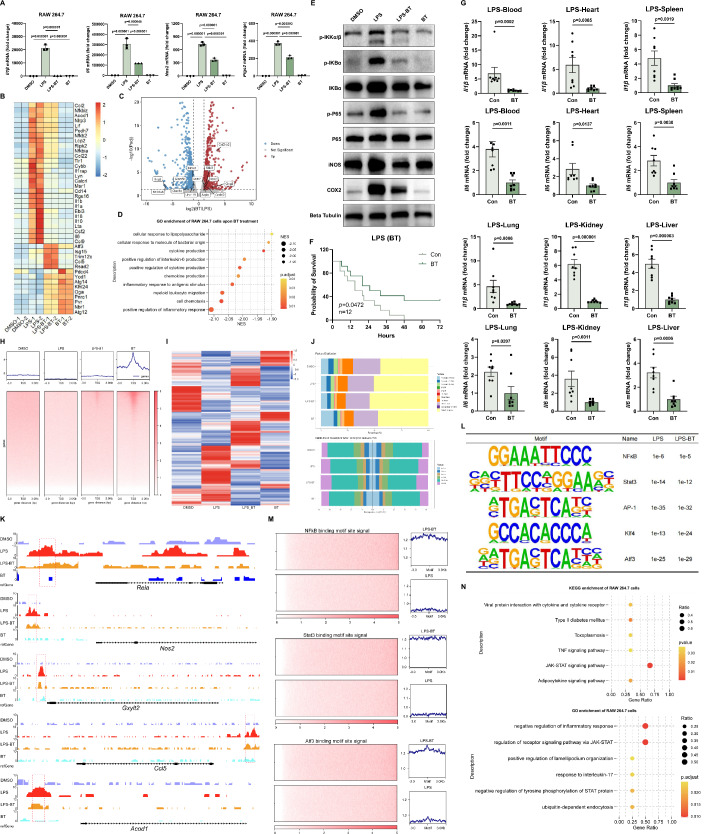
Betrixaban suppresses LPS-induced inflammation. (**A**) BT markedly downregulated the expression of *Il1β*, *Il-6*, *Nos2*, *Ptgs2* mRNA in RAW264.7 cells, following treatment with DMSO, LPS, LPS-BT, and BT alone. Ordinary one-way ANOVA test (*n* = 3). (**B**) Heatmap illustrated the expression of regulated genes in the BT group compared to DMSO groups, specifically those involved in the inflammatory response pathway. (**C**) Volcano plot depicted the distribution of DEGs between the treatment of LPS with BT or LPS alone in RAW264.7 cells. Wald test (*n* = 2). (**D**) Gene Ontology (GO) enrichment of differentially expressed genes (DEGs) between the treatment of LPS with BT or LPS alone in RAW264.7 cells. (**E**) BT significantly reduced the expression of p-IKKα/β, p-IκBα, t- IκBα, p-P65, t-P65, iNOS, COX2 at the protein level. Quantitative data are shown in Appendix Fig. S1. (**F**) BT significantly decreased the mortality rate in mice (*n* = 12) following LPS challenge. Log-rank (Mantel–Cox) test (*n* = 12). (**G**) RT-qPCR quantification of *Il1β* and *Il-6* in blood, heart, spleen, lung, kidney, liver. Unpaired *t* test (*n* = 8) (**H**) Heatmap showed that BT markedly changed the chromatin accessibility induced by LPS. (**I**) Heatmap illustrated the differentially accessible regions (DARs) most prominent in the LPS group compared to other groups. (**J**) Distribution of peaks and transcription factor-binding loci relative to transcription start sites (TSS) in the LPS, LPS-BT, and BT groups compared to the DMSO group, respectively, as annotated using the ChIPseeker R package. (**K**) Visualization of peak tracks for the DMSO, LPS, LPS-BT, and BT groups using IGV software. (**L**) Motif analysis obtained from the JASPAR database. (**M**) Mean aggregate binding signal of NF-κB, Stat3, and Atf3 across the LPS and LPS-BT groups. (**N**) GO and KEGG enrichment analysis of overlap genes in ATAC-seq and bulk RNA-seq between LPS and LPS-BT groups in RAW 264.7 cells. Data are shown as mean ± SEM. Source data are available online for this figure.

**Figure EV3:**
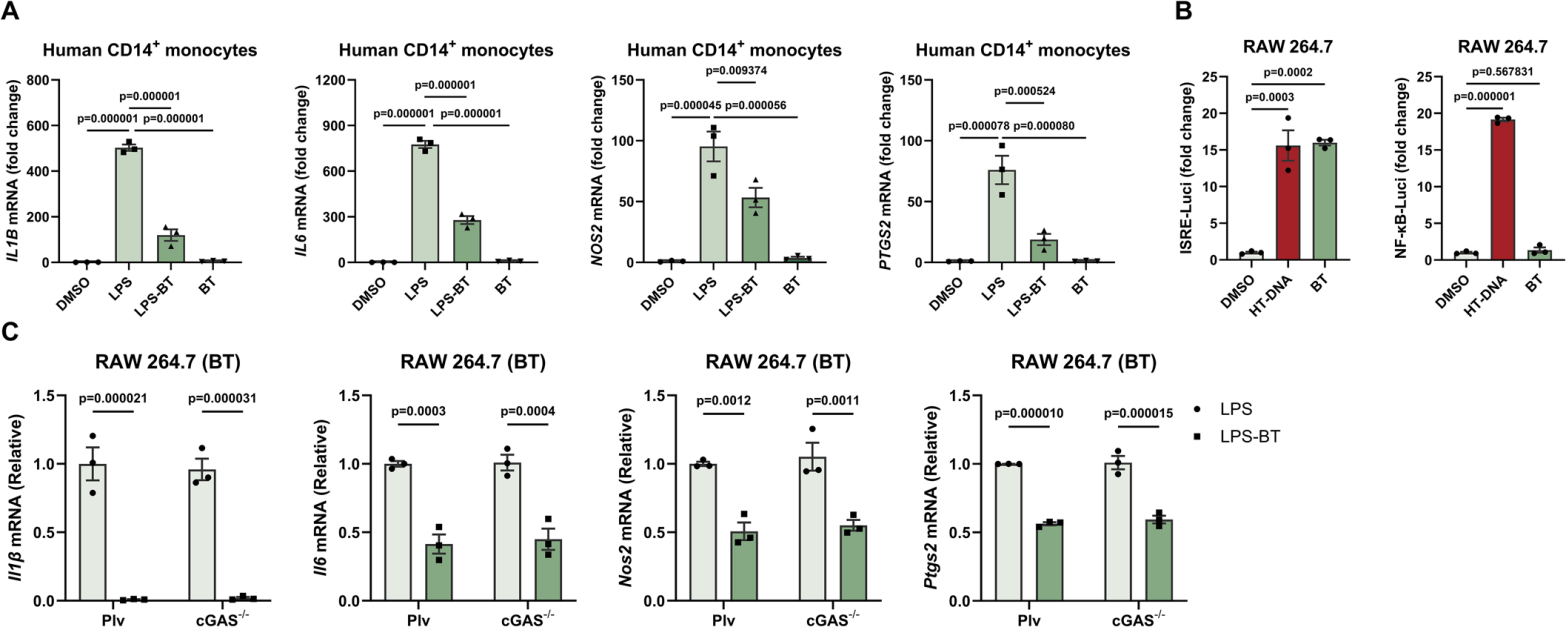
Betrixaban suppresses LPS-induced inflammation. (**A**) BT markedly downregulated the expression of *IL1B*, *IL6*, *NOS2*, *PTGS2* mRNA in human CD14^+^ monocyte cells, following treatment with DMSO, LPS, LPS-BT, and BT alone. Ordinary one-way ANOVA test (*n* = 3). (**B**) Activation of the ISRE and NF-κB reporter systems was assessed by luciferase assay following treatment with HT-DNA and BT. Ordinary one-way ANOVA test (*n* = 3). (**C**) Expression of *Il1β*, *Il-6*, *Nos2*, and *Ptgs2* mRNA in either plv-treated or cGAS^−/−^ RAW264.7 cells, following LPS priming and subsequent BT treatment. Two-way ANOVA test (*n* = 3). Data are shown as mean ± SEM.

Given the broad immunomodulatory effects of BT observed in tumor models, we next determined whether BT could also suppress excessive inflammatory responses. We first examined its impact using an in vitro LPS-induced inflammation model. RAW264.7 macrophages were treated with DMSO, LPS, LPS + BT, or BT alone. RT-qPCR analysis showed that LPS stimulation robustly upregulated the pro‑inflammatory cytokines *Il6* and *Il1b*, as well as the inflammatory mediator enzymes *Nos2* and *Ptgs2*, compared to DMSO controls (Fig. 3A). Co-treatment with BT significantly decreased the LPS-induced transcription of these genes. BT alone had little effect on the basal expression of these inflammatory markers. Consistent with these findings, BT co-treatment similarly reduced LPS-induced *IL6*, *IL1B*, *NOS2*, and *PTGS2* mRNA levels in human CD14⁺ monocytes, with minimal impact on baseline expression (Fig. EV3A). Thus, BT markedly attenuated LPS-triggered inflammatory gene induction at the mRNA level in macrophages. In line with these findings, stable RAW-ISRE and RAW-NF-κB reporter assays further showed that BT robustly induced ISRE activity while leaving NF-κB reporter activity near baseline, in contrast to cytosolic dsDNA (HT-DNA), which strongly activated both pathways (Fig. EV3B); importantly, BT also reduced LPS-induced inflammatory gene expression in cGAS-knockout RAW264.7 cells by RT-qPCR (Fig. EV3C).

To comprehensively investigate BT’s effects on LPS-driven gene expression in RAW264.7 cells, we performed bulk RNA-sequencing on each sample. LPS alone triggered a broad inflammatory transcriptional program, with hundreds of genes differentially expressed relative to control. BT co-treatment substantially altered this program. Heatmap visualization of differentially expressed genes (DEGs) indicated that the expression profile of BT co-treated cells was distinct from LPS alone, characterized by lower expression of inflammatory genes (Fig. 3B). The volcano plot of (LPS + BT vs LPS) revealed that a large number of genes were significantly regulated by BT, identifying 774 upregulated and 797 downregulated genes upon BT treatment (Fig. 3C). GO enrichment analysis highlighted multiple inflammation-related pathways among these DEGs (Fig. 3D).

Consistent with the transcriptional suppression, BT mitigated the increase of key inflammatory proteins and NF-κB activation caused by LPS. In LPS-stimulated RAW264.7 cells, Western blot analysis confirmed elevated protein levels of iNOS and COX-2, along with markedly enhanced phosphorylation of IKKα/β, IκBα, and NF-κB p65. BT treatment blocked these changes without affecting total protein levels of IκBα and NF-κB p65 (Fig. 3E). These protein-level findings corroborated the RT-qPCR results, confirming that BT inhibited LPS-induced inflammatory signaling in macrophages at both the mRNA and protein levels.

Encouraged by the in vitro findings, we next evaluated whether BT could ameliorate systemic inflammation in vivo using an LPS-induced sepsis model. Six-week-old C57BL/6 J mice received the pretreatment of a single dose of BT (50 mg/kg, i.p.) followed by a lethal intraperitoneal injection of LPS (20 mg/kg) 1 h later. All vehicle-treated mice died within 48 h, whereas nearly 40% of the BT-treated mice survived (Fig. 3F). To further assess systemic inflammation, we harvested blood, heart, spleen, lung, liver, and kidney tissues 12 h post-LPS injection and measured inflammatory cytokine mRNA levels. BT-treated mice exhibited significantly decreased levels of *Il1β*, *Il6* mRNAs across all tested tissues compared to controls, demonstrating effective suppression of systemic inflammation (Fig. 3G). These results indicated that BT effectively protected mice from hyperinflammation and endotoxic lethality, aligning with its potent anti-inflammatory action observed in vitro.

To explore the epigenetic basis of BT’s anti-inflammatory effects, we performed ATAC-seq to evaluate chromatin accessibility in RAW264.7 macrophages under the treatment of DMSO, LPS, BT + LPS, or BT alone. LPS stimulation alone caused widespread opening of chromatin at regulatory regions, reflecting the activation of inflammatory gene loci. Remarkably, BT co-treatment reversed a large portion of these changes, leading to reduced chromatin accessibility at multiple LPS-responsive loci (Fig. 3H–J). In particular, enhancers and promoters of key inflammatory genes, such as *Rela*, *Nos2*, *Gxylt2*, *Ccl5*, and *Acod1*, showed lower accessibility in BT co-treated cells compared to LPS alone, consistent with their suppressed transcription (Fig. 3K).

To identify upstream transcription factors potentially involved, we performed motif enrichment analysis on the regions exhibiting reduced accessibility with BT treatment. This analysis revealed significant enrichment for binding motifs of classical inflammation-associated transcription factors, including NF-κB, Stat3, AP-1, Klf4, and Atf3, in the BT-sensitive regions (Fig. 3L,M). This suggested that BT impaired the recruitment or activity of these key inflammatory TFs at their target sites, thereby limiting transcriptional activation of downstream inflammatory genes. Collectively, the ATAC-seq and motif analysis indicated that BT suppressed LPS-driven gene activation by restricting chromatin accessibility at pro-inflammatory loci and by dampening the activity of pivotal inflammatory transcription factors.

Finally, we integrated the transcriptomic and epigenomic datasets to directly link BT-induced changes in gene expression to alterations in chromatin state. We identified genes significantly downregulated at the mRNA level that concurrently showed reduced promoter accessibility based on ATAC-seq analysis. KEGG pathway analysis and GO analysis of these overlapping genes revealed strong enrichment for inflammatory pathways, including TNF and JAK-STAT signaling pathways, that were concordantly suppressed by BT at both the chromatin and transcriptional levels (Fig. 3N). This integrated multi-omics evidence firmly reinforced the conclusion that BT targeted key regulators of the inflammatory response.

Together, our results demonstrated that BT possessed potent anti-inflammatory activity, broadly suppressing LPS-induced inflammation through coordinated transcriptional repression and epigenetic modulation of critical inflammatory pathways.

### Betrixaban directly activates cGAS via a noncanonical mechanism

Building on our previous finding by surface plasmon resonance (SPR) that BT directly bound cGAS, we next examined whether BT directly stimulated cGAS enzymatic activity in vitro. We expressed and purified wild-type cGAS (residues 157–522) and four mutants, including a catalytic-site double mutant (E225A/D227A), a catalytic-triad mutant (E225A/D227A/D319A), a Zn^2+^-thumb mutant (C396A/C397A), and a DNA-binding-interface mutant (K173A/L174 A/K347A) with an N-terminal deletion. Each purified protein was incubated with BT (or DMSO) in the presence of ATP, GTP, and MgCl_2_, with or without exogenous double-stranded DNA (dsDNA), and the production of 2′3′-cGAMP was quantified by LC-MS.

**Figure 4:**
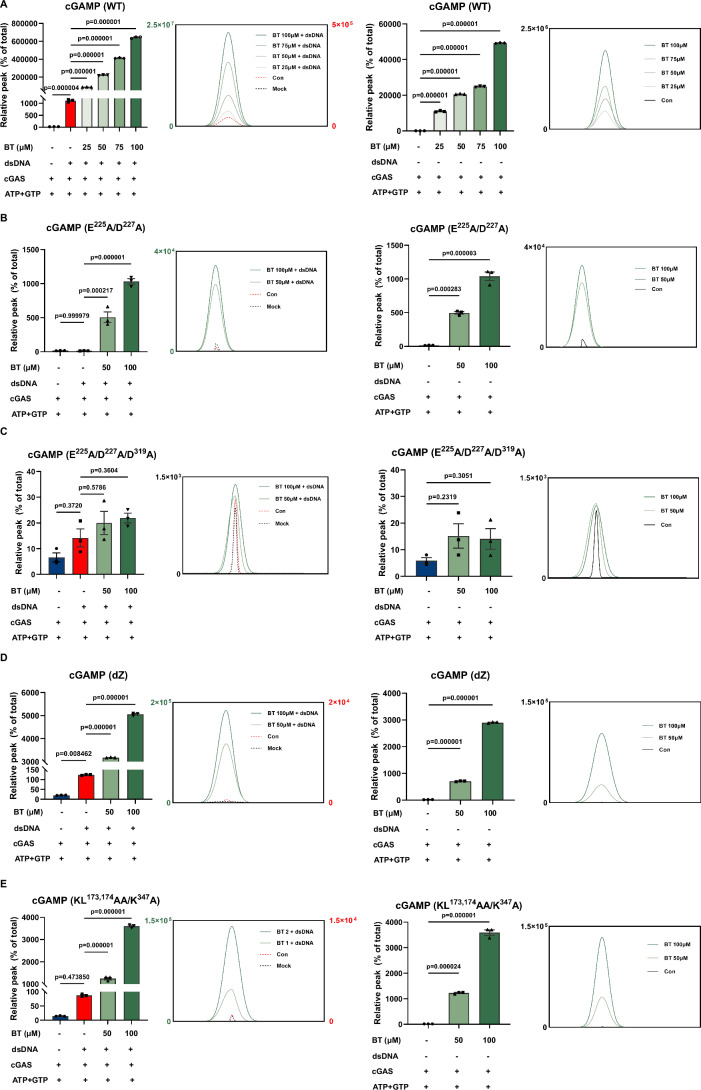
Betrixaban directly activates cGAS via a noncanonical mechanism. (**A**) In vitro cGAS enzymatic assays: LC-MS quantification of cGAMP production by wild-type human cGAS (residues 157–522) incubated with dsDNA in the presence of BT (25, 50, 75, or 100 µM) or the absence of exogenous DNA. Unpaired *t* test (*n* = 3). (**B**–**E**) In vitro cGAS enzymatic assays: LC-MS quantification of cGAMP production by human cGAS mutants incubated with dsDNA in the presence of BT (50 or 100 µM) or the absence of exogenous DNA. The mutants included a catalytic-site double mutant (E225A/D227A) (**B**), a catalytic-triad mutant (E225A/D227A/D319A) (**C**), a Zn^2+^-thumb mutant (C396A/C397A) (**D**), and a DNA-binding-interface mutant (K173A/L174A/K347A) (**E**) with an N-terminal deletion. Unpaired *t* test (*n* = 3). Data are shown as mean ± SEM. Source data are available online for this figure.

In wild-type cGAS, dsDNA robustly induced cGAMP synthesis, and addition of BT further increased the dsDNA-driven cGAMP yield compared with dsDNA alone. Notably, BT also activated wild-type cGAS in the absence of exogenous dsDNA, producing a measurable amount of cGAMP (Fig. 4A). This pattern indicated that BT did not replace DNA, but rather provided additional activation both in the absence and presence of dsDNA. As expected, all mutants generated negligible cGAMP in response to dsDNA. However, BT alone still elicited low but detectable cGAMP across the Zn^2+^-thumb and DNA-binding-interface mutants. For the catalytic triple mutant, the response to BT was abolished (Fig. 4B–E). These results demonstrated that BT directly activated cGAS through a noncanonical mechanism that can support cGAMP production in the absence of exogenous dsDNA and does not rely solely on canonical DNA binding or Zn²⁺ coordination.

**Figure EV4:**
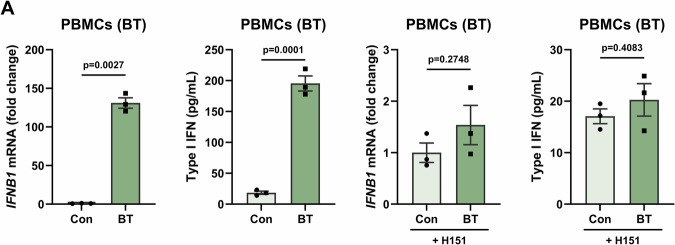
Betrixaban directly activates cGAS via a noncanonical mechanism. (**A**) PBMCs were assessed by RT-qPCR for *IFNB1* mRNA expression and by ELISA for Type I IFN production following BT or DMSO (Con) treatment, with or without H151 pretreatment. Unpaired *t* test (*n* = 3). Data are shown as mean ± SEM.

To assess whether BT also engaged the cGAS-STING pathway in primary human immune cells, we examined peripheral blood mononuclear cells (PBMCs) from healthy donors. BT stimulation significantly increased *IFNB1* mRNA expression in PBMCs and elevated IFN-β secretion into the culture supernatant. Pre-treatment with the cGAS inhibitor H-151 markedly attenuated these BT-induced responses, reducing both *IFNB1* transcript levels and IFN-β production to near-baseline values (Fig. EV4A). These findings indicated that BT activated the type I interferon program in primary human immune cells in a cGAS-dependent manner, providing a cellular correlate to its direct biochemical activation of human cGAS.

BT’s activation likely involved a binding site distinct from the canonical DNA-binding interface, which may help explain its ability to induce type I interferon (IFN-I) while limiting the induction of pro-inflammatory cytokines. Compared with classical activation by cytosolic DNA, which triggers both antiviral and pro-inflammatory cytokines, BT appeared to bias the cGAS-STING pathway toward antiviral signaling with relatively restrained inflammatory responses in our models. This selective modulation suggests that the pathway can be pharmacologically tuned to elicit protective immunity without pathological inflammation, providing a potential strategy for immunotherapies with strong antitumor activity and reduced inflammatory toxicity.

## Discussion

In summary, our study revealed that Betrixaban (BT) functioned as a dual immunomodulator that enhanced antitumor immunity while simultaneously restraining excessive inflammation. BT treatment significantly strengthened tumor-targeted immune responses in mice, as evidenced by reduced tumor growth and increased infiltration and activation of CD8⁺ T cells. These changes translated into improved efficacy of PD-1 checkpoint blockade when combined with BT. At the same time, BT prevented hyperinflammatory damage in a lipopolysaccharide (LPS) induced sepsis mouse model, markedly suppressing proinflammatory cytokine production and improving survival compared to controls. Together, these findings addressed a central challenge in immunotherapy: how to amplify protective immune responses against cancer without inducing pathological inflammation. Conventional approaches that activated innate immunity, such as systemic STING agonists, often triggered uncontrolled NF-κB-driven cytokine release syndromes. By contrast, BT achieved a more balanced immune stimulation, highlighting its significance as a single agent capable of promoting antitumor immunity while mitigating inflammatory toxicity.

Our findings suggested that BT’s mechanism differed from that of current immunotherapies and conferred potential advantages. Robust type I interferon (IFN-I) responses are known to be crucial for effective cancer immunity (Dufva et al, 2023; Liang et al, 2021). IFN-I activates dendritic cells (DCs), boosts antigen presentation, and enhances CD8⁺ T-cell priming, thereby improving responses to therapies like PD-1 blockade (Chae et al, 2022; Sanlorenzo et al, 2025; Yuan et al, 2025). Indeed, BT-treated tumors displayed characteristic features of enhanced IFN-I signaling and T cell activation, consistent with these intended effects. Notably, prior studies with direct STING agonists, such as CDNs or small molecules, reported potent antitumor IFN responses but at the cost of broad inflammation and systemic cytokine release when administered freely (Liang et al, 2022; Nguyen et al, 2024; Yang et al, 2025). Strategies to overcome such toxicity, for instance, intratumoral injection or nanoparticle encapsulation of STING agonists, underscore the difficulty of achieving systemic immune activation without collateral damage (Choi et al, 2024; Li et al, 2020; Wang et al, 2024). In contrast, BT’s dual mode of action inherently limited inflammatory cytokines even as it boosted IFN-I, eliminating the need for additional anti-inflammatory measures. This pointed to a novel therapeutic paradigm: rather than combining pro- and anti-inflammatory agents, a single repurposed drug BT can intrinsically calibrate the immune response. To our knowledge, this is the first demonstration that a Factor Xa inhibitor, originally designed as an anticoagulant, can be repurposed to invoke innate immune sensing in tumors. The therapeutic novelty of BT arose from both its mechanism and its outcome. In our study, BT engaged the cGAS-STING pathway through a noncanonical route. As a result, it enhanced T cell-mediated tumor clearance while simultaneously suppressing pathological inflammation. This combination has not been achieved by current immunotherapies.

Mechanistically, BT’s ability to activate the cGAS-STING pathway even in the absence of exogenous dsDNA, while further enhancing DNA-driven activation, was a striking finding of this study. Canonical cGAS activation required binding to cytosolic double-stranded DNA (dsDNA), which induced a conformational switch enabling cGAMP synthesis and subsequent STING activation (Gao et al, 2013; Li et al, 2013). BT partially bypassed this requirement, we observed that BT directly stimulated cGAS enzymatic activity even in the absence of DNA and in mutant cGAS proteins with disrupted DNA-binding. These biochemical results indicated that BT bound and directly activated cGAS through a nontraditional interface, partially mimicking the activation normally induced by DNA. Interestingly, few examples of such noncanonical cGAS activation have been reported; for instance, high concentrations of Mn^2+^ can directly activate cGAS without DNA (Lv et al, 2020; Zhang et al, 2021). BT now represented a pharmacological example of this principle, acting as an agonist of cGAS. The downstream consequences of BT’s cGAS activation were notably selective. In contrast to DNA-induced cGAS signaling, which robustly triggers both IRF3-mediated type I IFN and NF-κB-mediated inflammatory cytokines (Messaoud-Nacer et al, 2022). BT’s activation of the pathway was biased. The production of cGAMP by BT-activated cGAS led to STING-driven induction of interferon-stimulated genes, yet proinflammatory gene induction was blunted. This suggested that BT induced a subset of STING’s signaling outputs, sufficient to drive an IFN-I-dominated program, but insufficient to fully launch the canonical inflammatory cascade. Such selective tuning of cGAS-STING signaling is mechanistically intriguing, as it uncouples the beneficial antiviral/antitumor interferon response from the deleterious inflammatory response. Our findings therefore not only unveiled a new molecular mechanism for cGAS activation but also illustrated a novel strategy to pharmacologically modulate innate immunity with greater precision than traditional agonists.

The capacity of BT to balance interferon activation and inflammation has broad implications for immunotherapy. Type I interferons are well established for their role in promoting adaptive immunity against tumors, they activate dendritic cells for cross-priming and enhance cytotoxic T lymphocyte responses (Corrales et al, 2016; Hoover et al, 2024). We observed these effects in BT-treated tumors, which contained more activated CD8^+^ T cells producing IFN-γ and other effector cytokines. Concurrently, BT downregulated *IL-1β*, *IL-6*, *TNF-α*, and other inflammatory mediators. This dual action was critical because excessive inflammatory cytokines can lead to immune dysfunction, tissue damage, and even immunosuppression in the tumor milieu. For example, overactivation of NF-κB-dependent cytokines is associated with toxic cytokine release syndromes and can undermine antitumor immunity (Di et al, 2024). By limiting these factors, BT likely prevented the negative feedback loops and immune exhaustion that often accompany chronic inflammation. In the context of our LPS-induced sepsis model, the interferon and inflammasome balance achieved by BT proved life-saving. BT-treated mice had significantly improved survival and reduced organ cytokine levels compared to controls. This outcome underscored the importance of calibrating innate immune responses; a moderate IFN-I response can confer protection and initiate adaptive immunity, whereas unchecked inflammatory responses are fatal. Our work thus exemplified how tipping the cGAS-STING axis toward interferon signaling and away from rampant inflammation can yield therapeutic benefit. Achieving this balance is particularly important in cancer therapy, where one seeks to stimulate the immune system to attack tumors vigorously, yet must avoid inducing systemic inflammation or autoimmunity (De Martin et al, 2025). BT’s mechanism offers a blueprint for how an immunomodulator can “dial up” interferon-mediated immunity while “dialing down” inflammatory damage, a principle that could be applied to design safer immune stimulants for both oncology and inflammatory diseases.

While our study highlights a promising therapeutic approach, several limitations and future directions are worth discussing. First, our findings were demonstrated in mouse models and in vitro systems; it remained to be determined how to translate to human biology. Species-specific differences in the cGAS-STING pathway need to be carefully addressed when evaluating BT in clinical settings, as seen with some STING agonists that work in mice but not humans (Pan et al, 2020; Wang et al, 2020). In this study, we also observed BT-induced IFN-I responses in primary human PBMCs, but future work will be required to determine how BT modulates diverse human immune cell subsets and tumor–immune interactions in more physiological settings. Second, BT’s clinical dosing and safety as an immunomodulator will require optimization. The doses of BT effective for antitumor immunity in mice are higher than those used for anticoagulation in humans, and extended use of BT could carry bleeding risks inherent to Factor Xa inhibition. Thus, future studies should explore dose-ranging and schedule optimization to find a therapeutic window that maximizes immune benefits while minimizing anticoagulant side effects. It may also be feasible to modify BT’s structure to reduce its anticoagulant activity but preserve cGAS-binding, thereby improving safety, a strategy analogous to developing derivatives of existing drugs for new targets. Third, the mechanistic details of how BT exerts epigenetic and transcriptional effects deserve further investigation. We observed that BT treatment led to reduced chromatin accessibility at promoters of key inflammatory genes in LPS-challenged macrophages, suggesting BT interfered with the recruitment of inflammatory transcription factors to DNA. Identifying the upstream targets of BT responsible for this epigenetic modulation could reveal additional druggable nodes to suppress inflammation. Fourth, although our biochemical assays, cGAS-deficient cells, and tumor-intrinsic and host-intrinsic cGAS-knockout models collectively support a cGAS-dependent mechanism, the precise structural basis of BT-cGAS recognition remains unclear. Our current data indicate that BT functioned as a noncanonical activator of cGAS, but they did not yet pinpoint the exact binding interface at atomic resolution. High-resolution structural studies of cGAS in complex with BT, together with more extensive mutagenesis, will therefore be required to map the BT-binding surface and to fully elucidate how BT biases cGAS-STING signaling towards IFN-I-dominated responses with limited NF-κB activation.

In conclusion, this study demonstrated a novel strategy to engage the immune system against cancer: using a noncanonical cGAS activator to drive beneficial type I IFN responses while limiting harmful inflammation. BT exemplified this strategy, acting dually to promote CD8^+^ T cell-mediated tumor immunity and to protect against hyperinflammatory injury. These findings provide a foundation for developing improved immunotherapies that are both potent and safe, and they pave the way for clinical translation of BT as an immunomodulatory agent with a uniquely balanced action profile. The dual enhancement of antitumor immunity and suppression of pathological inflammation by BT underscores a therapeutic paradigm that could significantly advance cancer immunotherapy and immune regulation at large.

## Methods

**Table:** Reagents and tools table

Reagent/resource	Reference or source	Identifier or catalog number
**Experimental models**		
C57BL6/J (*M*. *musculus*)	Department of Laboratory Animal Science, Peking University Health Science Center	N/A
C57BL6/J (*M.* *musculus*) cGAS^-/-^	Dr. Zhengfan Jiang	Peking University
RAW 264.7 (*M.* *musculus*)	ATCC	RRID: CVCL_0493
hPBMC (*H. sapiens*)	YAYUBIO	N/A
B16F10 (*M.* *musculus*)	ATCC	RRID: CVCL_0159
Pan02 (*M. musculus*)	ATCC	RRID: CVCL_D627
**Antibodies**		
Rabbit anti-COX2 (1:1000 dilution)	Wanleibio	WL01750
Rabbit anti-Beta Tubulin (1:4000 dilution)	Proteintech	10094-1-AP
Rabbit anti-iNOS (1:2000 dilution)	Proteintech	80517-1-RR
Goat Anti-Rabbit IgG (1:8000 dilution)	Proteintech	SA00001-2
Goat Anti-Mouse IgG (1:8000 dilution)	Proteintech	SA00001-1
Rabbit anti-p-NF-κB p65 (Ser536) (1:1000 dilution)	Cell Signaling Technology	3033
Rabbit anti-NF-κB p65 (1:1000 dilution)	Cell Signaling Technology	8242
Rabbit anti-p-IκBα (Ser32) (1:1000 dilution)	Cell Signaling Technology	2859
Rabbit anti-IκBα (1:1000 dilution)	Cell Signaling Technology	4812
Rabbit anti-p-IKKα (Ser176)/IKKβ (Ser177) (1:1000 dilution)	Cell Signaling Technology	2078
Anti-mouse PD-1 (CD279)	Selleck	A2122
PE/Cyanine7 anti-mouse CD45	BioLegend	157205
FITC anti-mouse CD4	BioLegend	100405
APC anti-mouse CD8a	BioLegend	100711
PE anti-mouse CD62L	BioLegend	161204
PerCP/Cyanine5.5 anti-mouse CD69	BioLegend	104521
PerCP anti-mouse/human CD44	BioLegend	103036
APC anti-mouse IFN-γ	BioLegend	505809
PE anti-mouse TNF-α	BioLegend	506305
PE anti-mouse CD80	BioLegend	600055
APC anti-mouse CD86	BioLegend	159215
FITC anti-mouse NK-1.1	BioLegend	156507
Rat IgG2a isotype	BioXCell	BE0089
**Oligonucleotides and other sequence-based reagents**		
RT-qPCR primers	This study	Table EV1
**Chemicals, enzymes, and other reagents**		
Betrixaban	MedChemExpress	HY-10268
Puromycin	Sigma-Aldrich	P4512
Lipopolysaccharides, from E. coli O55:B5	MedChemExpress	HY-D1056
Dimethyl sulfoxide	Solarbio	D8371
DMEM	EallBio	03.1002 C
Collagenase D	Roche	11088866001
DNase I	Sigma-Aldrich	DN25-100G
Luciferase assay kit	TransGen Biotech	FR203-01
HiScript II RT SuperMix	Vazyme	R223-01
SYBR Green qMix	Vazyme	Q311
D-Luciferin	MedChemExpress	HY-12591A
RIPA Lysis Buffer (Strong)	MedChemExpress	HY-K1001
EDTA-Free Protease Inhibitor Cocktail	MedChemExpress	HY-K0010
Phosphatase Inhibitor Cocktail II	MedChemExpress	HY-K0022
Phosphatase Inhibitor Cocktail III	MedChemExpress	HY-K0023
Nitrocellulose membrane	Beyotime	FFN08
Enhanced chemiluminescence	EallBio	07.10009-50
High-throughput RNA extraction kit	TIANGEN	A0123A01
Hyperactive ATAC-Seq Library Prep Kit	Vazyme Biotech	TD711
**Software**		
ImageJ	https://imagej.net/ij/index.html	N/A
GraphPad Prism 10	www.graphpad-prism.cn	N/A
DESeq2 R package (v1.38.3)	https://bioconductor.org/packages//release/bioc/html/DESeq2.html	N/A
Trim-Galore (v0.6.4)	https://www.bioinformatics.babraham.ac.uk/projects/trim_galore/	N/A
Bowtie2 aligner (v2.3.5.1)	https://github.com/BenLangmead/bowtie2	N/A
Samtools (v1.10)	https://sourceforge.net/projects/samtools/files/samtools/1.10/	N/A
MACS3 (v3.0.0a5)	https://github.com/macs3-project/MACS/releases/tag/v3.0.0	N/A
Bedtools merge (v2.31.1)	https://github.com/arq5x/bedtools2/releases	N/A
BamCoverage (v3.3.2)	https://deeptools.readthedocs.io/en/latest/content/tools/bamCoverage.html	N/A
Csaw R package (v1.38.0)	https://new.bioconductor.org/packages/release/bioc/vignettes/csaw/inst/doc/csaw.html	N/A
ChIP seeker R package (v1.34.1)	https://bioc.r-universe.dev/ChIPseeker/doc/ChIPseeker.html	N/A
Integrative Genomics Viewer (IGV) (v2.17.4)	https://igv.org/doc/desktop/#DownloadPage/	N/A
**Other**		
Flow cytometer	BD Biosciences	N/A
Cryostat Microtome	Leica	N/A
Fluorescence microscope	Nikon	N/A
TD20/20 Luminometer	Turner Designs	N/A
High-pressure homogenization	Union-Biotech	N/A
Ni-NTA column	Qiagen	N/A
Superdex 200 10/300 GL column	Cytiva	N/A

### Reagents and antibodies

Rabbit anti-COX2 antibodies were purchased from Wanleibio. Rabbit antibodies against iNOS (catalog no. 80517-1-RR) and Beta Tubulin (catalog no. 10094-1-AP) were procured from Proteintech. Additional antibodies, including rabbit phospho-NF-κB p65 (Ser536) (93H1) (catalog no. 3033), NF-κB p65 (D14E12) XP (catalog no. 8242), phospho-IκBα (Ser32) (14D4) (catalog no. 2859), IκBα (44D4) (catalog no. 4812), and phospho-IKKα (Ser176)/IKKβ (Ser177) (C84E11) (catalog no. 2078), were obtained from Cell Signaling Technology. Secondary detection utilized HRP-conjugated Affinipure Goat Anti-Rabbit IgG (H + L) (catalog no. SA00001-2) and HRP-conjugated Affinipure Goat Anti-Mouse IgG (H + L) (catalog no. SA00001-1), also from Proteintech. Anti-mouse PD-1 (CD279) (catalog no. A2122) and Mouse CD8^+^ T Cell Sorting Kit (catalog no. B90011) were purchased from Selleck. Fluorochrome-conjugated flow cytometry antibodies, including PE/Cyanine7 anti-mouse CD45 Antibody (catalog no. 157205), FITC anti-mouse CD4 Antibody (catalog no. 100405), APC anti-mouse CD8a Antibody (catalog no. 100711), PE anti-mouse CD62L Antibody (catalog no. 161204), PerCP/Cyanine5.5 anti-mouse CD69 Antibody (catalog no. 104521), PerCP anti-mouse/human CD44 Antibody (catalog no. 103036), APC anti-mouse IFN-γ Antibody (catalog no. 505809), PE anti-mouse TNF-α Antibody (catalog no. 506305), PE anti-mouse CD80 Antibody (catalog no. 600055), APC anti-mouse CD86 Antibody (catalog no. 159215) and FITC anti-mouse NK-1.1 Antibody (catalog no. 156507) were bought from BioLegend. Lipopolysaccharides, from E. coli O55:B5 (catalog no. HY-D1056) and Betrixaban (catalog no. HY-10268) were sourced from MedChemExpress. Dimethyl sulfoxide (DMSO) (catalog no. D8371) was purchased from Solarbio.

### Cells

The RAW264.7 (RRID: CVCL_0493) cell was sourced from the American Type Culture Collection (ATCC), and maintained in Dulbecco’s Modified Eagle’s Medium (DMEM) (03.1002C, EallBio). Culture media were supplemented with 10% fetal bovine serum (FBS) and 1% penicillin–streptomycin for optimal cell growth and maintenance. In addition, cell identity was verified by short tandem repeat (STR) profiling, and routine mycoplasma screening with Mycolor One-Step Mycoplasma Detector (Vazyme, D201-01) was consistently negative.

Human peripheral blood mononuclear cells (PBMCs) were bought from Shanghai Yayu Biotechnology Co., Ltd (YAYUBIO, Shanghai, China). CD14^+^ monocytes were isolated from PBMCs by magnetic bead-based positive selection (Selleck) according to the manufacturer’s instructions and were used immediately for stimulation assays. Collection of peripheral blood from healthy adult donors and subsequent use of PBMCs were approved by the Institutional Review Board of Shanghai Liquan Hospital (protocol number 2022-09). Peripheral blood was obtained from three healthy adult Chinese donors (two males, 21 and 31 years old, and one female, 25 years old).

### CRISPR/Cas9 gene editing

The lentivirus-mediated CRISPR/Cas9 technology was used to generate cGAS knockout Pan02 cells. Briefly, the highly efficient and specific guide RNAs (sgRNAs) targeting the gene of interest were designed and selected by using the online CRISPR design website. The oligonucleotide pairs encoding sgRNAs were synthesized, annealed, and inserted into the lentiCRISPR v2 vector that had been digested by the BsmBI enzyme (NEB). For lentivirus production, the transfection mixture of the constructed lentiCRISPR v2 vector (2400 ng), packaging plasmid psPAX2 (800 ng), envelope plasmid VSV-G (800 ng), and PEI (1600 ng) was added into 293T cells and incubated at 37 °C for 72 h. The supernatant containing the virus was then collected and added to Pan02 cells supplemented with polybrene (Beyotime Biotechnology, China). At 48 h post-infection, cells were cultured in fresh media containing 3 µg/mL puromycin (Sigma-Aldrich, USA) for stable selection of transduced cells. The successful knockout cell line was validated by Sanger sequencing.

### Mice

Wild-type (WT) C57BL/6J mice were purchased from the Department of Laboratory Animal Science, Peking University Health Science Center. cGAS knockout mice, all on a C57BL/6J background, were gifts from Zhengfan Jiang (Peking University).

All animal care and procedures were conducted in accordance with the Guide for the Care and Use of Laboratory Animals by the Chinese Association for Laboratory Animal Science. The protocols were approved by the Animal Care Committee of Peking University Health Science Center (permit number: LA 2016240). Mice were bred and housed under specific pathogen-free conditions at the Laboratory Animal Center of Peking University. Only mice aged 6–8 weeks were used in the experiments.

### Construction of a sepsis model via LPS injection

Sepsis induced by lipopolysaccharide (LPS) was used to model systemic inflammatory responses. Male C57BL/6J mice aged 6–8 weeks and weighing 20–25 g were randomly assigned to two groups with twelve animals per group, an LPS plus PBS group and an LPS plus BT group. LPS at 20 mg/kg in sterile saline was delivered by intraperitoneal injection using sterile insulin syringes. Immediately after LPS administration, mice in the BT group received an intraperitoneal dose of 100 μL BT (50 mg/kg), while control mice received an equal volume of PBS. Survival was monitored for 48 h, and blood and tissues were collected 12 h after LPS administration for downstream analyses.

We deliberately used a high dose of LPS (20 mg/kg, i.p.) as a lethal sepsis model, rather than as a model of mild or physiological inflammation. Mice are relatively insensitive to LPS, and similar high doses are widely used to reproducibly induce systemic inflammatory response syndrome, cytokine storm, multi-organ inflammation, and rapid mortality, thereby modeling the extreme hyperinflammatory state of severe sepsis or cytokine storm rather than everyday inflammatory conditions. The BT dosing regimen used in this study was previously reported to be well tolerated in mice without significant changes (Chen et al, 2025).

### Tumor transplantation and treatments

Mice were subcutaneously injected into the right flank with 5 × 10⁵ Pan02 cells or B16F10 cells suspended in 100 μL PBS, unless otherwise specified. Tumor growth was monitored daily, with tumor size measured every 2–3 days. Tumor volume was calculated using the formula: volume = length (mm) × width² (mm²) × 0.5. Tumor-bearing mice were treated with 50 mg/kg BT or the same volume of PBS via intraperitoneal injection every 2 days. For immune checkpoint blockade, tumor-bearing mice were administered 200 μg of anti-mouse PD-1 antibody (200 μL saline) via intraperitoneal injection on days 3, 7, 11, and 15 following tumor inoculation. Control mice received 200 μL of rat IgG2a isotype (Clone 2A3, BioXCell) on the same schedule.

### Tissue digestion and isolation of tumor-infiltrating leukocytes

Tumors were collected into cold PBS containing 1% heat-inactivated serum (FACS), then minced and digested in RPMI 1640 medium containing 0.5 mg/mL collagenase D (11088866001, Roche) and 0.1 mg/mL DNase I (DN25-100G, Sigma-Aldrich) at 37 °C for 1 h.

Digests were filtered through a 100-μm strainer into cold FACS buffer, further passed through a 200-μm mesh, and centrifuged at 1600 rpm for 5 min. Pellets were gently resuspended in Ficoll according to the manufacturer’s instructions and overlaid with serum-free medium. After centrifugation at 800 × *g* for 20 min with low acceleration and deceleration, the mononuclear cell layer at the interface was collected, washed with FACS buffer, and centrifuged at 1600 rpm for 5 min. Cells were resuspended in 80% Percoll. Where indicated, leukocytes were enriched on a discontinuous Percoll gradient by gently layering 40% Percoll above the cell suspension and serum-free medium on top. The gradient was centrifuged at 800 × *g* for 30 min at room temperature with low acceleration and deceleration. Cells at the interface between 80% and 40% Percoll were collected and washed with FACS buffer. Red blood cells were lysed in ACK lysis buffer for 2 min, followed by neutralization with FACS buffer and centrifugation. The resulting cell mixture was then filtered through a 70-μm cell strainer to obtain a single-cell suspension, and then the cells were counted. Only samples with cell viability above 90% proceeded to flow cytometry.

### Flow cytometry analysis

Single-cell suspensions were kept on ice. Cells were stained with fluorochrome-conjugated antibodies against surface markers in FACS buffer for 20–30 min in the dark, then washed and resuspended. For intracellular cytokines, cells were stimulated with PMA and ionomycin in the presence of brefeldin A, where indicated, fixed and permeabilized using standard buffers, and stained for IFN-γ and TNF-α. Data were acquired on a calibrated flow cytometer (BD Biosciences) using matched compensation controls, fluorescence-minus-one controls, and live-dead discrimination. Analyses were performed in FlowJo.

### Orthotopic KPC-luc pancreatic tumor model

Orthotopic pancreatic tumors were established using luciferase-expressing KPC (KPC-luc) cells in immunocompetent mice. KPC-luc cells were maintained in DMEM supplemented with 10% FBS, 1% penicillin–streptomycin, and 2 mM L-glutamine at 37 °C in a humidified 5% CO₂ incubator. For orthotopic implantation, cells were harvested at 70–80% confluence, washed twice with sterile PBS, and resuspended in ice-cold PBS/Matrigel (1:1, v/v) at a final concentration of 2 × 10⁷ cells/mL. Mice were anesthetized with isoflurane (3% for induction, 2% for maintenance in oxygen) and placed on a heating pad. After shaving and disinfecting the left flank, a 1–1.5 cm left subcostal incision was made to expose the spleen and underlying pancreatic tail.

Using a 29-G insulin syringe, 2 × 10⁵ KPC-luc cells in 50 μL PBS/Matrigel were slowly injected into the pancreatic tail. Successful implantation was confirmed by the formation of a visible bleb at the injection site without leakage into the peritoneal cavity. Mice in which obvious leakage occurred were excluded from the study. The pancreas and spleen were gently returned to the peritoneal cavity, the peritoneum and muscle were closed with absorbable sutures, and the skin was closed with wound clips or non-absorbable sutures. Mice received perioperative analgesia and were monitored daily for recovery, body weight, and signs of distress.

After implantation, tumor engraftment was verified by bioluminescence imaging, and mice with comparable baseline signals were randomized into treatment groups. BT was administered at 50 mg/kg by intraperitoneal injection once every other day. Control mice received PBS on the same schedule. At the experimental endpoint, mice were euthanized, and the pancreas with attached orthotopic tumor was dissected and weighed as a measure of tumor burden. Tumor tissue was snap-frozen for RNA extraction and RT-qPCR analysis.

### Bioluminescence imaging of orthotopic KPC-luc tumors

Tumor burden in the orthotopic KPC-luc model was monitored longitudinally by in vivo bioluminescence imaging (BLI). Mice were injected intraperitoneally with D-luciferin (150 mg/kg in PBS) and anesthetized with isoflurane. Ten minutes after D-luciferin administration, images were acquired using an IVIS Spectrum imaging system with standardized acquisition parameters applied to all groups within each experiment.

Bioluminescent signals were analyzed using Living Image software. A fixed region of interest (ROI) was placed over the pancreatic area for each mouse, and total photon flux (photons/sec) within the ROI was quantified. For longitudinal analysis, total flux values at subsequent time points were normalized to the baseline signal for each mouse. These measurements were used as a surrogate for orthotopic tumor burden and for comparing tumor growth kinetics between vehicle- and BT-treated groups.

### B16F10 experimental lung metastasis model

B16F10 cells were cultured in DMEM supplemented with 10% FBS. For tail-vein injection, subconfluent cultures were detached, washed with PBS, and resuspended in PBS at a final concentration of 1 × 10^5^ cells/100 μL. Cells were slowly injected into the lateral tail vein using a 29-G insulin syringe.

Mice were monitored daily for body weight, general condition, and signs of distress. BT was administered at 50 mg/kg by intraperitoneal injection once every other day, control mice received vehicle on the same schedule. At the experimental endpoint, mice were euthanized, and the lungs were weighed as a measure of metastatic burden. Visible black metastatic nodules on the lung surface were counted under a dissecting microscope in a blinded manner.

### Immunofluorescence staining of CD8⁺ T cells

Paraffin-embedded lung sections were deparaffinized and rehydrated through a graded alcohol series. Antigen retrieval was conducted in citrate buffer (pH 6.0) using a microwave oven. Sections were blocked in 5% BSA with 0.3% Triton X-100 for 1 h at room temperature, followed by overnight incubation at 4 °C with anti-mouse CD8α primary antibody. After washing, slides were incubated with Alexa Fluor 488-conjugated secondary antibody for 1 h at room temperature. Nuclei were counterstained with DAPI (1 μg/mL), and sections were mounted with antifade reagent. Fluorescent images were captured using a Nikon confocal microscope.

### Hematoxylin-eosin (H&E) staining

The tissues were quickly placed in cold saline solution and rinsed after they were collected, then fixed in 4% paraformaldehyde, dehydrated, and embedded in paraffin prior to sectioning at 5 mm, and sections were stained with hematoxylin and eosin.

### RNA extraction, reverse transcription, and quantitative PCR

Total RNA was extracted from cells after the indicated treatments or infections using TRIzol reagent from TIANGEN, catalog A0123A01. RNA was reverse transcribed to cDNA with HiScript II RT SuperMix from Vazyme, catalog R223-01. Target transcripts were quantified by real-time PCR using SYBR Green qMix from Vazyme, catalog Q311. Relative mRNA levels were normalized to Actb as the housekeeping gene. Primer sequences are listed in Table EV1.

### Luciferase assay

Cells were seeded into 12-well plates and incubated overnight. Cells were then treated with BT for 12 h at 37 °C in a humidified atmosphere with 5% CO2. After incubation, cells were lysed directly in the wells using lysis buffer provided in the luciferase assay kit (TransGen Biotech, cat. FR203-01), following the manufacturer’s instructions. The lysates were collected by gentle pipetting, and luciferase activity was measured using a TD20/20 Luminometer (Turner Designs).

### Total protein extraction and immunoblotting

Cells were lysed in RIPA lysis buffer, strong formulation, from MedChemExpress, catalog HY-K1001. The buffer contained an EDTA-free protease inhibitor cocktail and phosphatase inhibitor cocktails II and III at 1:100 in DMSO. Lysates were cleared by centrifugation, and protein concentration was measured. Equal amounts of protein, typically 10–30 μg, were separated by SDS–PAGE and transferred to nitrocellulose membranes from Beyotime, catalog FFN08. Membranes were blocked, incubated with primary antibodies, washed, and then incubated with HRP-conjugated secondary antibodies. Signals were developed by enhanced chemiluminescence using the ECL kit from EallBio, catalog 07.10009-50, and recorded on a digital imager.

### Expression and purification of cGAS truncations and mutations

cGAS truncations and mutations were PCR-amplified from plasmid pET28a + -cGAS, which carried the full-length human cGAS gene or its mutants, and cloned into the pET-28a+ vector optimized for *E. coli* expression of an N-terminal 6xHis-SUMO-TEV fusion protein. Recombinant proteins were expressed in ER2566 (WEIDI) at 16 °C after initial growth in LB medium at 37 °C for 8 h and subsequent induction with 0.5 mM IPTG at 16 °C for 14 h. Cells were harvested by centrifugation at 1914 × *g* for 10 min at 4 °C, resuspended in 25 mM Tris-HCl (pH 7.5), 20 mM imidazole, 500 mM NaCl, 3 mM β-mercaptoethanol, and 2 mM PMSF, and lysed by high-pressure homogenization (Union-Biotech) at 800 bar for 10 min at 4 °C. The lysate was clarified by centrifugation at 34,571 × *g* for 30 min, and the supernatant was applied to a Ni-NTA column (Qiagen) for 1 h at 4 °C. Bound protein was eluted with lysis buffer supplemented to 100 mM imidazole, the SUMO tag was removed by TEV protease digestion, and the best fractions were pooled, concentrated, and further purified by size-exclusion chromatography on a Superdex 200 10/300 GL column (Cytiva) in 50 mM Tris-HCl (pH 8.0), 300 mM NaCl.

### cGAS activity assay

Purified human cGAS (hcGAS) was incubated at 37 °C for 30 min in a 50 μL reaction that contained 1 μM hcGAS, 1 mM ATP, 1 mM GTP, 100 mM NaCl, 40 mM Tris-HCl at pH 7.5, and 10 mM MgCl₂. Where indicated, the reaction included double-stranded DNA at 5 μg/mL. Reactions were heated to 99 °C, then 200 μL acetonitrile was added to precipitate proteins. Samples were centrifuged at 14,000 rpm for 40 min, and the supernatants were analyzed by LC-MS/MS.

### LC-MS/MS quantification of 2′,3′-cGAMP

Mass spectrometry was performed on a Xevo TQ-S triple-quadrupole instrument with electrospray ionization in positive mode. Samples were separated on a reverse-phase C18 column with a mobile-phase flow rate of 0.3 mL/min. Mobile phase A was water with 0.1% formic acid, and mobile phase B was acetonitrile with 0.1% formic acid. The gradient started at 2% B for 1 min, increased linearly to 98% B over 4 min, was held for 1 min, and then returned to 2% B over 0.5 min Data were acquired in multiple-reaction-monitoring mode. The precursor ion at *m/z* 675.1 was monitored with two product ions: 675.1 → 136.0 for quantification and 675.1 → 152.0 for confirmation.

### RNA-seq and data analysis

Total RNA was extracted with the TIANGEN A0123A01 high-throughput kit. Quality control, library preparation, sequencing, and downstream analysis were performed by Suzhou GENEWIZ following their standard protocols. Gene counts were generated by featureCounts v2.0.0 and normalized to FPKM. Differential expression was assessed in DESeq2 v1.38.3 using |log₂FC | > 2 and *P *< 0.05.

### ATAC-seq and data analysis

We performed ATAC-seq using the Hyperactive ATAC-Seq Library Prep Kit (TD711, Vazyme Biotech). Tn5 transposase, preloaded with sequencing adapters, was incubated with isolated nuclei to insert adapters into open chromatin. After PCR amplification with indexed primers, libraries were sequenced by GENEWIZ (Suzhou, China), yielding a genome-wide map of chromatin accessibility.

Raw ATAC-seq reads (paired-end) from GENEWIZ were first trimmed with Trim-Galore v0.6.4, then aligned to the mouse mm10 genome using Bowtie2 v2.3.5.1 (--very-sensitive -X 2000) and piped through SAMtools v1.10 to produce sorted, indexed BAM files. Peaks were called for each sample with MACS3 v3.0.0a5 (default settings), yielding per-sample BED files.

For ATAC-seq, individual peak sets were merged across all samples with Bedtools v2.31.1, and genome-wide coverage tracks were generated in RPKM units using deepTools v3.3.2 (bamCoverage --binSize 100 --normalizeUsing RPKM --effectiveGenomeSize 2864785220 --ignoreForNormalization chrM --extendReads). Differential accessibility/enrichment analyses were performed in R using the csaw package v1.38.0, and peaks were annotated with ChIPseeker v1.34.1. Final track visualization was carried out in IGV v2.17.4 (Guo et al, 2025).

### Generation and processing of single-cell RNA sequencing data

Tumors were harvested after subcutaneous implantation and were gently dissociated to single-cell suspensions. The cell suspensions were transferred to Genewiz, Azenta Life Sciences. CD45-positive cells were isolated by flow cytometric sorting and were used for library preparation. Libraries were prepared with the 10x Genomics Chromium Next GEM Single Cell 3′ Reagent Kits v3.1 according to the manufacturer’s instructions. Sequencing was carried out on an Illumina NovaSeq 6000 in paired-end 150 base-pair mode. Genewiz delivered FASTQ files for analysis.

Raw reads were processed with Cell Ranger version 3.1.0 for barcode demultiplexing, alignment, and unique molecular identifier counting to generate feature-barcode matrices. Downstream analyses were performed in R with Seurat version 4.3.0. Data were normalized, subjected to dimensionality reduction, and clustered to define cell populations. Cell types were annotated manually based on canonical marker genes. Results were exported for subsequent analyses.

Cell–cell communication was assessed with the CellChat package version 1.1.3. Gene set enrichment analysis and gene set variation analysis were performed with clusterProfiler version 4.6.2. Pseudotime trajectories were inferred with Monocle3 version 1.0.0. Marker gene expression was visualized with dot plots, violin plots, and heatmaps (Guo et al, 2024).

### Quantification and statistical analysis

Statistical analyses were conducted using Student’s *t* test for two-group comparisons (indicated by square brackets) and one-way ANOVA for multi-group experiments. Survival was evaluated by Kaplan–Meier analysis. Regarding experimental design, animals (or samples) were randomly assigned to experimental groups. For in vivo experiments, randomization was performed using a computer-generated random sequence. For in vitro experiments, cell cultures were randomized across plates and positions. For each experiment, the number of biologically independent replicates (*n*) is indicated in the figure legends, with technical replicates averaged for each biological replicate. All experiments were repeated at least three times independently. Data are shown as mean ± SEM (*n* ≥ 3).

## Supplementary information

Table EV1

Appendix Figure S1

Peer Review File

Source data Fig. 1

Source data Fig. 2

Source data Fig. 3

Source data Fig. 4

Expanded View Figures

## Data Availability

The sequencing datasets produced in this study are available in the following databases: RNA-Seq data, ATAC-Seq data, and single-cell RNA-Seq data: Sequence Read Archive PRJNA1304132. The source data of this paper are collected in the following database record: biostudies:S-SCDT-10_1038-S44321-026-00429-1.
