# Supplementary material for: Betrixaban activates cGAS-STING to promote antitumor immunity without pathological inflammation
Source: EMBO Mol Med. 2026 May 14;18(6):2213–35. doi: 10.1038/s44321-026-00429-1 (PMC13269763; doi:10.1038/s44321-026-00429-1)
Supplement: Supplementary file 2 — Appendix Figure S1 [file 44321_2026_429_MOESM2_ESM.pdf]

Appendix for "Betrixaban Activates cGAS-STING to Promote Antitumor Immunity without Pathological Inflammation"

Table of Contents

1. Appendix Figure S1 2

Appendix Figure S1

A Related to Figure 3E

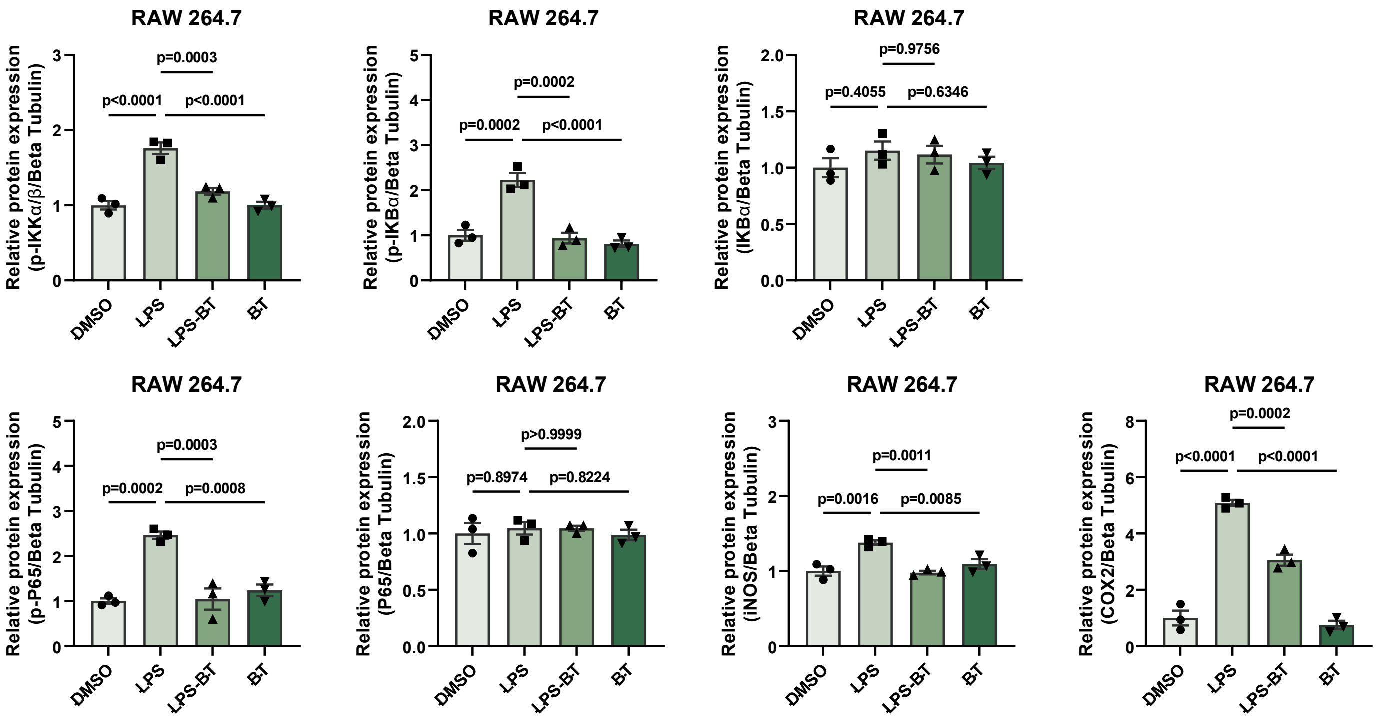

Appendix Figure S1. Quantification of the presented Western blots.

(A) quantification of band intensities in Figure 3E. Densitometry was performed in ImageJ 1.51 using fixed rectangular ROIs with local background subtraction. Target proteins were normalized to loading control and then to the vehicle group, which was set to 1.0. Data are shown as mean ± SEM (n = 3 independent experiments).
