# Supplementary figures and images for: Betrixaban activates cGAS-STING to promote antitumor immunity without pathological inflammation
Source: EMBO Mol Med. 2026 May 14;18(6):2213–35. doi: 10.1038/s44321-026-00429-1 (PMC13269763; doi:10.1038/s44321-026-00429-1)

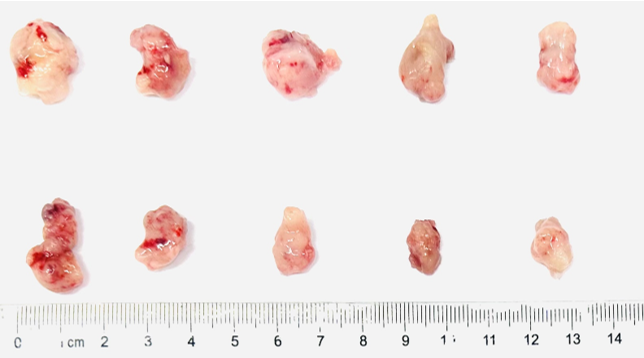

Supplement: Supplementary file 4 — Source data Fig. 1 [file 44321_2026_429_MOESM4_ESM.zip › Figure 1/1A/1A.png]

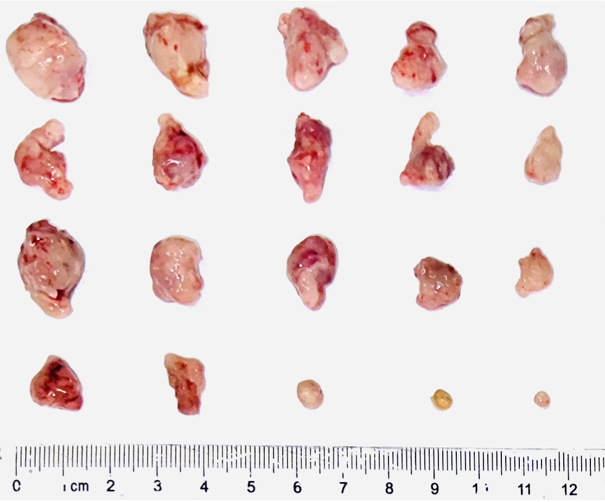

Supplement: Supplementary file 4 — Source data Fig. 1 [file 44321_2026_429_MOESM4_ESM.zip › Figure 1/1L/1P.png]

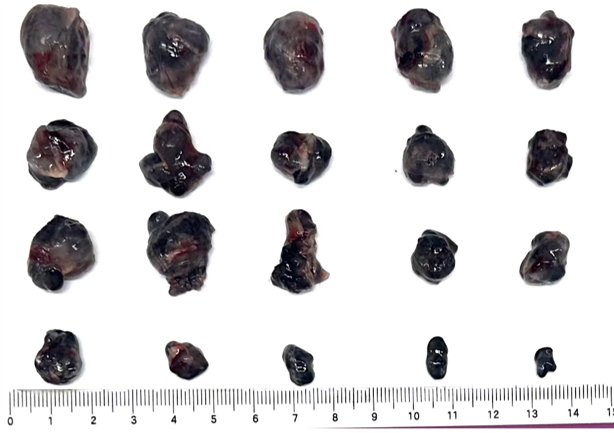

Supplement: Supplementary file 4 — Source data Fig. 1 [file 44321_2026_429_MOESM4_ESM.zip › Figure 1/1O/1T.png]

## Slide 1
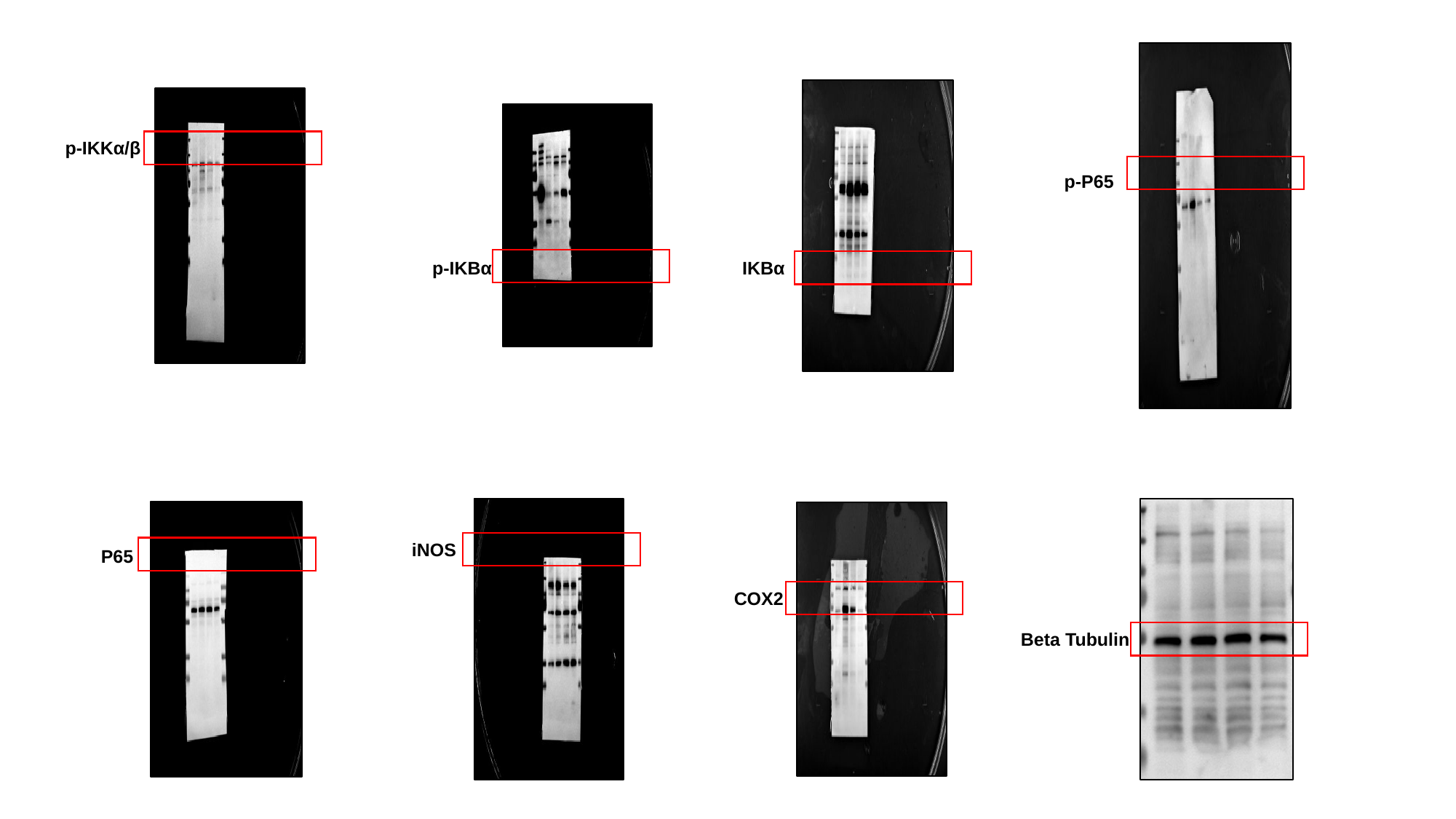

p-IKKα/β
p-P65
p-IKBα
IKBα
iNOS
P65
COX2
Beta Tubulin

Supplement: Supplementary file 6 — Source data Fig. 3 [file 44321_2026_429_MOESM6_ESM.zip › Figure 3/3E/3E.pptx]
