# Supplementary material for: Betrixaban activates cGAS-STING to promote antitumor immunity without pathological inflammation
Source: EMBO Mol Med. 2026 May 14;18(6):2213–35. doi: 10.1038/s44321-026-00429-1 (PMC13269763; doi:10.1038/s44321-026-00429-1)
Supplement: Supplementary file 8 — Expanded View Figures [file 44321_2026_429_MOESM8_ESM.pdf]

Expanded View Figures

Figure EV1. The antitumor ability of Betrixaban.

(**A**) Individual tumor growth curves for each mouse, corresponds to Fig. 1A. (**B**) Wild-type (WT) mice were inoculated with the indicated numbers of cGAS$^{-/-}$ Pan02 cells subcutaneously. Tumorigenesis was monitored every other day for 18 days. Representative images of tumors, tumor sizes, and tumor weights in PBS-treated control (Con) and BT-treated group. Unpaired *t* test ($n = 4$). (**C**) cGAS knockout mice were inoculated with the indicated numbers of Pan02 cells subcutaneously. Then treated with PBS or BT every two days. Representative images of tumors, tumor sizes, and tumor weights in PBS-treated control (Con) and BT-treated group. Unpaired *t* test ($n = 4$). (**D**) Representative FACS data and quantification of tumor-infiltrating CD45$^+$ CD8$^+$ TILs, IFNγ$^+$ TILs, TNFα$^+$ TILs of mice as in (**B**). Unpaired *t* test ($n = 3$). (**E**) Representative FACS data and quantification of tumor-infiltrating CD45$^+$ CD8$^+$ TILs, IFNγ$^+$ TILs, TNFα$^+$ TILs of mice as in (**C**). Unpaired *t* test ($n = 3$). (**F**) Individual tumor growth curves for each mouse, corresponds to Fig. 1L. (**G**) Tumor sizes of subcutaneous Pan02 implanted in cGAS knockout mice treated with the isotype antibody (200 μg/mouse i.p.), BT (50 mg/kg i.p.), anti-PD-1 antibody (200 μg/mouse i.p.), or BT plus anti-PD-1 antibody (Combo, combined treatment with BT and anti-PD-1 antibody). Tumorigenesis was monitored every other day for 18 days. Representative images of tumors, tumor sizes, and tumor weights. Ordinary one-way ANOVA test ($n = 4$). (**H**) Individual tumor growth curves for each mouse, corresponds to Fig. 1O. (**I**) KPC-luc cells were implanted orthotopically into the pancreas of C57BL/6 mice to establish an orthotopic KPC-luc pancreatic tumor model. Tumor burden was longitudinally monitored using bioluminescent imaging. Representative bioluminescent images as well as tumor photographs, tumor volume, and tumor weight were shown. (**J**) BT upregulates *Isg15* and *Rsad2* mRNA in KPC-Luc cells. (**K**) Representative images of the B16F10 experimental lung metastasis model and lung weights. (**L**) Representative CD8 immunofluorescence images and H&E staining images from (**K**). Data are shown as mean ± SEM.

© The Author(s)

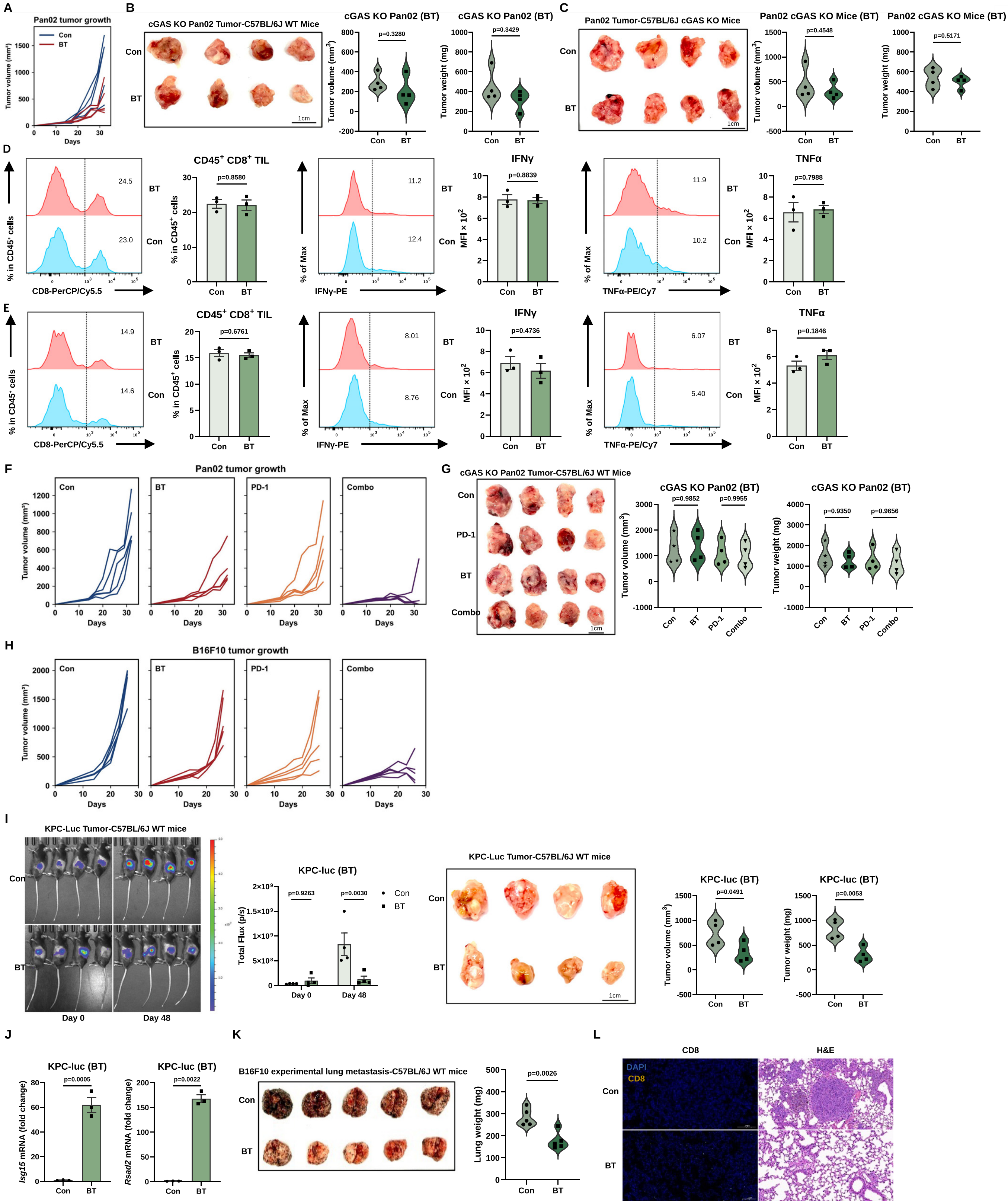
A
Pan02 tumor growth
B
cGAS KO Pan02 Tumor-C57BL/6J WT Mice
cGAS KO Pan02 (BT)
C
Pan02 Tumor-C57BL/6J cGAS KO Mice
Pan02 cGAS KO Mice (BT)
D
CD45+ CD8+ TIL
IFNγ
TNFα
E
F
Pan02 tumor growth
G
cGAS KO Pan02 Tumor-C57BL/6J WT Mice
H
B16F10 tumor growth
I
KPC-Luc Tumor-C57BL/6J WT mice
KPC-luc (BT)
J
K
B16F10 experimental lung metastasis-C57BL/6J WT mice
L
CD8
H&E

© The Author(s)

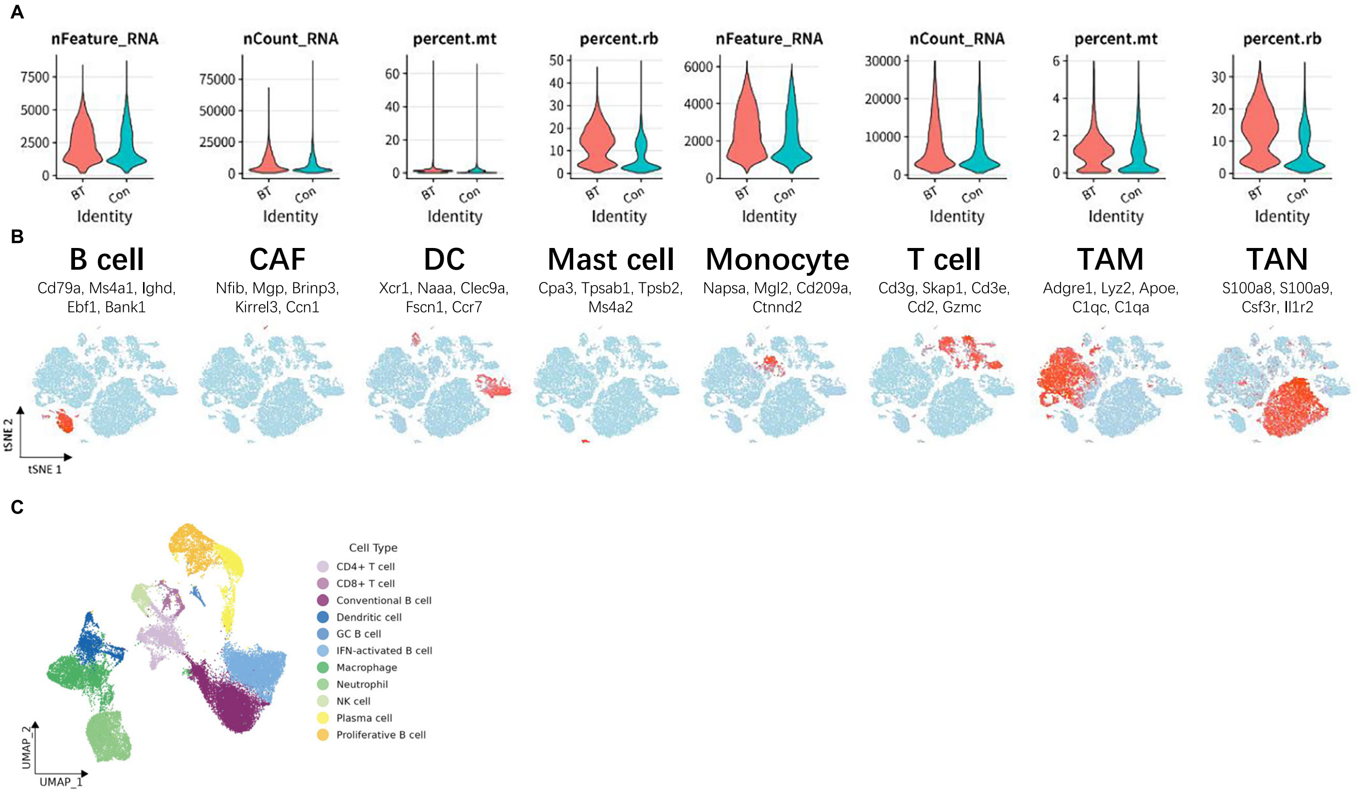

Figure EV2. Betrixaban enhances CD8⁺ TIL effector programs via transcriptional and epigenetic remodeling.

(**A**) The panel showed pre-QC distributions of key quality metrics and the corresponding post-QC distributions following application of our filtering criteria. (**B**) Key marker gene expression across cell clusters. (**C**) UMAP plot illustrating cell population clustering and annotation in $CD45^{+}$ Pan02 tumor samples treated with PBS (Con) and BT, with clusters color-coded by cell type.

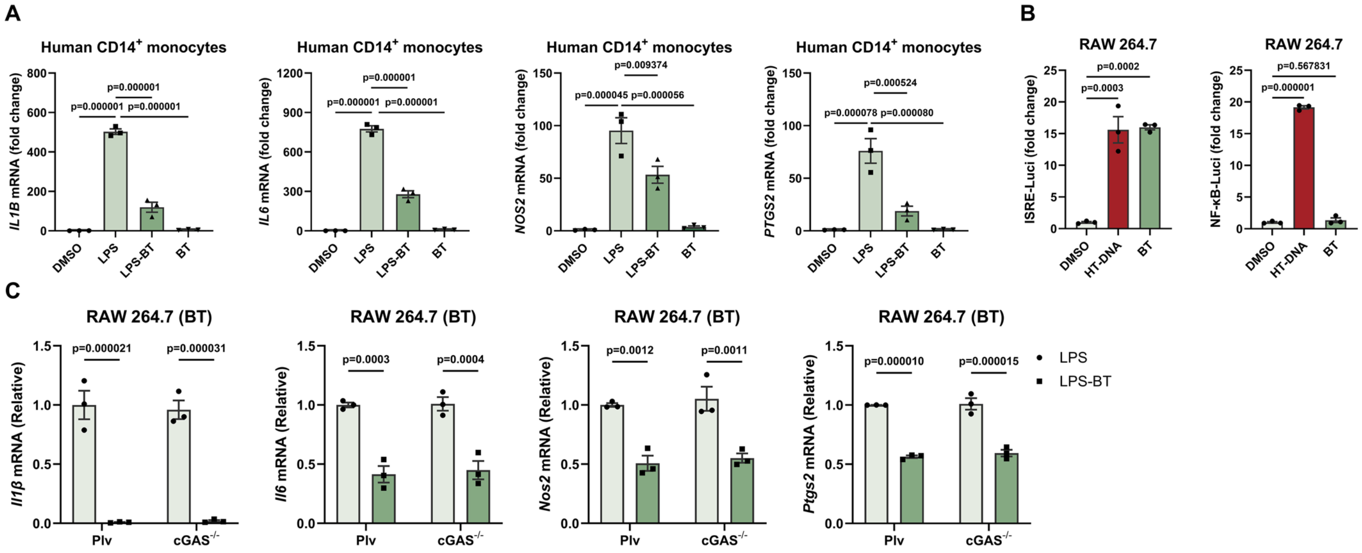

Figure EV3. Betrixaban suppresses LPS-induced inflammation.

(**A**) BT markedly downregulated the expression of *IL1B*, *IL6*, *NOS2*, *PTGS2* mRNA in human $CD14^{+}$ monocyte cells, following treatment with DMSO, LPS, LPS-BT, and BT alone. Ordinary one-way ANOVA test ($n = 3$). (**B**) Activation of the ISRE and NF-κB reporter systems was assessed by luciferase assay following treatment with HT-DNA and BT. Ordinary one-way ANOVA test ($n = 3$). (**C**) Expression of *Il1β*, *Il-6*, *Nos2*, and *Ptgs2* mRNA in either plv-treated or $cGAS^{-/-}$ RAW264.7 cells, following LPS priming and subsequent BT treatment. Two-way ANOVA test ($n = 3$). Data are shown as mean ± SEM.

 © The Author(s)

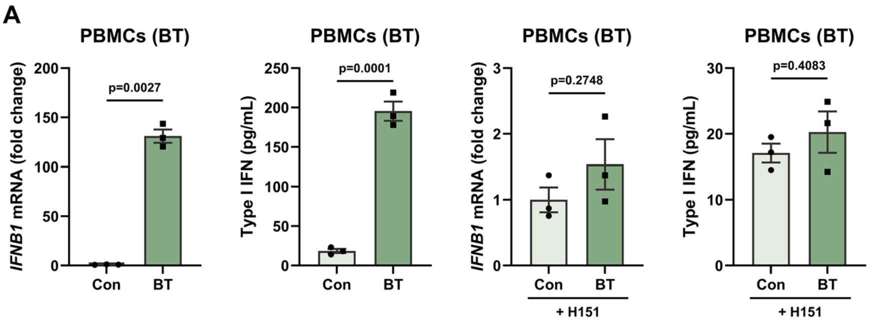

Figure EV4. Betrixaban directly activates cGAS via a noncanonical mechanism.

(**A**) PBMCs were assessed by RT-qPCR for *IFNB1* mRNA expression and by ELISA for Type I IFN production following BT or DMSO (Con) treatment, with or without H151 pretreatment. Unpaired *t* test ($n = 3$). Data are shown as mean ± SEM.

© The Author(s)
