# Supplementary material for: Betrixaban activates cGAS-STING to promote antitumor immunity without pathological inflammation
Source: EMBO Mol Med. 2026 May 14;18(6):2213–35. doi: 10.1038/s44321-026-00429-1 (PMC13269763; doi:10.1038/s44321-026-00429-1)
Supplement: Supplementary file 3 — Peer Review File [file 44321_2026_429_MOESM3_ESM.pdf]

EMBO Molecular Medicine

Betrixaban Activates cGAS-STING to Promote Antitumor Immunity without Pathological Inflammation

Yang Zhao, Xingyu Chen, LiRui Tang, Hanjie Liu, Boming Kang, Shuailong Zheng, Songying Ouyang, Yunfei Xie, and Fuping You

Corresponding authors: Yang Zhao (2311210031@stu.pku.edu.cn) , Songying Ouyang (ouyangsy@fjnu.edu.cn), Fuping You (fupingyou@hsc.pku.edu.cn), Yunfei Xie (qhx18@tsinghua.org.cn)

Review Timeline:

Submission Date:	12th Nov 25
Editorial Decision:	4th Dec 25
Revision Received:	23rd Feb 26
Editorial Decision:	23rd Mar 26
Revision Received:	1st Apr 26
Accepted:	7th Apr 26

Editor: Zeljko Durdevic

Transaction Report:

(Note: With the exception of the correction of typographical or spelling errors that could be a source of ambiguity, letters and reports are not edited. Depending on transfer agreements, referee reports obtained elsewhere may or may not be included in this compilation. Referee reports are anonymous unless the Referee chooses to sign their reports.)

4th Dec 2025

Dear Dr. Zhao,

Thank you for the submission of your manuscript to EMBO Molecular Medicine. We have now received feedback from the three reviewers who agreed to evaluate your manuscript. All three referees recognize interest of the study but also raise important concerns that should be addressed in a major revision. If you would like to discuss further the points raised by the referees, I am available to do so via email or video. Let me know if you are interested in this option.

Further consideration of a revision that addresses reviewers' concerns in full will entail a second round of review. EMBO Molecular Medicine encourages a single round of revision only and therefore, acceptance or rejection of the manuscript will depend on the completeness of your responses included in the next, final version of the manuscript. For this reason, and to save you from any frustrations in the end, I would strongly advise against returning an incomplete revision. Further, when submitting the revised manuscript please be sure to add institutional email addresses for Yang Zha and Yunfei Xie in the manuscript and our submission system

We would welcome the submission of a revised version within three months for further consideration. Please let us know if you require longer to complete the revision.

I look forward to receiving your revised manuscript.

Yours sincerely,

Zeljko Durdevic

Zeljko Durdevic
Senior Editor
EMBO Molecular Medicine

*****
When preparing your revised manuscript, please refer to our guidelines: https://link.springer.com/journal/44321/submission-guidelines#cms-Revised-submissions. We perform an initial quality control of all revised manuscripts before re-review; failure to include requested items will delay the evaluation of your revision.

We require:

1) A .docx formatted version of the manuscript text (including legends for main figures, EV figures and tables). Please make sure that the changes are highlighted to be clearly visible.

2) Individual production quality figure files as .eps, .tif, .jpg (one file per figure). For guidance, download the 'Figure Guide PDF': https://media.springernature.com/original/springer-cms/rest/v1/content/27825798/data/v1.

3) A .docx formatted letter INCLUDING the reviewers' reports and your detailed point-by-point responses to their comments. As part of the EMBO Press transparent editorial process, the point-by-point response is part of the Review Process File (RPF), which will be published alongside your paper.

4) A complete author checklist, which you can download from our author guidelines. Please insert information in the checklist that is also reflected in the manuscript. The completed author checklist will also be part of the RPF.

5) Please note that all corresponding authors are required to supply an ORCID ID for their name upon submission of a revised manuscript.

6) It is mandatory to include a 'Data Availability' section after the Materials and Methods. Before submitting your revision, primary datasets produced in this study need to be deposited in an appropriate public database, and the accession numbers and database listed under 'Data Availability'. Please remember to provide a reviewer password if the datasets are not yet public.

In case you have no data that requires deposition in a public database, please state so in this section. Note that the Data Availability Section is restricted to new primary data that are part of this study.

7) For data quantification: please specify the name of the statistical test used to generate error bars and P values, the number (n) of independent experiments (specify technical or biological replicates) underlying each data point and the test used to calculate p-values in each figure legend. The figure legends should contain a basic description of n, P and the test applied. Graphs must include a description of the bars and the error bars (s.d., s.e.m.).

8) At EMBO Press we ask authors to provide source data for the main manuscript figures. You will receive a separate email with instructions for providing source data with your revised manuscript, including how to upload and organize the files.

9) Our journal encourages inclusion of *data citations in the reference list* to directly cite datasets that were re-used and obtained from public databases. Data citations in the article text are distinct from normal bibliographical citations and should directly link to the database records from which the data can be accessed. In the main text, data citations are formatted as follows: "Data ref: Smith et al, 2001" or "Data ref: NCBI Sequence Read Archive PRJNA342805, 2017". In the Reference list, data citations must be labeled with "[DATASET]". A data reference must provide the database name, accession number/identifiers and a resolvable link to the landing page from which the data can be accessed at the end of the reference.

10) We replaced Supplementary Information with Expanded View (EV) Figures and Tables that are collapsible/expandable online. A maximum of 5 EV Figures can be typeset. EV Figures should be cited as 'Figure EV1, Figure EV2" etc... in the text and their respective legends should be included in the main text after the legends of regular figures.

- For the figures that you do NOT wish to display as Expanded View figures, they should be bundled together with their legends in a single PDF file called *Appendix*, which should start with a short Table of Content. Appendix figures should be referred to in the main text as: "Appendix Figure S1, Appendix Figure S2" etc.

- Additional Tables/Datasets should be labeled and referred to as Table EV1, Dataset EV1, etc. Legends have to be provided in a separate tab in case of .xls files. Alternatively, the legend can be supplied as a separate text file (README) and zipped together with the Table/Dataset file.

11) The paper explained: EMBO Molecular Medicine articles are accompanied by a summary of the articles to emphasize the major findings in the paper and their medical implications for the non-specialist reader. Please provide a draft summary of your article highlighting

- the medical issue you are addressing,

- the results obtained and

- their clinical impact.

This may be edited to ensure that readers understand the significance and context of the research. Please refer to any of our published articles for an example.

12) Author contributions: You will be asked to provide CRediT (Contributor Role Taxonomy) terms in the submission system. These replace a narrative author contribution section in the manuscript.

13) A Conflict of Interest statement should be provided in the main text.

14) Every published paper includes a 'Synopsis' to further enhance discoverability. Synopses are displayed on the journal webpage and are freely accessible to all readers. They include a short stand first (maximum of 300 characters, including space) as well as 2-5 one-sentences bullet points that summarizes the paper. Please write the bullet points to summarize the key NEW findings. They should be designed to be complementary to the abstract - i.e. not repeat the same text. We encourage inclusion of key acronyms and quantitative information (maximum of 30 words / bullet point). Please use the passive voice. Please attach these in a separate file or send them by email, we will incorporate them accordingly.

Please also suggest a striking image or visual abstract to illustrate your article as a PNG file 550 px wide x 300-600 px high.

15) Include a Reagents and Tools Table as part of the Methods section, which can be downloaded from our author guidelines.

EMBO Molecular Medicine has a "scooping protection" policy, whereby similar findings that are published by others during review or revision are not a criterion for rejection. Should you decide to submit a revised version, I do ask that you get in touch after three months if you have not completed it, to update us on the status.

***** Reviewer's comments *****

Referee #1 (Comments on Novelty/Model System for Author):

This study has an interesting idea, showing that Betrixaban may improve antitumor immune responses. The concept is good and worth exploring. However, a few important points need to be addressed:
Tumor models:
The paper only uses subcutaneous tumor models. These models do not fully represent real tumor environments. It would be better to add at least one orthotopic or more natural tumor model.
Human cells:
All key experiments were done in mouse cells. Because mouse and human cGAS-STING responses can be different, the authors should include some basic tests in human primary immune cells.
Betrixaban dosing and safety:
The dose of Betrixaban used in mice appears much higher than the dose used in humans. The authors should provide a clear rationale for this dosing and include data to show that such a high dose is safe in mice (for example, body weight, bleeding signs, or basic blood tests).
LPS model:
The study uses a very high-dose LPS model, which is not similar to typical human inflammatory situations. The authors should explain why this dose was chosen and whether it reflects real clinical immune reactions.
Single-cell analysis:
The single-cell RNA-seq uses t-SNE only. Since UMAP is now more commonly used and recommended, it would be helpful to include UMAP results to support the clustering.

Referee #2 (Comments on Novelty/Model System for Author):

The technical quality can be improved as detailed in the remarks to the authors. The desscribed DNA-independent activation of the cGAS-STING pathway is novel and interesting. There is no direct medical impact but translational value to further explore the effect of Betrixaban on cancer immunity (and probably sepsis-management). The models empoloyed are adequate.

Referee #2 (Remarks for Author):

The present manuscript: "Betrixaban Activates cGAS-STING to Promote Antitumour Immunity without Pathological Inflammation" by Zhao et al. describes Betrixaban-induced anti-tumor immunity. This was shown by reduced tumor growth, increased $CD8^+$ T cell infiltration, and improved efficacy of PD-1 immune checkpoint inhibition (ICI). In parallel, Betrixaban was shown to suppress the production of proinflammatory cytokines and to improve survival in lipopolysaccharide (LPS)-induced sepsis in mice. Mechanistically, the authors showed that Betrixaban activates cGAS in vitro in a DNA-independent manner.

The manuscript is well written and logically structured.

Main:
The authors claim that Betrixaban activated cGAS independently of DNA yet show increased activation in the presence of the latter. This should be further explored. Moreover, this crucial novel mechanism deserves further validation with alternative methodologies such as using cGAS-/- tumours for in vivo studies and cytosolic DNase expression in vitro.

Western blot raw data (uncut full membranes) need to be annexed.

Minor:
Error bars on tumour growth curves would further allow assessment of variation. Single curves should be annexed.

Numerical p-values should be favored over p-value ranges indicated by asterisks.

Single data points should be depicted for all graphs (including violin plots).

The size marker for tumors seems to be (copy-pasted) between Figure 1A and 1L.

Referee #3 (Comments on Novelty/Model System for Author):

This study investigates the dual role of the approved anticoagulant drug Betrixaban (BT) in cancer immunotherapy and inflammation control. The authors discovered that BT activates the cGAS-STING pathway via a non-canonical, DNA-independent mechanism, thereby promoting CD8 T cell-mediated antitumor immunity in tumor models, and exhibiting synergistic effects with PD-1 blockade. BT also effectively suppressed inflammatory responses in an LPS-induced sepsis model. Overall, it is an interesting study with translational application.

Referee #3 (Remarks for Author):

General Comments:
This study investigates the dual role of the approved anticoagulant drug Betrixaban (BT) in cancer immunotherapy and inflammation control. The authors discovered that BT activates the cGAS-STING pathway via a non-canonical, DNA-independent mechanism, thereby promoting CD8 T cell-mediated antitumor immunity in tumor models, and exhibiting synergistic effects with PD-1 blockade. BT also effectively suppressed inflammatory responses in an LPS-induced sepsis model. Overall, it is an interesting study with translational application. To strengthen the manuscript, the following points need to be addressed or clarified.

Major Points:
1.The authors claim that BT "elicits a robust IRF3-driven type I interferon response but limits the activation of the NF-κB-mediated inflammatory cascade". It is necessary to clarify how BT regulates these pathways, and compare to the signaling pathways regulated by canonical cGAS agonists.

2.To demonstrate that the antitumor efficacy of BT is dependent on the cGAS-STING axis, The key therapeutic experiments (e.g., BT monotherapy or in combination with anti-PD-1) must be performed in immunocompetent cGAS-KO mice .

3.Generating a cGAS mutant ablating BT binding could provide direct evidence of BT-induced IFN responses via cGAS.

4.Keep consistent format for referencing supplementary figures throughout the manuscript, either "Fig. S1" or "Supplementary Fig. 1"

Response to Reviewer 1

Referee #1 (Comments on Novelty/Model System for Author):

This study has an interesting idea, showing that Betrixaban may improve antitumor immune responses. The concept is good and worth exploring. However, a few important points need to be addressed:

1. Tumor models: The paper only uses subcutaneous tumor models. These models do not fully represent real tumor environments. It would be better to add at least one orthotopic or more natural tumor model.

Response: We thank the reviewer for this valuable comment and fully agree that subcutaneous tumors have limitations in recapitulating the native tumor microenvironment and metastatic disease. In response, we have added two additional in vivo models to the revised manuscript to evaluate the antitumor effects of BT in more physiological settings.

First, we established an orthotopic KPC-luc pancreatic tumor model by implanting KPC-luc cells into the pancreas of C57BL/6 mice. Tumor burden was monitored longitudinally by bioluminescence imaging. BT treatment markedly reduced orthotopic tumor growth compared with vehicle, as shown by significantly lower bioluminescent signal and decreased tumor weight at endpoint (Fig. EV1I). These results demonstrate that BT exerts robust antitumor activity in a clinically relevant pancreatic cancer model with an intact stromal and immune microenvironment.

Second, to assess the effect of BT on metastatic disease, we employed a B16F10 experimental lung metastasis model via tail-vein injection. BT-treated mice developed significantly fewer pulmonary metastatic nodules and reduced lung weight than vehicle-treated controls (Fig. EV1K), indicating that BT also suppresses hematogenous metastasis in an aggressive melanoma model.

Together with our original subcutaneous tumor experiments, these new orthotopic and metastatic models show that BT's antitumor efficacy is not restricted to ectopic flank tumors but extends to more natural tumor environments and metastatic settings. We believe these additional data address the reviewer's concern regarding the tumor models used in this study.

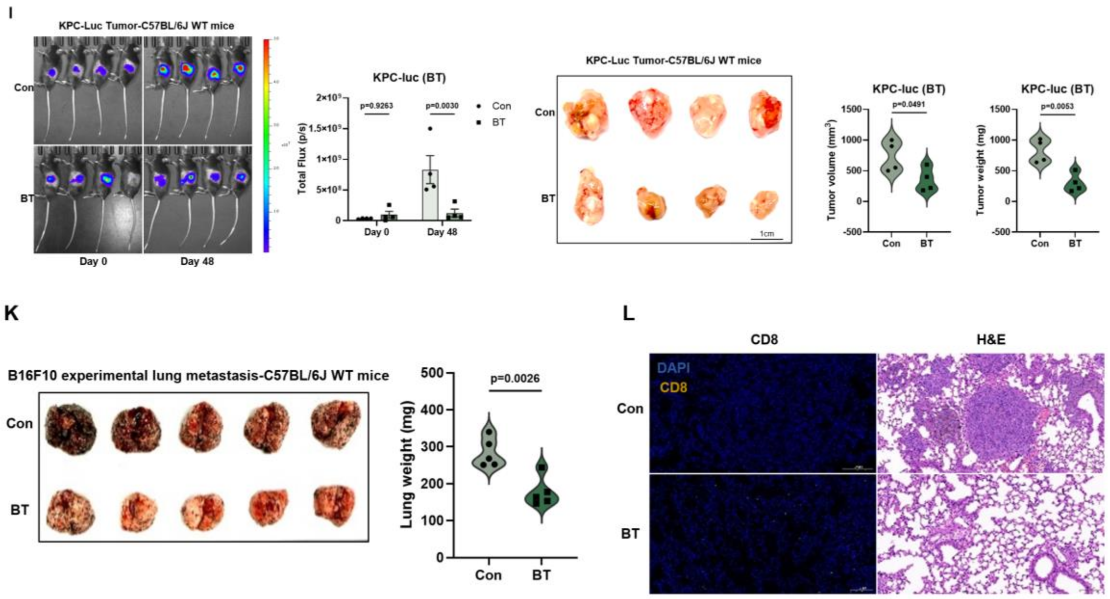

(1) Revised Sentence in Result: To evaluate BT in a more physiological pancreatic tumor setting, we established an orthotopic KPC-luc model. BT treatment markedly reduced orthotopic tumor burden, as shown by lower bioluminescent signals and decreased pancreatic tumor weight compared with vehicle (Fig. EV1I). Consistent with activation of an interferon response, qPCR analysis of orthotopic tumors showed significant upregulation of the interferon-stimulated genes Isg15 and Rsad2 in BT-treated mice (Fig. EV1J). To assess the effect of BT on metastatic disease, we further used a B16F10 experimental lung metastasis model via tail-vein injection. Compared with vehicle-treated mice, BT-treated animals developed markedly fewer pulmonary metastatic nodules and had reduced lung weight at endpoint (Fig. EV1K). To further validate the observed phenotype at the histological level, we performed immunofluorescence staining for CD8 and hematoxylin and eosin (H&E) staining on lung tissue sections. CD8 immunofluorescence revealed increased infiltration of $CD8^+$ T cells in the lungs of BT-treated mice, suggesting that the reduction in metastatic burden was accompanied by enhanced antitumor immune responses. H&E staining showed that control lungs were extensively infiltrated by tumor cells, with thickened alveolar septa and disrupted lung architecture. In contrast, BT-treated lungs exhibited smaller metastatic foci and markedly preserved lung structure (Fig. EV1L). Taken together, these results demonstrated that BT exerted a clear anti-metastatic effect in the B16F10 lung metastasis model, which was accompanied by enhanced $CD8^+$ T cell-associated immune features.

Together, these results demonstrated that BT effectively potentiated PD-1 blockade by enhancing both innate immune activation and adaptive T cell function, and exerted robust antitumor activity

across subcutaneous, orthotopic and metastatic tumor models. (Line 148 to 168)

(2) Revised Sentence in Material and Methods:

Orthotopic KPC-luc pancreatic tumor model

Orthotopic pancreatic tumors were established using luciferase-expressing KPC (KPC-luc) cells in immunocompetent mice. KPC-luc cells were maintained in DMEM supplemented with 10% FBS, 1% penicillin-streptomycin and 2 mM L-glutamine at 37°C in a humidified 5% CO_2 incubator. For orthotopic implantation, cells were harvested at 70-80% confluence, washed twice with sterile PBS, and resuspended in ice-cold PBS/Matrigel (1:1, v/v) at a final concentration of 2×10^7 cells/mL. Mice were anesthetized with isoflurane (3% for induction, 2% for maintenance in oxygen) and placed on a heating pad. After shaving and disinfecting the left flank, a 1-1.5 cm left subcostal incision was made to expose the spleen and underlying pancreatic tail.

Using a 29G insulin syringe, 2×10^5 KPC-luc cells in 50 μL PBS/Matrigel were slowly injected into the pancreatic tail. Successful implantation was confirmed by the formation of a visible bleb at the injection site without leakage into the peritoneal cavity. Mice in which obvious leakage occurred were excluded from the study. The pancreas and spleen were gently returned to the peritoneal cavity, the peritoneum and muscle were closed with absorbable sutures, and the skin was closed with wound clips or non-absorbable sutures. Mice received perioperative analgesia and were monitored daily for recovery, body weight, and signs of distress.

After implantation, tumor engraftment was verified by bioluminescence imaging, and mice with comparable baseline signals were randomized into treatment groups. BT was administered at 50 mg/kg by intraperitoneal injection once every other day. Control mice received PBS on the same schedule. At the experimental endpoint, mice were euthanized, and the pancreas with attached orthotopic tumor was dissected and weighed as a measure of tumor burden. Tumor tissue was snap-frozen for RNA extraction and RT-qPCR analysis.

Bioluminescence imaging of orthotopic KPC-luc tumors

Tumor burden in the orthotopic KPC-luc model was monitored longitudinally by in vivo bioluminescence imaging (BLI). Mice were injected intraperitoneally with D-luciferin (150 mg/kg in PBS) and anesthetized with isoflurane. Ten minutes after D-luciferin administration, images were acquired using an IVIS Spectrum imaging system with standardized acquisition parameters applied to all groups within each experiment.

Bioluminescent signals were analyzed using Living Image software. A fixed region of interest (ROI) was placed over the pancreatic area for each mouse, and total photon flux (photons/sec) within the ROI was quantified. For longitudinal analysis, total flux values at subsequent time points were normalized to the baseline signal for each mouse. These measurements were used as a surrogate for orthotopic tumor burden and for comparing tumor growth kinetics between vehicle- and BT-treated groups.

B16F10 experimental lung metastasis model

B16F10 cells were cultured in DMEM supplemented with 10% FBS. For tail-vein injection, subconfluent cultures were detached, washed with PBS, and resuspended in PBS at a final concentration of 1×10^5 cells/100 μL. Cells were slowly injected into the lateral tail vein using a 29G insulin syringe.

Mice were monitored daily for body weight, general condition, and signs of distress. BT was administered at 50 mg/kg by intraperitoneal injection once every other day, control mice received vehicle on the same schedule. At the experimental endpoint, mice were euthanized and the lungs were weighed as a measure of metastatic burden. Visible black metastatic nodules on the lung surface were counted under a dissecting microscope in a blinded manner.

Immunofluorescence Staining of CD8$^+$ T Cells

Paraffin-embedded lung sections were deparaffinized and rehydrated through a graded alcohol series. Antigen retrieval was conducted in citrate buffer (pH 6.0) using a microwave oven. Sections were blocked in 5% BSA with 0.3% Triton X-100 for 1 hour at room temperature, followed by overnight incubation at 4°C with anti-mouse CD8α primary antibody. After washing, slides were incubated with Alexa Fluor 488-conjugated secondary antibody for 1 hour at room temperature. Nuclei were counterstained with DAPI (1 μg/mL), and sections were mounted with antifade reagent. Fluorescent images were captured using a Nikon confocal microscope.

Hematoxylin-eosin (H&E) staining

The tissues were quickly placed in cold saline solution and rinsed after they were collected, then fixed in 4% paraformaldehyde, dehydrated, and embedded in paraffin prior to sectioning at 5 mm, and sections were stained with hematoxylin and eosin. (Line 570 to 625)

(3) Revised Sentence in Figure Legends:

(I) KPC-luc cells were implanted orthotopically into the pancreas of C57BL/6 mice to establish an

orthotopic KPC-luc pancreatic tumor model. Tumor burden was longitudinally monitored using bioluminescent imaging. Representative bioluminescent images as well as tumor photographs, tumor volume, and tumor weight were shown. (J) BT upregulates Isg15 and Rsad2 mRNA in KPC-Luc cells. (K) Representative images of the B16F10 experimental lung metastasis model and lung weights. (L) Representative CD8 immunofluorescence images and H&E staining images from Fig. EV1K. (Line 838 to 844)

2. Human cells: All key experiments were done in mouse cells. Because mouse and human cGAS-STING responses can be different, the authors should include some basic tests in human primary immune cells.

Response: We thank the reviewer for this important comment and fully agree that it is essential to assess BT activity in human primary immune cells. In the revised manuscript, we therefore performed additional experiments using primary human cells from healthy donors.

In response to the reviewer's concern about human systems, we added two sets of experiments in primary human immune cells. **First**, in PBMCs from healthy donors, BT stimulation induced robust *IFNB1* mRNA expression and IFN-β secretion, whereas pre-treatment with the cGAS inhibitor H-151 largely abolished these responses, indicating that BT-mediated IFN-I activation in human cells was cGAS-dependent. **Second**, in primary human CD14^{+} monocytes, BT co-treatment significantly attenuated LPS-induced upregulation of *IL6*, *IL1B*, *NOS2*, and *PTGS2* transcripts, demonstrating that BT also suppressed excessive inflammatory responses in human cells.

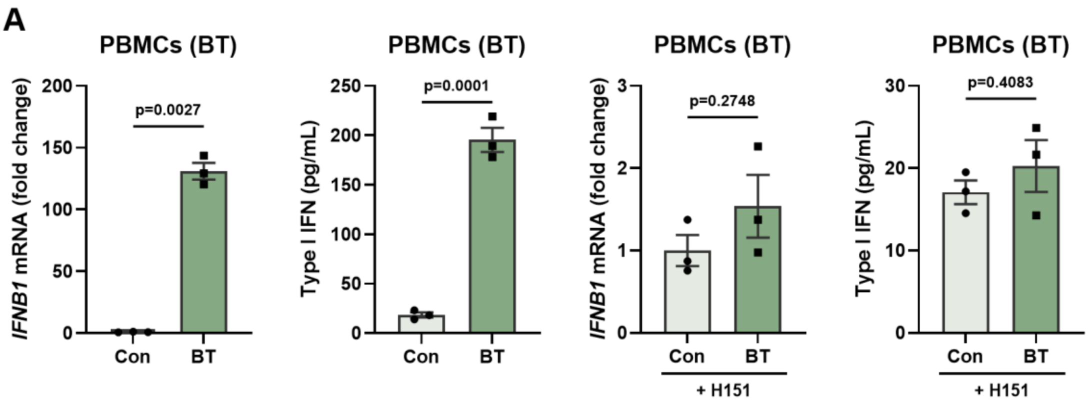

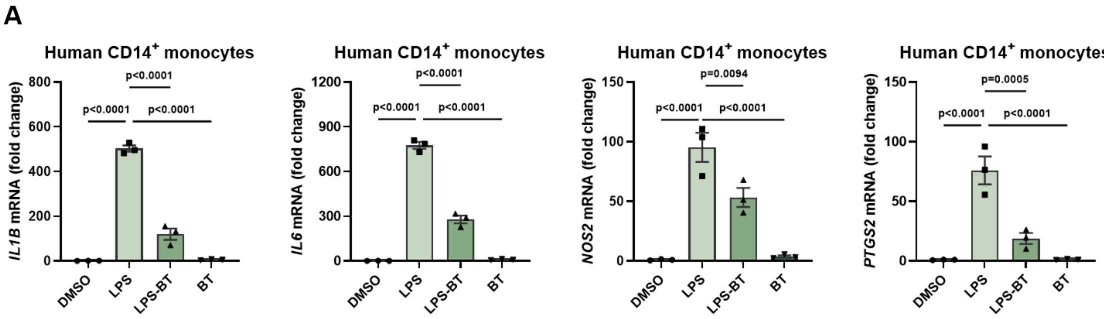

(1) Revised Sentence in Result: To assess whether BT also engaged the cGAS-STING pathway in primary human immune cells, we examined peripheral blood mononuclear cells (PBMCs) from healthy donors. BT stimulation significantly increased IFNB1 mRNA expression in PBMCs and elevated IFN-β secretion into the culture supernatant. Pre-treatment with the cGAS inhibitor H-151 markedly attenuated these BT-induced responses, reducing both IFNB1 transcript levels and IFN-β production to near-baseline values (Fig. EV4A). These findings indicated that BT activated the type I interferon program in primary human immune cells in a cGAS-dependent manner, providing a cellular correlate to its direct biochemical activation of human cGAS. (Line 324 to 331)

(2) Revised Sentence in Result: Consistent with these findings, BT co-treatment similarly reduced LPS-induced IL6, IL1B, NOS2, and PTGS2 mRNA levels in human CD14^{+} monocytes, with minimal impact on baseline expression (Fig. EV3A). (Line 238 to 240)

(3) Revised Sentence in Material and Methods: Human peripheral blood mononuclear cells (PBMCs) were bought from Shanghai Yayu Biotechnology Co., Ltd (YAYUBIO, Shanghai, China). CD14+ monocytes were isolated from PBMCs by magnetic bead-based positive selection (Selleck) according to the manufacturer's instructions and were used immediately for stimulation assays. Collection of peripheral blood from healthy adult donors and subsequent use of PBMCs were approved by the Institutional Review Board of Shanghai Liquan Hospital (protocol number 2022-09). Peripheral blood was obtained from three healthy adult Chinese donors (two males, 21 and 31 years old, and one female, 25 years old). (Line 490 to 497)

(4) Revised Sentence in Figure Legends:

(A) BT markedly downregulated the expression of *Il1β*, *Il-6*, *Nos2*, *Ptgs2* mRNA in human CD14^{+} monocyte cells, following treatment with DMSO, LPS, LPS-BT and BT alone. (Line 852 to 853)

(5) Revised Sentence in Figure Legends:

(A) PBMCs were assessed by RT-qPCR for IFNB1 mRNA expression and by ELISA for Type I IFN production following BT or DMSO (Con) treatment, with or without H151 pretreatment. (Line 858 to 859)

3. Betrixaban dosing and safety: The dose of Betrixaban used in mice appears much higher than the dose used in humans. The authors should provide a clear rationale for this dosing and include data to show that such a high dose is safe in mice (for example, body weight, bleeding signs, or basic blood tests).

Response: We thank the reviewer for this thoughtful comment. We agree that the rationale and safety of the Betrixaban (BT) dosing regimen in mice should be clearly explained. The dose used here was chosen based on our previous pharmacology work, in which this regimen produced robust Type I IFN signaling pathway activation and antitumor efficacy without overt toxicity in mice. Regarding safety, we have already performed a dedicated evaluation of this BT dosing regimen in mice in a separate study, which has been accepted by EMBO MOLECULAR MEDICIE for publication and is currently in proof stage (Chen *et al*, 2025).

In that work, we demonstrated daily administration of BT for 14 days caused no observable toxicity, as assessed by organ appearance, organ weights, body wight, and histological analysis of major tissues. These data indicate that the BT dose used in our mouse experiments is well tolerated and does not cause apparent toxicity. To avoid unnecessary duplication of published material, we have not re-plotted all of these safety data in the current manuscript, but we cite this study in the current manuscript. Meanwhile, we provided these data to reviewer in this response letter, and we are happy to provide the full safety in-press manuscript for reference if necessary.

We hope that this explanation and the previously established safety profile address the reviewer's concerns regarding BT dosing and safety in mice.

Revised Sentence in Material and Methods: The BT dosing regimen used in this study was previously reported to be well tolerated in mice without significant changes (Chen *et al.*, 2025). (Line 534 to 535)

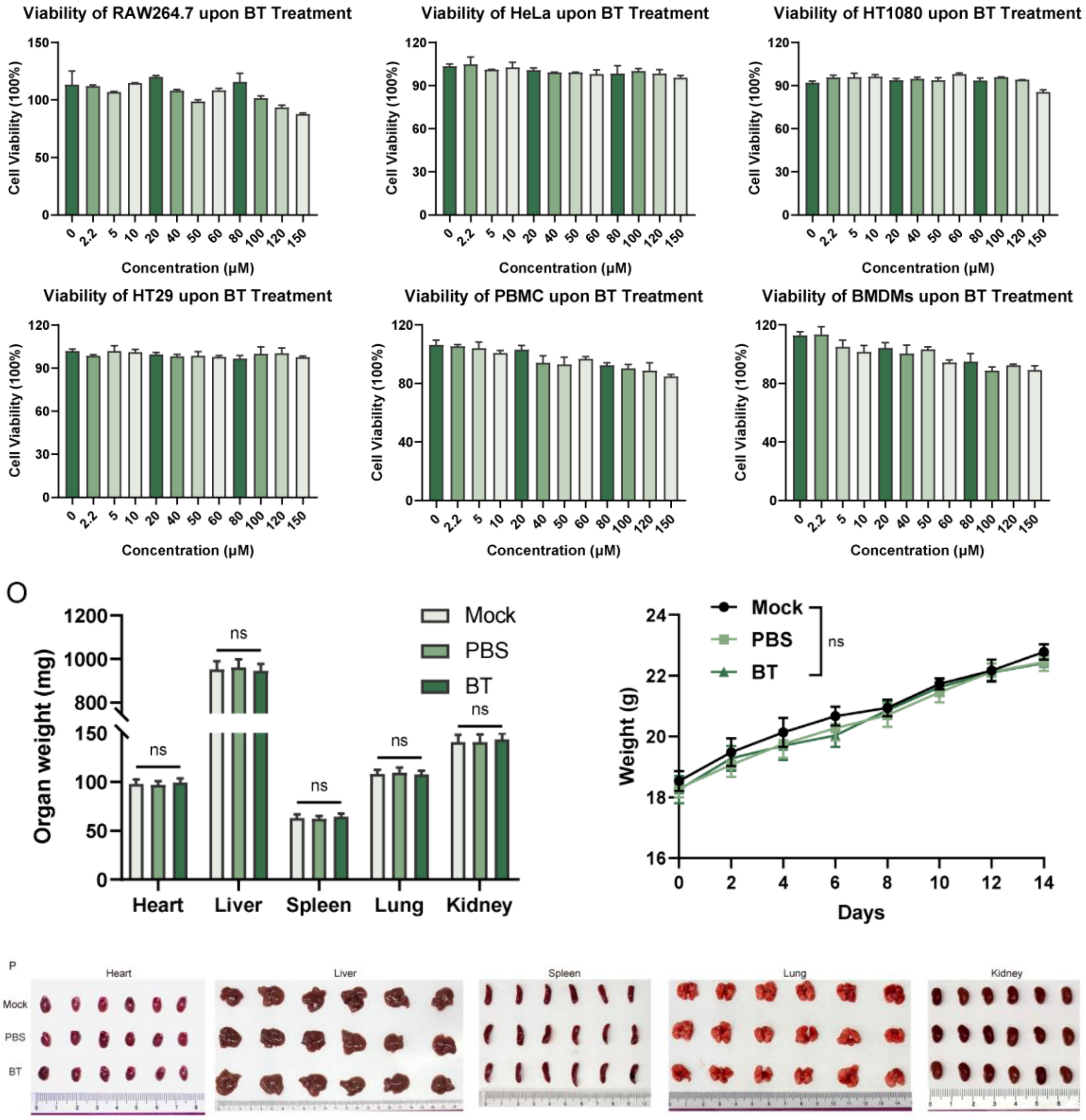
Viability of RAW264.7 upon BT Treatment
Viability of HeLa upon BT Treatment
Viability of HT1080 upon BT Treatment
Viability of HT29 upon BT Treatment
Viability of PBMC upon BT Treatment
Viability of BMDMs upon BT Treatment
Cell Viability (100%)
Concentration (μM)
0 2.2 5 10 20 40 50 60 80 100 120 150
O
Organ weight (mg)
Mock
PBS
BT
ns
Heart Liver Spleen Lung Kidney
Weight (g)
Days
P
Heart
Liver
Spleen
Lung
Kidney

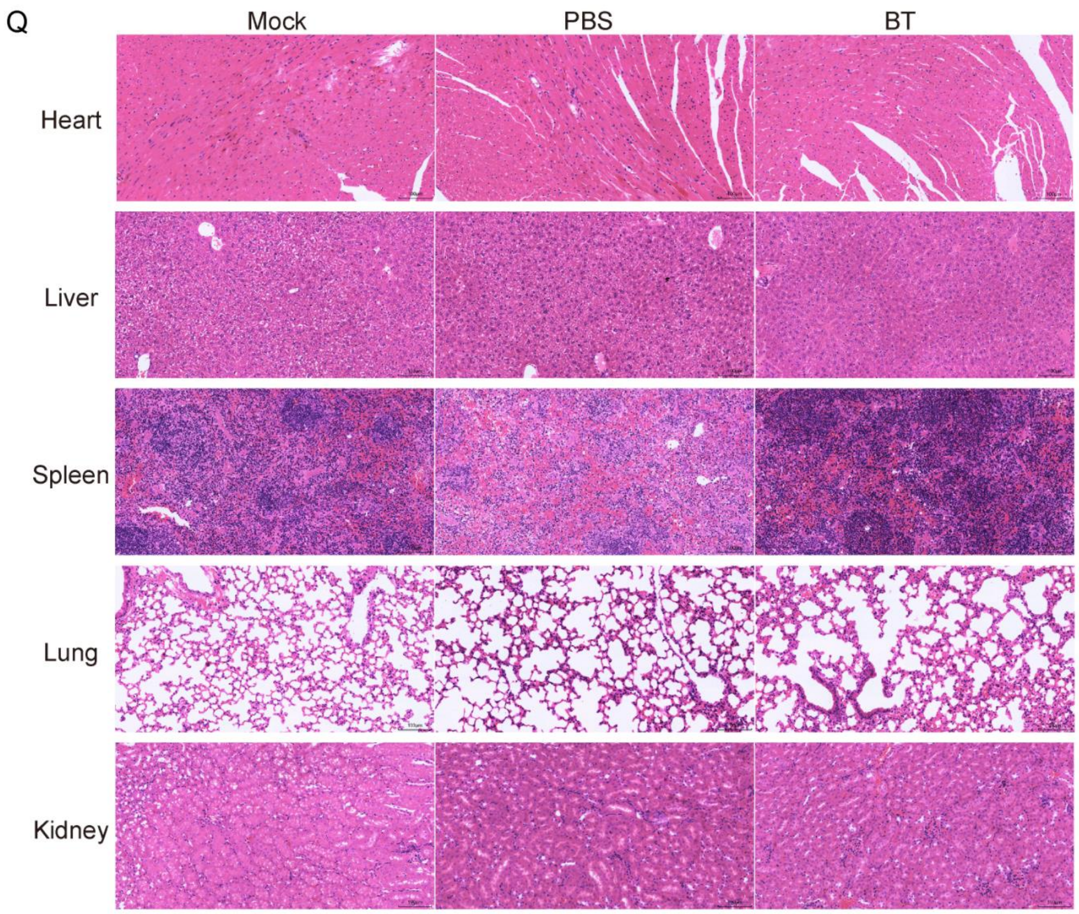

4. LPS model: The study uses a very high-dose LPS model, which is not similar to typical human inflammatory situations. The authors should explain why this dose was chosen and whether it reflects real clinical immune reactions.

Response: We appreciate this important comment and agree that the context of the LPS model needs to be clearly defined. In our study, we deliberately used a high dose of LPS (20 mg/kg, i.p.) as a lethal sepsis model, rather than as a model of mild or physiological inflammation. Mice are relatively insensitive to LPS, and similar high doses are widely used to reproducibly induce systemic inflammatory response syndrome, cytokine storm, multi-organ inflammation, and rapid mortality, thereby modelling the extreme hyperinflammatory state of severe sepsis or cytokine storm rather than everyday inflammatory conditions. We have now clarified this rationale in the revised Materials and methods, explicitly referring to this as a high-dose LPS-induced lethal sepsis model.

To better relate our findings to more typical inflammatory settings, we also examined the effect of BT under standard in vitro LPS stimulation conditions. In RAW264.7 cells, LPS robustly

upregulated *Il6*, *Il1b*, *Nos2* and *Ptgs2*, whereas BT co-treatment significantly reduced this LPS-induced transcription with minimal effects on basal expression. Consistent with this, BT similarly attenuated LPS-induced *IL6*, *IL1B*, *NOS2* and *PTGS2* mRNA expression in primary human $CD14^+$ monocytes.

Together, the high-dose in vivo model showed that BT can ameliorate severe sepsis-like hyperinflammation, while the RAW264.7 and human $CD14^+$ cell experiments demonstrated that BT also suppressed LPS-driven inflammatory responses under standard, non-extreme conditions, thereby addressing the reviewer' s concerns about both the choice and the clinical relevance of the LPS model.

Revised Sentence in Material and Methods: We deliberately used a high dose of LPS (20 mg/kg, i.p.) as a lethal sepsis model, rather than as a model of mild or physiological inflammation. Mice are relatively insensitive to LPS, and similar high doses are widely used to reproducibly induce systemic inflammatory response syndrome, cytokine storm, multi-organ inflammation, and rapid mortality, thereby modelling the extreme hyperinflammatory state of severe sepsis or cytokine storm rather than everyday inflammatory conditions. (Line 529 to 534)

5. Single-cell analysis: The single-cell RNA-seq uses t-SNE only. Since UMAP is now more commonly used and recommended, it would be helpful to include UMAP results to support the clustering.

Response: We appreciate the reviewer' s suggestion and agree that UMAP is a useful complementary visualization method. In the revised manuscript, we have re-embedded our single-cell RNA-seq dataset using UMAP with the same set of highly variable genes and principal components as in the original t-SNE analysis. The UMAP projection recapitulated the overall structure observed with t-SNE. We have added this UMAP plot as Fig. EV2C and now state in the Results section that UMAP yielded clustering patterns consistent with t-SNE. We hope this addition adequately addresses the reviewer' s concern.

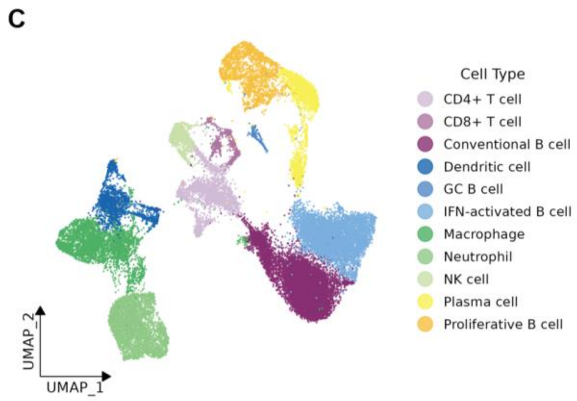

(1) Revised Sentence in Results: Consistent results were obtained when visualizing the same data with UMAP, which showed the same separation of major immune cell lineages (Fig. EV2C). (Line 180 to 182)

(2) Revised Sentence in Figure Legends:

(C) UMAP plot illustrating cell population clustering and annotation in $CD45^{+}$ Pan02 tumor samples treated with PBS (Con) and BT, with clusters color-coded by cell type. (Line 849 to 850)

Chen X, Zhao Y, Xie Y, Liu T, Wang H, Wang X, Guo X, You F (2025) Betrixaban Activates cGAS and ERVs to Promote Dual Nucleic-Sensing Antiviral Immunity. 2025.2008.2008.669242

Response to Reviewer 2

Referee #2 (Comments on Novelty/Model System for Author):

The technical quality can be improved as detailed in the remarks to the authors. The desscribed DNA-independent activation of the cGAS-STING pathway is novel and interesting. There is no direct medical impact but translational value to further explore the effect of Betrixaban on cancer immunity (and probably sepsis-management). The models empoloyed are adequate.

Referee #2 (Remarks for Author):

The present manuscript: "Betrixaban Activates cGAS-STING to Promote Antitumour Immunity without Pathological Inflammation" by Zhao et al. describes Betrixaban-induced anti-tumor immunity. This was shown by reduced tumor growth, increased $CD8^+$ T cell infiltration, and improved efficacy of PD-1 immune checkpoint inhibition (ICI). In parallel, Betrixaban was shown to suppress the production of proinflammatory cytokines and to improve survival in lipopolysaccharide (LPS)-induced sepsis in mice. Mechanistically, the authors showed that Betrixaban activates cGAS in vitro in a DNA-independent manner.

The manuscript is well written and logically structured.

Main:

1.The authors claim that Betrixaban activated cGAS independently of DNA yet show increased activation in the presence of the latter. This should be further explored.

Response: We thank the reviewer for pointing out this important conceptual issue. Our intention was not to imply that BT activates cGAS in a manner completely independent of DNA, but rather that BT can induce cGAMP production by purified cGAS in the absence of exogenous dsDNA and further enhances cGAS activity when dsDNA is present.

To clarify this, we have revised the wording throughout the Results and Discussion to describe BT as inducing a partially DNA-independent, noncanonical activation of cGAS that synergizes with

dsDNA instead of fully replacing it. In the revised manuscript, we now explicitly highlight that BT alone induces a modest but reproducible increase in cGAMP, whereas dsDNA produces a robust activation, and the combination of BT and dsDNA yields the highest cGAMP levels. This quantitative pattern supports the view that BT can activate cGAS in the absence of exogenous DNA, while further potentiating DNA-driven activation.

In addition, we now emphasize in the text that BT retains low but detectable activity on DNA-binding-interface and Zn^{2+}-thumb mutants that are essentially unresponsive to dsDNA, consistent with an allosteric, noncanonical mechanism that does not rely solely on the canonical DNA-binding interface. We hope that these clarifications address the reviewer's concern regarding how DNA and BT jointly contribute to cGAS activation.

(1) Revised Sentence in Results: This pattern indicated that BT did not replace DNA, but rather provided additional activation both in the absence and presence of dsDNA. (Line 316 to 317)

(2) Revised Sentence in Results: For the catalytic triple mutant, the response to BT was abolished (Fig. 4B-E). These results demonstrated that BT directly activated cGAS through a noncanonical mechanism that can support cGAMP production in the absence of exogenous dsDNA and does not rely solely on canonical DNA binding or Zn^{2+} coordination. (Line 319 to 323)

(3) Revised Sentence in Results: BT's activation likely involved a binding site distinct from the canonical DNA-binding interface, which may help explain its ability to induce type I interferon (IFN-I) while limiting the induction of pro-inflammatory cytokines. Compared with classical activation by cytosolic DNA, which triggers both antiviral and pro-inflammatory cytokines, BT appeared to bias the cGAS–STING pathway toward antiviral signaling with relatively restrained inflammatory responses in our models. This selective modulation suggests that the pathway can be pharmacologically tuned to elicit protective immunity without pathological inflammation, providing a potential strategy for immunotherapies with strong antitumor activity and reduced inflammatory toxicity. (Line 332 to 339)

(4) Revised Sentence in Discussion: Mechanistically, BT's ability to activate the cGAS-STING pathway even in the absence of exogenous dsDNA, while further enhancing DNA-driven activation, was a striking finding of this study. (Line 376 to 378)

(5) Revised Sentence in Discussion: In this study, we also observed BT-induced IFN-I responses in primary human PBMCs, but future work will be required to determine how BT modulates

diverse human immune cell subsets and tumor–immune interactions in more physiological settings. (Line 428 to 430)

2.Moreover, this crucial novel mechanism deserves further validation with alternative methodologies such as using cGAS$^{-/-}$ tumours for in vivo studies and cytosolic DNase expression in vitro.

Response: We thank the reviewer for emphasizing the need to rigorously validate the proposed cGAS-dependent mechanism in vivo. In response, we have now performed additional loss-of-function experiments combining both tumour-intrinsic and host-intrinsic cGAS deficiency.

First, we generated cGAS$^{-/-}$ Pan02 cells using CRISPR/Cas9 and implanted them subcutaneously into WT C57BL/6J mice. In this model, BT treatment failed to significantly inhibit tumor growth or reduce tumor weight in mice bearing cGAS$^{-/-}$ tumors (Fig. EV1B). Flow cytometry analysis further showed that BT failed to increase intratumoral CD8^{+} T cell infiltration or IFN-γ^{+}/TNF-α^{+} in cGAS-/- tumours (Fig. EV1D).

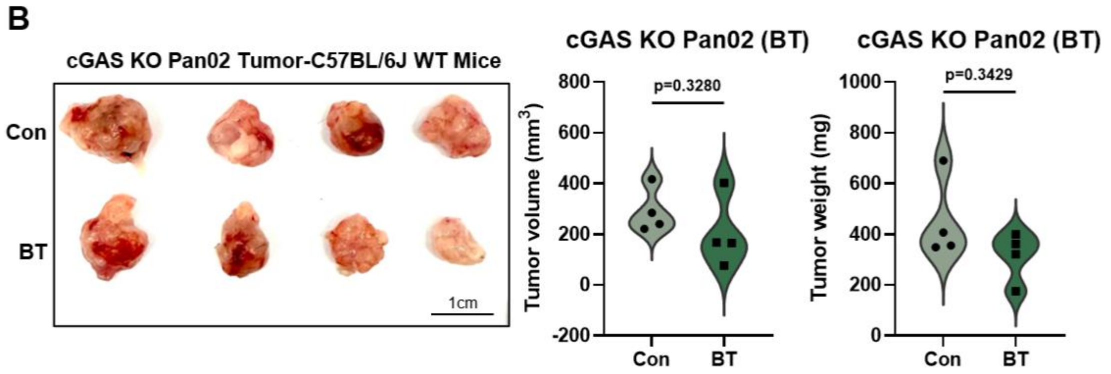

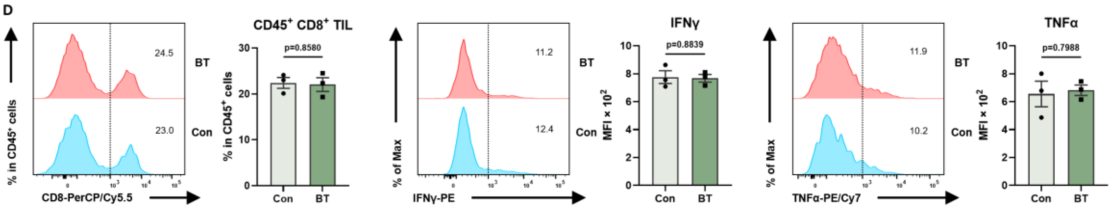

Second, to assess the contribution of host cGAS, we implanted WT Pan02 cells into cGAS$^{-/-}$ mice and treated with BT or vehicle. BT failed to suppress tumor growth in cGAS-/- hosts (Fig. EV1C), and BT did not enhance CD8^{+} T cell activation or IFN-γ^{+}/TNF-α^{+} cells in cGAS$^{-/-}$ mice (Fig. EV1E).

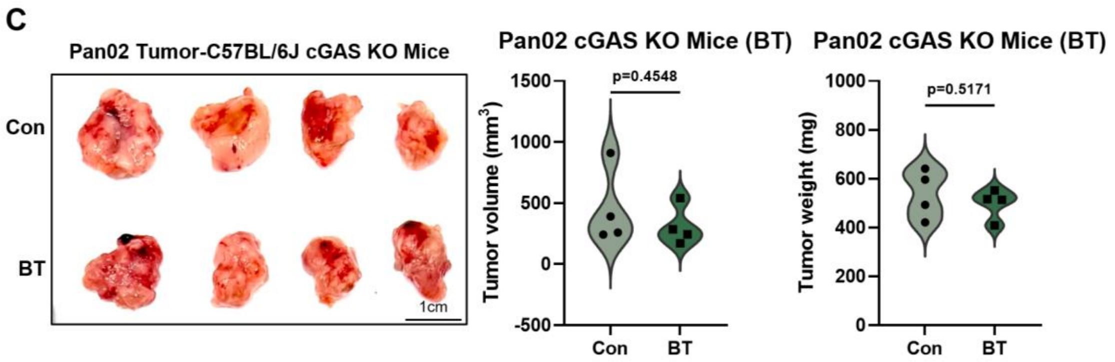

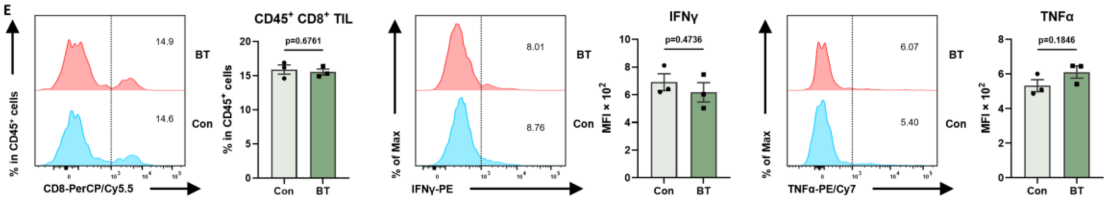

Third, we further evaluated BT in combination with anti-PD-1 in cGAS-deficient hosts. WT Pan02 cells were implanted into cGAS$^{-/-}$ mice, which were then randomized to receive vehicle, BT alone, anti-PD-1 alone, or BT plus anti-PD-1. In contrast to WT mice, where BT markedly potentiated the antitumor efficacy of anti-PD-1, in cGAS$^{-/-}$ mice BT monotherapy did not significantly inhibit tumour growth, and the BT plus anti-PD-1 combination did not provide additional benefit beyond anti-PD-1 alone (Fig. EV1G).

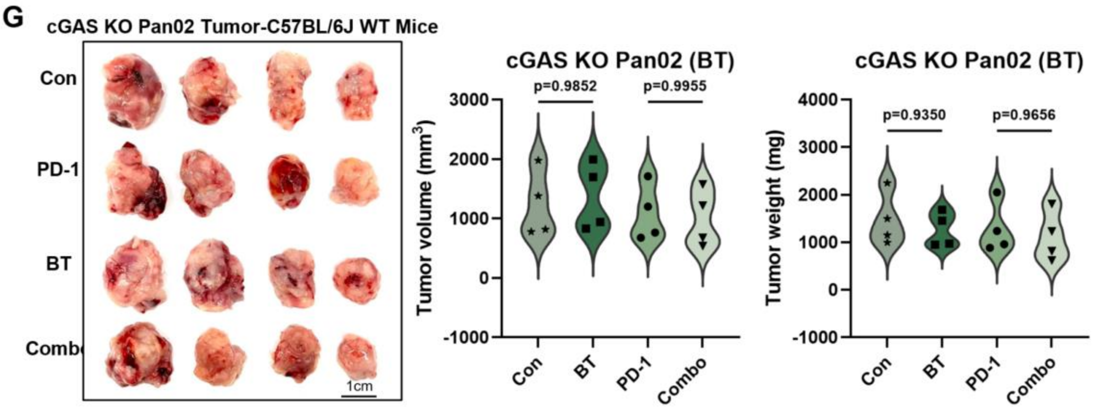

Together, these complementary models showed that both tumour cell-intrinsic and host immune cGAS were required for the full antitumor activity of BT in vivo.

In addition, we added an experiment in PBMCs from healthy donors. BT stimulation induced robust *IFNB1* mRNA expression and IFN-β secretion, whereas pre-treatment with the cGAS inhibitor H-151 largely abolished these responses, indicating that BT-mediated IFN-I activation in human cells was cGAS-dependent (Fig. EV4A). Meanwhile, our biochemical data already showed that BT directly activated purified human cGAS in the absence of exogenous DNA and further

enhanced DNA-driven cGAMP production (Fig. 4A).

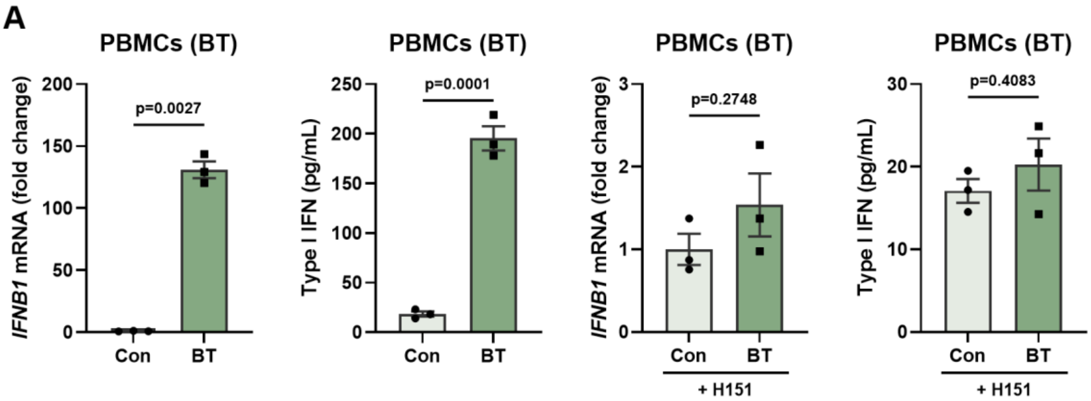

We fully agree that manipulating cytosolic DNases to dissect the contribution of endogenous DNA would provide further mechanistic insight. However, we believe that the current set of orthogonal approaches, including biochemical activation of purified cGAS, tumour and host cGAS loss-of-function in vivo, and cGAS-dependent IFN induction in human primary cells, already provides a robust validation of the proposed mechanism. A detailed dissection of how endogenous cytosolic DNA and DNases modulate BT-driven cGAS activation will be an important direction for future work.

(1) Revised Sentence in Results: To further validate the cGAS-dependent mechanism of BT, we used four experimental groups, including both cGAS-/- tumor cell and cGAS-/- host models. First, we implanted cGAS-/- Pan02 cells in WT mice and treated them with BT or PBS. Tumor images showed no significant reduction in tumor size compared to PBS-treated controls (Fig. EV1B). Similarly, in cGAS-/- mice bearing WT Pan02 tumors, BT also failed to reduce tumor growth. Consistently, tumor volume and tumor weight confirmed that BT treatment had no significant effect (Fig. EV1C). Flow cytometry analysis of tumors showed BT failed to increase infiltration of $CD8^+$ TILs or their IFN-γ/TNF-α production (Fig. EV1D-E). These results confirmed that both tumor and host cGAS were essential for the full antitumor effect of BT. (Line 119 to 127)

(2) Revised Sentence in Results: Consistently, BT failed to inhibit tumour growth in cGAS-/- mice, and the BT plus anti-PD-1 combination did not provide additional benefit beyond anti-PD-1 alone (Fig. EV1G). (Line 134 to 135)

(3) Revised Sentence in Result: To assess whether BT also engaged the cGAS-STING pathway in primary human immune cells, we examined peripheral blood mononuclear cells (PBMCs) from healthy donors. BT stimulation significantly increased *IFNB1* mRNA expression in PBMCs and

elevated IFN-β secretion into the culture supernatant. Pre-treatment with the cGAS inhibitor H-151 markedly attenuated these BT-induced responses, reducing both *IFNB1* transcript levels and IFN-β production to near-baseline values (Fig. EV4A). These findings indicated that BT activated the type I interferon program in primary human immune cells in a cGAS-dependent manner, providing a cellular correlate to its direct biochemical activation of human cGAS. (Line 324 to 331)

(4) Revised Sentence in Material and Methods: Human peripheral blood mononuclear cells (PBMCs) were bought from Shanghai Yayu Biotechnology Co., Ltd (YAYUBIO, Shanghai, China). CD14+ monocytes were isolated from PBMCs by magnetic bead-based positive selection (Selleck) according to the manufacturer's instructions and were used immediately for stimulation assays. Collection of peripheral blood from healthy adult donors and subsequent use of PBMCs were approved by the Institutional Review Board of Shanghai Liquan Hospital (protocol number 2022-09). Peripheral blood was obtained from three healthy adult Chinese donors (two males, 21 and 31 years old, and one female, 25 years old). (Line 490 to 497)

(5) Revised Sentence in Material and Methods:

CRISPR/Cas9 gene editing

The lentivirus-mediated CRISPR/Cas9 technology was used to generate cGAS knockout Pan02 cells. Briefly, the highly efficient and specific guide RNAs (sgRNAs) targeting the gene of interest were designed and selected by using the online CRISPR design website. The oligonucleotide pairs encoding sgRNAs were synthesized, annealed, and inserted into the lentiCRISPR v2 vector that had been digested by the BsmBI enzyme (NEB). For lentivirus production, the transfection mixture of constructed lentiCRISPR v2 vector (2400 ng), packaging plasmid psPAX2 (800 ng), envelope plasmid VSV-G (800 ng), and PEI (1600 ng) was added into 293T cells and incubated at 37°C for 72 h. The supernatant containing virus was then collected and added to Pan02 cells supplemented with polybrene (Beyotime Biotechnology, China). At 48 h post-infection, cells were cultured in fresh media containing 3 μg/mL puromycin (Sigma-Aldrich, USA) for stable selection of transduced cells. The successful knockout cell line was validated by Sanger sequencing. (Line 498 to 509)

(6) Revised Sentence in Figure Legends:

(B) Wild-type (WT) mice were inoculated with the indicated numbers of cGAS-/- Pan02 cells

subcutaneously. Tumorigenesis was monitored every other day for 18 days. Representative images of tumors, tumor sizes and tumor weights in PBS-treated control (Con) and BT-treated group. Unpaired t test (n = 4). (C) cGAS knockout mice were inoculated with the indicated numbers of Pan02 cells subcutaneously. Then treated with PBS or BT every two days. Representative images of tumors, tumor sizes and tumor weights in PBS-treated control (Con) and BT-treated group. Unpaired t test (n = 4). (D) Representative FACS data and quantification of tumor infiltrating CD45+ CD8+ TILs, IFNγ+ TILs, TNFα+ TILs of mice as in B. Unpaired t test (n = 3). (E) Representative FACS data and quantification of tumor infiltrating CD45+ CD8+ TILs, IFNγ+ TILs, TNFα+ TILs of mice as in C. Unpaired t test (n = 3). (Line 822 to 832)

(7) Revised Sentence in Figure Legends:

(G) Tumor sizes of subcutaneous Pan02 implanted in cGAS knockout mice treated with the isotype antibody (200 μg/mouse i.p.), BT (50 mg/kg i.p.), anti-PD-1 antibody (200 μg/mouse i.p.) or BT plus anti-PD-1 antibody (Combo, combined treatment with BT and anti-PD-1 antibody). Tumorigenesis was monitored every other day for 18 days. Representative images of tumors, tumor sizes and tumor weights. Ordinary one-way ANOVA test (n = 4). (Line 833 to 837)

3.Western blot raw data (uncut full membranes) need to be annexed.

Response: We thank the reviewer for this important suggestion and fully agree that providing the raw Western blot data is essential for transparency and reproducibility. In the revised submission, we have now included the uncropped, full-length membranes for all Western blots presented in the figures as source data files. The bands used in the main figures are indicated on the full membranes for clarity. In addition, we have provided densitometric quantification of the Western blot signals (normalized to loading controls) in the Appendix Fig. S1, which correspond to the bar graphs shown in the main text. We hope that these additions satisfactorily address the reviewer's request regarding Western blot raw data.

Minor:

4.Error bars on tumour growth curves would further allow assessment of variation. Single curves should be annexed.

Response: We thank the reviewer for this helpful recommendation. In the revised manuscript, we have replotted all tumor growth curves with error bars, thereby visualizing the variability across biological replicates. In addition, we now provide the individual tumour growth curves for each

mouse as separate plots in Fig. EV1A, EV1F and EV1H. We agree that these additions improve the transparency and assessment of variation in our in vivo experiments.

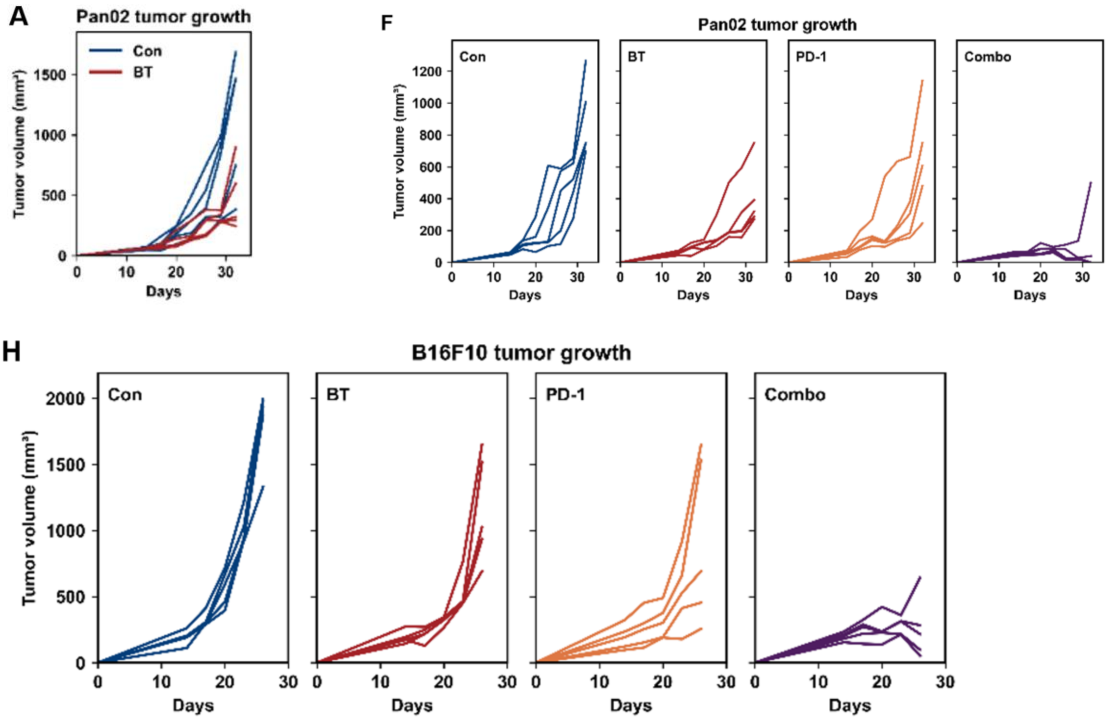

Revised Sentence in Figure Legends:

(A) Individual tumor growth curves for each mouse, corresponds to Fig. 1A. (Line 822)

(F) Individual tumor growth curves for each mouse, corresponds to Fig. 1L. (Line 832 to 833)

(H) Individual tumor growth curves for each mouse, corresponds to Fig. 1O. (Line 837 to 838)

5.Numerical p-values should be favored over p-value ranges indicated by asterisks.

Response: We appreciate the reviewer's suggestion. In the revised manuscript, we have replaced all asterisk-based significance annotations with the corresponding numerical p-values for every figure, including the main and supplementary panels. In addition, the figure legends have been updated to specify the statistical tests used. We believe this presentation improves the transparency and interpretability of our statistical analyses.

6.Single data points should be depicted for all graphs (including violin plots).

Response: We thank the reviewer for this helpful suggestion. In the revised manuscript, we have re-plotted all graphs so that individual data points for each biological replicate are now explicitly shown. Data points are overlaid on the summary statistics, and the figure legends have been updated accordingly. We believe this improves the transparency and interpretability of our results.

7.The size marker for tumors seems to be (copy-pasted) between Figure 1A and 1L.

Response: We appreciate the reviewer’s careful inspection of the tumor images. We have double-checked the original, uncropped image files and confirm that Figures 1A and 1L were obtained from independent experiments but were photographed on the same imaging board with a fixed ruler. Therefore, the appearance of the size marker is very similar between the two panels, although no copy-and-paste or digital manipulation was performed.

To avoid any misunderstanding, in the revised manuscript we have reassembled the tumor images: we removed the physical ruler from the panels and instead added a standardized digital scale bar, and we now provide the full, unedited original photographs as Source Data. We hope this clarification and the inclusion of the raw images address the reviewer’s concern regarding the size marker.

Response to Reviewer 3

Referee #3 (Comments on Novelty/Model System for Author):

This study investigates the dual role of the approved anticoagulant drug Betrixaban (BT) in cancer immunotherapy and inflammation control. The authors discovered that BT activates the cGAS-STING pathway via a non-canonical, DNA-independent mechanism, thereby promoting CD8 T cell-mediated antitumor immunity in tumor models, and exhibiting synergistic effects with PD-1 blockade. BT also effectively suppressed inflammatory responses in an LPS-induced sepsis model. Overall, it is an interesting study with translational application.

Referee #3 (Remarks for Author):

General Comments:

This study investigates the dual role of the approved anticoagulant drug Betrixaban (BT) in cancer immunotherapy and inflammation control. The authors discovered that BT activates the cGAS-STING pathway via a non-canonical, DNA-independent mechanism, thereby promoting CD8 T cell-mediated antitumor immunity in tumor models, and exhibiting synergistic effects with PD-1 blockade. BT also effectively suppressed inflammatory responses in an LPS-induced sepsis model. Overall, it is an interesting study with translational application. To strengthen the manuscript, the following points need to be addressed or clarified.

Major Points:

1.The authors claim that BT "elicits a robust IRF3-driven type I interferon response but limits the activation of the NF-κB-mediated inflammatory cascade". It is necessary to clarify how BT regulates these pathways, and compare to the signaling pathways regulated by canonical cGAS agonists.

Response: We sincerely thank the reviewer for this thoughtful and constructive comment. We agree that our previous description did not sufficiently explain how BT differentially impacts the IRF3/type I interferon branch and the NF-κB inflammatory branch, nor did it provide a direct

comparison with canonical cGAS agonists. To address this, we therefore performed luciferase reporter assays in stable RAW-ISRE and RAW-NF-κB cells using cytosolic dsDNA transfection as a canonical cGAS stimulus, and we additionally assessed LPS-induced inflammatory gene expression by RT-qPCR in cGAS-knockout RAW264.7 cells. We revised the manuscript accordingly to present a clearer mechanistic comparison.

First, to directly compare BT with a canonical cGAS stimulus, we performed luciferase reporter assays in stable RAW264.7 reporter cell lines, including RAW-ISRE (IRF3/type I IFN axis) and RAW-NF-κB (inflammatory axis). Cytosolic dsDNA transfection (HT-DNA) robustly activated both ISRE and NF-κB reporters, in line with canonical cGAS-STING signaling that can engage both the TBK1-IRF3 and IKK-NF-κB branches. In contrast, under matched conditions, BT induced strong ISRE reporter activity while eliciting little to no activation of the NF-κB reporter, indicating an IFN-dominant, inflammation-sparing signaling output (Fig. EV3B).

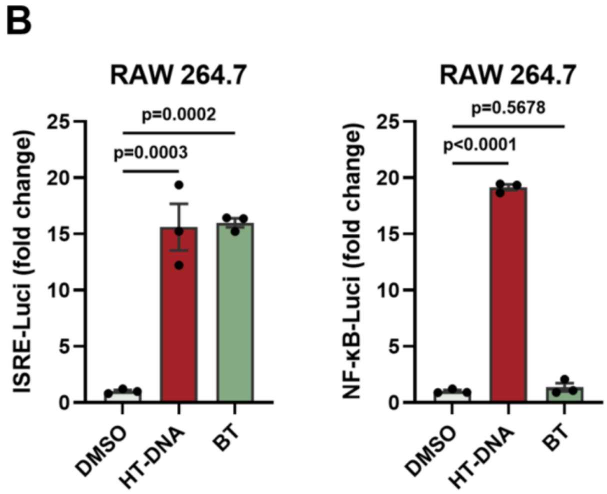

Second, to determine whether the anti-inflammatory effect of BT depended on cGAS, we generated cGAS-knockout RAW264.7 cells and examined inflammatory transcriptional outputs by RT-qPCR in an LPS-challenged setting. In wild-type RAW264.7 cells, BT markedly reduced LPS-induced expression of representative NF-κB-dependent inflammatory genes, including *Il1β*, *Il6*, *Nos2* and *Ptgs2*. Importantly, a comparable suppression was observed in cGAS-knockout RAW264.7 cells (Fig. EV3C), indicating that BT can dampen NF-κB–driven inflammatory gene induction independently of cGAS. These consistent with our chromatin accessibility and motif analyses showing reduced NF-κB/AP-1-associated regulatory activity upon BT treatment.We now explicitly describe this point in the revised manuscript.

C

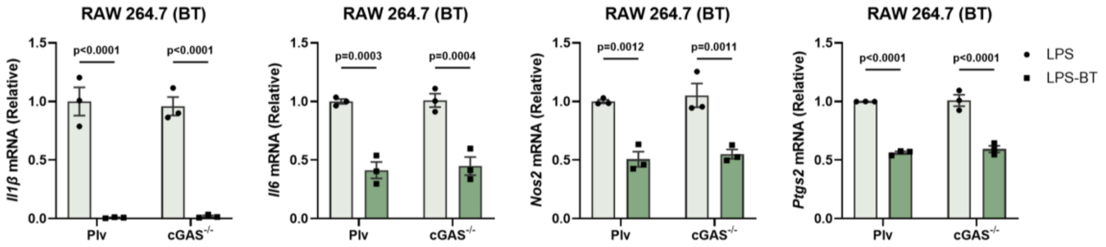

(1) Revised Sentence in Results: In line with these findings, stable RAW-ISRE and RAW-NF-κB reporter assays further showed that BT robustly induced ISRE activity while leaving NF-κB reporter activity near baseline, in contrast to cytosolic dsDNA (HT-DNA) which strongly activated both pathways (Fig. EV3B); importantly, BT also reduced LPS-induced inflammatory gene expression in cGAS-knockout RAW264.7 cells by RT-qPCR (Fig. EV3C). (Line 241 to 246)

(2) Revised Sentence in Figure Legends:

(B) Activation of the ISRE and NF-κB reporter systems was assessed by luciferase assay following treatment with HT-DNA and BT. (C) Expression of Il1β, Il-6, Nos2, and Ptgs2 mRNA in either plv-treated or cGAS-/- RAW264.7 cells, following LPS priming and subsequent BT treatment. (Line 853 to 856)

(3) Revised Sentence in Material and Methods:

Luciferase assay

Cells were seeded into 12-well plates and incubated overnight. Cells were then treated with BT for 12 h at 37°C in a humidified atmosphere with 5% CO2. After incubation, cells were lysed directly in the wells using lysis buffer provided in the luciferase assay kit (TransGen Biotech, cat. FR203-01), following the manufacturer's instructions. The lysates were collected by gentle pipetting, and luciferase activity was measured using a TD20/20 Luminometer (Turner Designs). (Line 632 to 637)

2.To demonstrate that the antitumor efficacy of BT is dependent on the cGAS-STING axis, The key therapeutic experiments (e.g., BT monotherapy or in combination with anti-PD-1) must be performed in immunocompetent cGAS-KO mice.

Response: We fully agree with the reviewer that the antitumor activity of BT should be rigorously tested in cGAS-deficient settings, particularly for the key therapeutic experiments. In the revised manuscript, we have therefore performed additional loss-of-function studies that address both tumour-intrinsic and host-intrinsic cGAS, and we have directly repeated the BT monotherapy and

BT plus anti-PD-1 combination experiments in cGAS$^{-/-}$ mice.

First, to examine the requirement for tumour cell-intrinsic cGAS, we generated cGAS$^{-/-}$ Pan02 cells using CRISPR/Cas9 and implanted them subcutaneously into WT C57BL/6J mice. In this model, BT treatment failed to significantly inhibit tumor growth or reduce tumor weight in mice bearing cGAS$^{-/-}$ tumors (Fig. EV1B). Consistently, flow cytometry analysis showed that BT failed to increase intratumoral $CD8^+$ T cell infiltration or IFN-γ^+/TNF-α^+ cells in cGAS$^{-/-}$ tumours (Fig. EV1D).

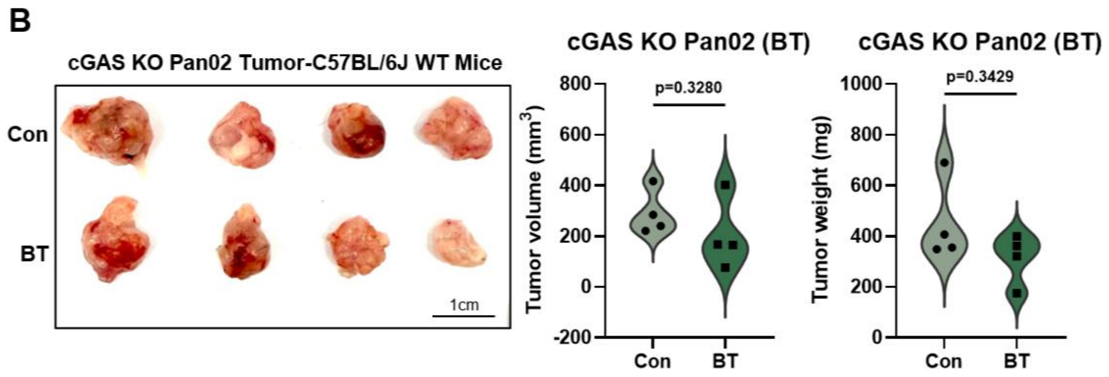

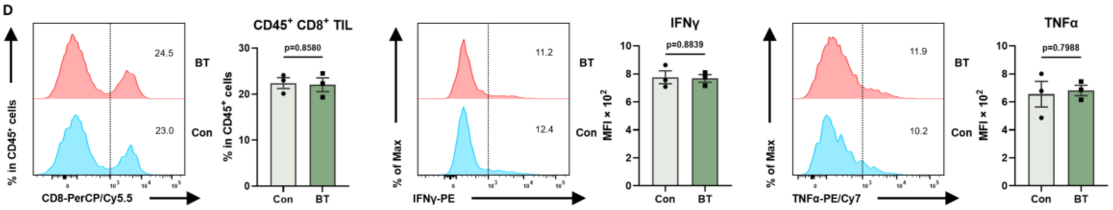

Second, to directly fulfill the reviewer's request for key therapeutic experiments in cGAS-KO mice, we implanted WT Pan02 cells into either cGAS$^{-/-}$ mice and treated them with BT or vehicle. BT failed to suppress tumor growth in cGAS-/- hosts (Fig. EV1C), and BT did not enhance $CD8^+$ T cell activation or IFN-γ^+/TNF-α^+ cells in cGAS$^{-/-}$ mice (Fig. EV1E). This demonstrated that cGAS was crucial to antitumor efficacy of BT.

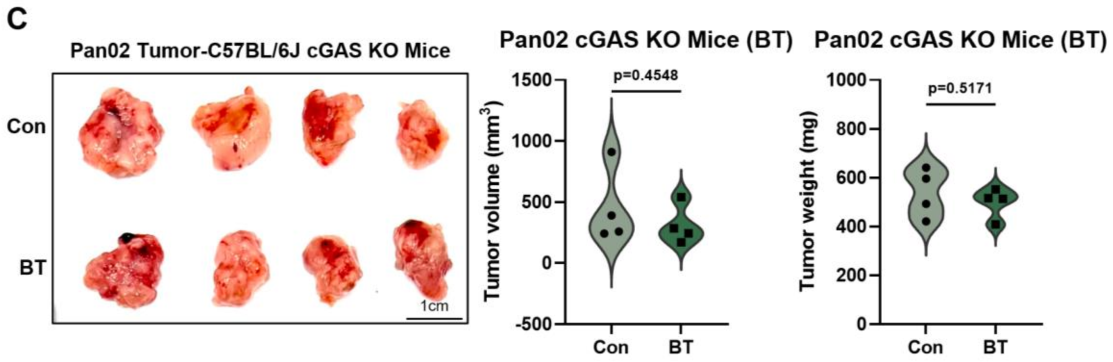

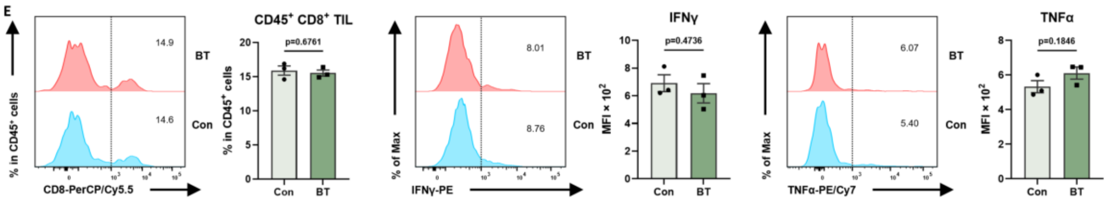

Third, because the reviewer specifically highlighted the combination with anti-PD-1 as a key therapeutic setting, we further evaluated BT plus anti-PD-1 in cGAS$^{-/-}$ hosts. WT Pan02 cells were implanted into cGAS$^{-/-}$ mice, which were then randomized to receive vehicle, BT alone, anti-PD-1 alone, or BT plus anti-PD-1. In contrast to WT mice, where BT markedly potentiated the antitumor efficacy of anti-PD-1, in cGAS$^{-/-}$ mice BT did not significantly inhibit tumour growth, and the BT plus anti–PD-1 combination did not provide additional benefit beyond anti-PD-1 alone (Fig. EV1G). These data indicated that the capacity of BT to enhance PD-1 checkpoint blockade also critically depended on an intact cGAS pathway in immunocompetent hosts.

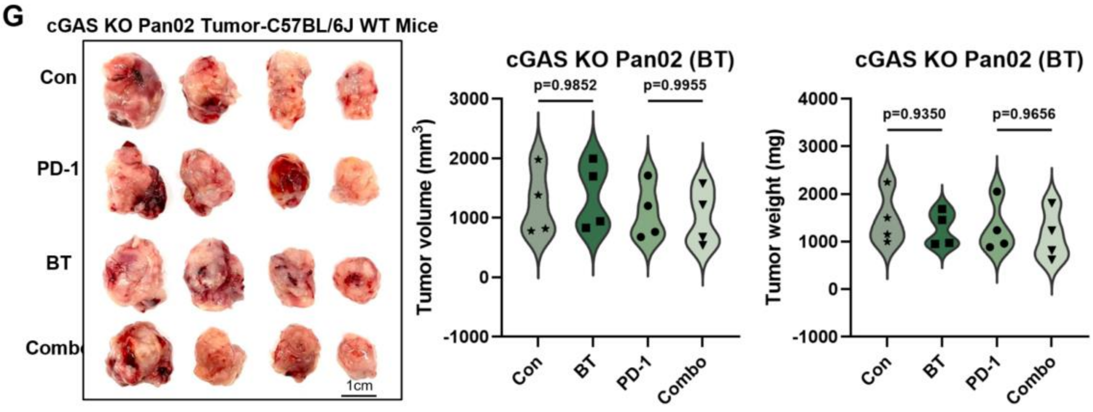

Together, these complementary models showed that both tumour cell-intrinsic cGAS and host immune cGAS are required for the full antitumor activity of BT in vivo, and they directly address the reviewer's request to perform the key therapeutic experiments in immunocompetent cGAS-KO mice.

(1) Revised Sentence in Results: To further validate the cGAS-dependent mechanism of BT, we used four experimental groups, including both cGAS-/- tumor cell and cGAS-/- host models. First, we implanted cGAS-/- Pan02 cells in WT mice and treated them with BT or PBS. Tumor images showed no significant reduction in tumor size compared to PBS-treated controls (Fig. EV1B). Similarly, in cGAS-/- mice bearing WT Pan02 tumors, BT also failed to reduce tumor growth. Consistently, tumor volume and tumor weight confirmed that BT treatment had no significant

effect (Fig. EV1C). Flow cytometry analysis of tumors showed BT failed to increase infiltration of $CD8^+$ TILs or their IFN-γ/TNF-α production (Fig. EV1D-E). These results confirmed that both tumor and host cGAS were essential for the full antitumor effect of BT. (Line 119 to 127)

(2) Revised Sentence in Results: Consistently, BT failed to inhibit tumour growth in cGAS-/- mice, and the BT plus anti-PD-1 combination did not provide additional benefit beyond anti-PD-1 alone (Fig. EV1G). (Line 134 to 135)

(3) Revised Sentence in Material and Methods:

CRISPR/Cas9 gene editing

The lentivirus-mediated CRISPR/Cas9 technology was used to generate cGAS knockout Pan02 cells. Briefly, the highly efficient and specific guide RNAs (sgRNAs) targeting the gene of interest were designed and selected by using the online CRISPR design website. The oligonucleotide pairs encoding sgRNAs were synthesized, annealed, and inserted into the lentiCRISPR v2 vector that had been digested by the BsmBI enzyme (NEB). For lentivirus production, the transfection mixture of constructed lentiCRISPR v2 vector (2400 ng), packaging plasmid psPAX2 (800 ng), envelope plasmid VSV-G (800 ng), and PEI (1600 ng) was added into 293T cells and incubated at 37℃ for 72 h. The supernatant containing virus was then collected and added to Pan02 cells supplemented with polybrene (Beyotime Biotechnology, China). At 48 h post-infection, cells were cultured in fresh media containing 3 μg/mL puromycin (Sigma-Aldrich, USA) for stable selection of transduced cells. The successful knockout cell line was validated by Sanger sequencing. (Line 498 to 509)

(4) Revised Sentence in Figure Legends:

(B) Wild-type (WT) mice were inoculated with the indicated numbers of cGAS-/- Pan02 cells subcutaneously. Tumorigenesis was monitored every other day for 18 days. Representative images of tumors, tumor sizes and tumor weights in PBS-treated control (Con) and BT-treated group. Unpaired t test (n = 4). (C) cGAS knockout mice were inoculated with the indicated numbers of Pan02 cells subcutaneously. Then treated with PBS or BT every two days. Representative images of tumors, tumor sizes and tumor weights in PBS-treated control (Con) and BT-treated group. Unpaired t test (n = 4). (D) Representative FACS data and quantification of tumor infiltrating CD45+ CD8+ TILs, IFNγ+ TILs, TNFα+ TILs of mice as in B. Unpaired t test (n = 3). (E) Representative FACS data and quantification of tumor infiltrating CD45+ CD8+ TILs, IFNγ+

TILs, TNFα+ TILs of mice as in C. Unpaired t test (n = 3). (Line 822 to 832)

(5) Revised Sentence in Figure Legends:

(G) Tumor sizes of subcutaneous Pan02 implanted in cGAS knockout mice treated with the isotype antibody (200 μg/mouse i.p.), BT (50 mg/kg i.p.), anti-PD-1 antibody (200 μg/mouse i.p.) or BT plus anti-PD-1 antibody (Combo, combined treatment with BT and anti-PD-1 antibody). Tumorigenesis was monitored every other day for 18 days. Representative images of tumors, tumor sizes and tumor weights. Ordinary one-way ANOVA test (n = 4). (Line 833 to 837)

3.Generating a cGAS mutant ablating BT binding could provide direct evidence of BT-induced IFN responses via cGAS.

Response: We fully agree with the reviewer that a cGAS mutant selectively abolishing BT binding while preserving DNA sensing would provide very direct evidence for BT-induced interferon responses via cGAS. Motivated by this suggestion, we performed additional exploratory structure-guided mutagenesis experiments.

Briefly, we carried out molecular docking of BT onto cGAS to predict a putative BT-contacting surface that is distinct from the canonical DNA-binding and catalytic interfaces. Based on these docking results, we introduced point mutations at several residues within the predicted pocket, expressed and purified the corresponding cGAS variants, and quantified 2′3′-cGAMP production by LC-MS in the presence of BT (± dsDNA) (data not shown). All of these mutants retained robust DNA-dependent enzymatic activity, and BT continued to enhance cGAMP synthesis to a similar extent as in WT cGAS. Thus, within the limits of this initial mutagenesis screen, we were not able to identify a single-point mutant that selectively abolished BT-induced activation while preserving normal DNA-driven cGAS function. These negative but informative results suggest that BT may engage a more distributed or allosteric binding surface on cGAS that is not easily disrupted by a small number of point mutations. We are also planning these structural investigations as a follow-up project.

In light of this, we have revised the manuscript text to more cautiously describe BT as a noncanonical activator of cGAS based on our current biochemical and genetic evidence. We also noted in discussion that high-resolution structural studies of cGAS in complex with BT is necessary. However, we believe that the existing data already provide multiple independent lines of evidence that BT-induced IFN responses are mediated by cGAS, including direct binding by

SPR, BT-stimulated cGAMP production in vitro, loss of BT responses in cGAS-deficient cells and primary human PBMCs treated with a cGAS inhibitor, and abrogation of BT's antitumor efficacy in both tumour-intrinsic and host-intrinsic cGAS-knockout models in vivo. We hope that these comprehensive biochemical and genetic data, together with the planned structural work, adequately address the reviewer's concern about the cGAS-dependent mechanism.

Revised Sentence in Discussion: Fourth, although our biochemical assays, cGAS-deficient cells, and tumour-intrinsic and host-intrinsic cGAS-knockout models collectively support a cGAS-dependent mechanism, the precise structural basis of BT-cGAS recognition remains unclear. Our current data indicated that BT functioned as a noncanonical activator of cGAS, but they did not yet pinpoint the exact binding interface at atomic resolution. High-resolution structural studies of cGAS in complex with BT, together with more extensive mutagenesis, will therefore be required to map the BT-binding surface and to fully elucidate how BT biases cGAS-STING signalling towards IFN-I-dominated responses with limited NF-κB activation. (Line 443 to 451)

4.Keep consistent format for referencing supplementary figures throughout the manuscript, either "Fig. S1" or "Supplementary Fig. 1"

Response: We appreciate the reviewer for drawing our attention to this formatting inconsistency. In the revised manuscript, we have carefully re-checked the entire text for all references to figures and have standardized the format. We believe these changes improve the clarity and internal consistency of the manuscript formatting, and we thank the reviewer again for this helpful suggestion.

23rd Mar 2026

Dear Dr. Zhao,

Thank you for submitting your revised manuscript to EMBO Molecular Medicine. Your manuscript will be ready for acceptance pending the following final amendments:

1) Please address referees' minor points.
2) Authors:
- We note that you currently have together with you, a total of 4 co-corresponding authors. Is that correct? Do you confirm equal contribution of these authors, able to take full responsibility for the paper and its content? While there is no limit per se to the number of co-corresponding authors, 4 co-corresponding authors is rare, and may not reflect as intended to the community.
- Please provide an institutional e-mail addresses for the co-corresponding authors Yang Zhao and Yunfei Xie.
- E-mail correspondence to Xingyu Chen could not be delivered. Please update the e-mail address and make sure to enter correct e-mail addresses for all authors in our submission system.
3) Affiliations: There are two affiliations associated with the number 3, please correct.
4) In the main manuscript file, please do the following:
- Please address all comments suggested by our data editors listed below:
o Data availability statement:
1. Please note that the specific URLs for PRJNA1304132 datasets are not provided in the data availability statement.
o Figure legends:
1. Please note that the exact p values are not provided in the legends of figures 1C, D, E, F, I, J, K, N; 3A, G; 4A, B, D, E; EV3 A, C.
2. Please indicate the statistical test used for data analysis in the legends of figures 2C, 3C, F; EV3 A-C; EV4 A.
3. Please note that information related to n is missing in the legends of figures 2E, F; 3C, EV3 A-C.
4. Please note that the error bars are not defined in the legends of figures EV3 A-C; EV4 A.
- Please change the headings and order of the sections in the manuscript text: Abstract / The Paper Explained / Introduction / Results / Discussion / Methods / Data Availability / Acknowledgements / Disclosure and Competing Interests Statement / References / Figure Legends / Tables / Expanded View Figure Legends
- In Methods, provide the antibody dilutions that were used for each antibody.
- In Methods, statistical paragraph should reflect all information that you have filled in the Authors Checklist, especially regarding randomization, blinding, replication.
- Indicate in legends number and nature of replicates and exact p= values, not a range, along with the statistical test used. To keep the figures "clear" some authors found providing an Appendix table Sx with all exact p-values preferable. You are welcome to do this if you want to.
- Please include structured Methods section that includes a Reagents and Tools Table (should be uploaded as a separate file) followed by a Methods and Protocols section. More information on how to adhere to this format as well as downloadable templates (.docx) for the Reagents and Tools Table can be found in our author guidelines:
https://www.embopress.org/page/journal/17574684/authorguide#structuredmethods
An example of a paper with Structured Methods can be found here:
https://www.embopress.org/doi/full/10.1038/s44320-024-00037-6#sec-4
- Add "Disclosure and competing interests statement". We updated our journal's competing interests policy in January 2022 and request authors to consider both actual and perceived competing interests. Please review the policy
https://www.embopress.org/competing-interests and update your competing interests if necessary.
- Author contributions: Please remove it from the manuscript and specify author contributions in our submission system. CRediT has replaced the traditional author contributions section because it offers a systematic machine-readable author contributions format that allows for more effective research assessment. You are encouraged to use the free text boxes beneath each contributing author's name to add specific details on the author's contribution. More information is available in our guide to authors:
https://www.embopress.org/page/journal/17574684/authorguide#authorshipguidelines
- In Data availability statement please use the following format to report the accession number of your data:

The datasets produced in this study are available in the following databases:
[data type]: [full name of the resource] [accession number/identifier] ([doi or URL or identifiers.org/DATABASE:ACCESSION])

Please check "Author Guidelines" for more information.
https://www.embopress.org/page/journal/17574684/authorguide#availabilityofpublishedmaterial
5) Funding: Please merge it with Acknowledgements and enter the funding information in our system to the line-by-line list of funders.
6) Tables: Please remove the legend from the manuscript text and add it to Table EV1.
7) The Paper Explained: Please provide "The Paper Explained" and add it to the main manuscript text. Please check "Author Guidelines" for more information. https://www.embopress.org/page/journal/17574684/authorguide#researcharticleguide

8) Synopsis: Every published paper now includes a 'Synopsis' to further enhance discoverability. Synopses are displayed on the journal webpage and are freely accessible to all readers. They include separate synopsis image and synopsis text.
- Synopsis image: Please provide a visual abstract as a high-resolution jpeg file 550 px-wide x 300-600 pixels high to illustrate your article.
- Synopsis text: Please provide a short standfirst (maximum of 300 characters, including space) as well as 2-5 one sentence bullet points that summarise the paper as a .doc file. Please write the bullet points to summarise the key NEW findings. They should be designed to be complementary to the abstract - i.e. not repeat the same text. We encourage inclusion of key acronyms and quantitative information (maximum of 30 words / bullet point). Please use the passive voice.
- Please check your synopsis text and image before submission with your revised manuscript. Please be aware that in the proof stage minor corrections only are allowed (e.g., typos).
9) As part of the EMBO Publications transparent editorial process (see our Editorial at http://embomolmed.embopress.org/content/2/9/329), EMBO Molecular Medicine will publish online a Review Process File (RPF) to accompany accepted manuscripts. This file will be published in conjunction with your paper and will include the anonymous referee reports, your point-by-point response and all pertinent correspondence relating to the manuscript. Let us know if you want to remove or not any figures from it prior to publication. Please note that the Authors checklist will be published at the end of the RPF.
10) Please provide a point-by-point letter INCLUDING my comments as well as the reviewer's reports and your detailed responses (as Word file).

I look forward to reading a new revised version of your manuscript as soon as possible.

Yours sincerely,

Zeljko Durdevic

Zeljko Durdevic
Senior Editor
EMBO Molecular Medicine

*** Instructions to submit your revised manuscript ***

*** PLEASE NOTE *** As part of the EMBO Publications transparent editorial process initiative (see our Editorial at https://www.embopress.org/doi/pdf/10.1002/emmm.201000094), EMBO Molecular Medicine will publish online a Review Process File to accompany accepted manuscripts.

In the event of acceptance, this file will be published in conjunction with your paper and will include the anonymous referee reports, your point-by-point response and all pertinent correspondence relating to the manuscript. If you do NOT want this file to be published, please inform the editorial office at contact@embomolmed.org.

*****

When preparing your revised manuscript, please refer to our guidelines: https://link.springer.com/journal/44321/submission-guidelines#cms-Revised-submissions. We perform an initial quality control of all revised manuscripts before re-review; failure to include requested items will delay the evaluation of your revision.

We require:

1) A .docx formatted version of the manuscript text (including legends for main figures, EV figures and tables). Please make sure that the changes are highlighted to be clearly visible.

2) Individual production quality figure files as .eps, .tif, .jpg (one file per figure). For guidance, download the 'Figure Guide PDF': https://media.springernature.com/original/springer-cms/rest/v1/content/27825798/data/v1.

3) A .docx formatted letter INCLUDING the reviewers' reports and your detailed point-by-point responses to their comments. As

part of the EMBO Press transparent editorial process, the point-by-point response is part of the Review Process File (RPF), which will be published alongside your paper.

4) A complete author checklist, which you can download from our author guidelines. Please insert information in the checklist that is also reflected in the manuscript. The completed author checklist will also be part of the RPF.

5) Please note that all corresponding authors are required to supply an ORCID ID for their name upon submission of a revised manuscript.

6) It is mandatory to include a 'Data Availability' section after the Materials and Methods. Before submitting your revision, primary datasets produced in this study need to be deposited in an appropriate public database, and the accession numbers and database listed under 'Data Availability'. Please remember to provide a reviewer password if the datasets are not yet public.

In case you have no data that requires deposition in a public database, please state so in this section. Note that the Data Availability Section is restricted to new primary data that are part of this study.

7) For data quantification: please specify the name of the statistical test used to generate error bars and P values, the number (n) of independent experiments (specify technical or biological replicates) underlying each data point and the test used to calculate p-values in each figure legend. The figure legends should contain a basic description of n, P and the test applied. Graphs must include a description of the bars and the error bars (s.d., s.e.m.).

8) At EMBO Press we ask authors to provide source data for the main manuscript figures. You will receive a separate email with instructions for providing source data with your revised manuscript, including how to upload and organize the files.

9) Our journal encourages inclusion of *data citations in the reference list* to directly cite datasets that were re-used and obtained from public databases. Data citations in the article text are distinct from normal bibliographical citations and should directly link to the database records from which the data can be accessed. In the main text, data citations are formatted as follows: "Data ref: Smith et al, 2001" or "Data ref: NCBI Sequence Read Archive PRJNA342805, 2017". In the Reference list, data citations must be labeled with "[DATASET]". A data reference must provide the database name, accession number/identifiers and a resolvable link to the landing page from which the data can be accessed at the end of the reference.

10) We replaced Supplementary Information with Expanded View (EV) Figures and Tables that are collapsible/expandable online. A maximum of 5 EV Figures can be typeset. EV Figures should be cited as 'Figure EV1, Figure EV2" etc... in the text and their respective legends should be included in the main text after the legends of regular figures.

- For the figures that you do NOT wish to display as Expanded View figures, they should be bundled together with their legends in a single PDF file called *Appendix*, which should start with a short Table of Content. Appendix figures should be referred to in the main text as: "Appendix Figure S1, Appendix Figure S2" etc.

- Additional Tables/Datasets should be labeled and referred to as Table EV1, Dataset EV1, etc. Legends have to be provided in a separate tab in case of .xls files. Alternatively, the legend can be supplied as a separate text file (README) and zipped together with the Table/Dataset file.

11) The paper explained: EMBO Molecular Medicine articles are accompanied by a summary of the articles to emphasize the major findings in the paper and their medical implications for the non-specialist reader. Please provide a draft summary of your article highlighting

- the medical issue you are addressing,

- the results obtained and

- their clinical impact.

This may be edited to ensure that readers understand the significance and context of the research. Please refer to any of our published articles for an example.

12) Author contributions: You will be asked to provide CRediT (Contributor Role Taxonomy) terms in the submission system. These replace a narrative author contribution section in the manuscript.

13) A Conflict of Interest statement should be provided in the main text.

14) Every published paper includes a 'Synopsis' to further enhance discoverability. Synopses are displayed on the journal webpage and are freely accessible to all readers. They include a short stand first (maximum of 300 characters, including space)

as well as 2-5 one-sentences bullet points that summarizes the paper. Please write the bullet points to summarize the key NEW findings. They should be designed to be complementary to the abstract - i.e. not repeat the same text. We encourage inclusion of key acronyms and quantitative information (maximum of 30 words / bullet point). Please use the passive voice. Please attach these in a separate file or send them by email, we will incorporate them accordingly.

Please also suggest a striking image or visual abstract to illustrate your article as a PNG file 550 px wide x 300-600 px high.

15) Include a Reagents and Tools Table as part of the Methods section, which can be downloaded from our author guidelines.

EMBO Molecular Medicine has a "scooping protection" policy, whereby similar findings that are published by others during review or revision are not a criterion for rejection. Should you decide to submit a revised version, I do ask that you get in touch after three months if you have not completed it, to update us on the status.

*Additional important information regarding Figures

Each figure should be given in a separate file and should have the following resolution:
Graphs 800-1,200 DPI
Photos 400-800 DPI
Colour (only CMYK) 300-400 DPI"

Figures are not edited by the production team. All lettering should be the same size and style; figure panels should be indicated by capital letters (A, B, C etc). Gridlines are not allowed except for log plots. Figures should be numbered in the order of their appearance in the text with Arabic numerals. Each Figure must have a separate legend and a caption is needed for each panel.

*Additional important information regarding figures and illustrations can be found at
https://media.springernature.com/original/springer-cms/rest/v1/content/27825798/data/v1

***** Reviewer's comments *****

Referee #1 (Comments on Novelty/Model System for Author):

The manuscript addresses an important topic with high novelty and potential medical impact. The overall study design is generally appropriate, and the authors have provided a comprehensive set of experiments to support their main conclusions. The model system used is adequate for the research question, and the findings are of potential translational relevance.

The authors have also carefully revised the manuscript in response to previous comments, and most concerns have been satisfactorily addressed. The data are generally well presented, and the conclusions are largely supported by the results.

One minor methodological point relates to the single-cell RNA-seq analysis. The study primarily relies on t-SNE for clustering, which is relatively outdated compared to current standard practices. Although the authors have supplemented the analysis with UMAP visualization, a more rigorous approach would be to consistently base downstream analyses (e.g., clustering-dependent results) on the updated UMAP-based framework. This would further enhance the methodological consistency and robustness of the single-cell component. Nevertheless, this issue does not materially affect the main conclusions of the study.

Overall, despite the above limitation, the study is well-conducted and provides valuable insights. I believe the manuscript is suitable for publication.

Referee #2 (Comments on Novelty/Model System for Author):

The methodology seems sound, and the experiments have been conducted appropriately from a technical standpoint. While the immediate clinical impact appears limited, the findings nonetheless open potential avenues for the application of betrixaban.

Referee #2 (Remarks for Author):

The presented findings are novel and scientifically intereresting. The authors have revised their manuscript and provided additional in vitro and in vivo data supporting their findings. They have employed additional tumor models as well as knockout strains to validate their claims. The authors have responded convincingly to all questions raised by the three referees.

Referee #3 (Comments on Novelty/Model System for Author):

The authors have addressed my questions. I recommend acceptance of this manuscript.

Referee #3 (Remarks for Author):

The authors have addressed my questions. I recommend acceptance of this manuscript.
Please ensure that gene symbols are italicized (e.g., cGAS, Isg15 and Rsad2).

Response to the Editor

We would like to thank the Editor for the constructive and detailed comments. Below we provide our point-by-point responses following the exact order of the editorial letter, with the reviewers' original comments included alongside our detailed replies.

1. Referee #1 (Comments on Novelty/Model System for Author):

The manuscript addresses an important topic with high novelty and potential medical impact. The overall study design is generally appropriate, and the authors have provided a comprehensive set of experiments to support their main conclusions. The model system used is adequate for the research question, and the findings are of potential translational relevance.

The authors have also carefully revised the manuscript in response to previous comments, and most concerns have been satisfactorily addressed. The data are generally well presented, and the conclusions are largely supported by the results.

One minor methodological point relates to the single-cell RNA-seq analysis. The study primarily relies on t-SNE for clustering, which is relatively outdated compared to current standard practices. Although the authors have supplemented the analysis with UMAP visualization, a more rigorous approach would be to consistently base downstream analyses (e.g., clustering-dependent results) on the updated UMAP-based framework. This would further enhance the methodological consistency and robustness of the single-cell component. Nevertheless, this issue does not materially affect the main conclusions of the study.

Overall, despite the above limitation, the study is well-conducted and provides valuable insights. I believe the manuscript is suitable for publication.

Response: We sincerely thank the reviewer for this helpful suggestion. We agree that UMAP is now widely used in single-cell RNA-seq analysis and may provide better methodological consistency when applied throughout the entire downstream workflow.

In this study, however, t-SNE was mainly used for visualization, and the major downstream biological conclusions were supported by multiple complementary analyses rather than relying solely on the clustering display method itself. We have also supplemented the manuscript with UMAP visualization, which yielded consistent overall patterns and did not alter the interpretation of the results. Therefore, given that this issue does not materially affect the main conclusions, we respectfully did not further re-run all clustering-dependent downstream analyses under a fully UMAP-based framework in the current revision.

We are grateful for this constructive comment and will carefully consider adopting a more unified UMAP-based workflow in future work.We hope the reviewer finds this clarification reasonable.

2. Referee #3 (Comments on Novelty/Model System for Author):

The authors have addressed my questions. I recommend acceptance of this manuscript.

Referee #3 (Remarks for Author):

The authors have addressed my questions. I recommend acceptance of this manuscript.

Please ensure that gene symbols are italicized (e.g., cGAS, Isg15 and Rsad2).

Response: As suggested, we have italicized all gene symbols (including cGAS, Isg15, and Rsad2) throughout the manuscript to comply with the standard formatting.

7th Apr 2026

Dear Dr. Zhao,

We are pleased to inform you that your manuscript is accepted for publication and is now being sent to our publisher to be included in the next available issue of EMBO Molecular Medicine.

Your manuscript will be processed for publication by EMBO Press. It will be copy edited and you will receive page proofs prior to publication. Please note that you will be contacted by Springer Nature Author Services to complete licensing and payment information.

You may qualify for financial assistance for your publication charges - either via a Springer Nature fully open access agreement or an EMBO initiative. Check your eligibility: https://link.springer.com/journal/44321/how-to-publish-with-us

Should you be planning a Press Release on your article, please get in contact with embo_production@springernature.com as early as possible in order to coordinate publication and release dates.

If you have any questions, please do not hesitate to contact the Editorial Office. Thank you for your contribution to EMBO Molecular Medicine.

Yours sincerely,
Zeljko Durdevic

Zeljko Durdevic
Senior Editor
EMBO Molecular Medicine

>>> Please note that it is EMBO Molecular Medicine policy for the transcript of the editorial process (containing referee reports and your response letter) to be published as an online supplement to each paper. If you do NOT want this, you will need to inform the Editorial Office via email immediately. More information is available here: https://link.springer.com/partners/embo-press/editorial-policies#Peer%20review

EMBO Press Author Checklist

Corresponding Author Name: Yang Zhao, Yunfei Xie, Songying Ouyang, Fuping You	
Journal Submitted to: EMBO Molecular Medicine	
Manuscript Number: EMM-2025-22966	

USEFUL LINKS FOR COMPLETING THIS FORM
The EMBO Journal - Author Guidelines
EMBO Reports - Author Guidelines
Molecular Systems Biology - Author Guidelines
EMBO Molecular Medicine - Author Guidelines

Reporting Checklist for Life Science Articles (updated January
This checklist is adapted from Materials Design Analysis Reporting (MDAR) Checklist for Authors. MDAR establishes a minimum set of requirements in transparent reporting in the life sciences (see Statement of Task: 10.31222/osf.io/9sm4x). Please follow the journal's guidelines in preparing your
Please note that a copy of this checklist will be published alongside your article.

Abridged guidelines for figures
1. Data
The data shown in figures should satisfy the following conditions:
- the data were obtained and processed according to the field's best practice and are presented to reflect the results of the experiments in an accurate and unbiased manner.
- ideally, figure panels should include only measurements that are directly comparable to each other and obtained with the same assay.
- plots include clearly labeled error bars for independent experiments and sample sizes. Unless justified, error bars should not be shown for technical
- if n<5, the individual data points from each experiment should be plotted. Any statistical test employed should be justified.
- Source Data should be included to report the data underlying figures according to the guidelines set out in the authorship guidelines on Data

2. Captions
Each figure caption should contain the following information, for each panel where they are relevant:
- a specification of the experimental system investigated (eg cell line, species name).
- the assay(s) and method(s) used to carry out the reported observations and measurements.
- an explicit mention of the biological and chemical entity(ies) that are being measured.
- an explicit mention of the biological and chemical entity(ies) that are altered/varied/perturbed in a controlled manner.
- the exact sample size (n) for each experimental group/condition, given as a number, not a range;
- a description of the sample collection allowing the reader to understand whether the samples represent technical or biological replicates (including how many animals, litters, cultures, etc.).
- a statement of how many times the experiment shown was independently replicated in the laboratory.
- definitions of statistical methods and measures:
  - common tests, such as t-test (please specify whether paired vs. unpaired), simple χ2 tests, Wilcoxon and Mann-Whitney tests, can be unambiguously identified by name only, but more complex techniques should be described in the methods section;
  - are tests one-sided or two-sided?
  - are there adjustments for multiple comparisons?
  - exact statistical test results, e.g., P values = x but not P values < x;
  - definition of 'center values' as median or average;
  - definition of error bars as s.d. or s.e.m.

Please complete ALL of the questions below.
Select "Not Applicable" only when the requested information is not relevant for your study.

Materials

Newly Created Materials	Information included in the manuscript?	In which section is the information available? (Reagents and Tools Table, Materials and Methods, Figures, Data Availability Section)
New materials and reagents need to be available; do any restrictions apply?	Yes	Materials and methods

Antibodies	Information included in the manuscript?	In which section is the information available? (Reagents and Tools Table, Materials and Methods, Figures, Data Availability Section)
For **antibodies** provide the following information:<br>- Commercial antibodies: RRID (if possible) or supplier name, catalogue number and or/clone number<br>- Non-commercial: RRID or citation	Yes	Materials and methods

DNA and RNA sequences	Information included in the manuscript?	In which section is the information available? (Reagents and Tools Table, Materials and Methods, Figures, Data Availability Section)
**Short novel DNA or RNA including primers, probes**: provide the sequences.	Yes	Supplementary Table 1. Primer sequences used for RT-qPCR

Cell materials	Information included in the manuscript?	In which section is the information available? (Reagents and Tools Table, Materials and Methods, Figures, Data Availability Section)
**Cell lines:** Provide species information, strain. Provide accession number in repository **OR** supplier name, catalog number, clone number, and/**OR** RRID.	Yes	Materials and methods
**Primary cultures:** Provide species, strain, sex of origin, genetic modification status.	Yes	Materials and methods
Report if the cell lines were recently **authenticated** (e.g., by STR profiling) and tested for mycoplasma contamination.	Yes	Materials and methods

Experimental animals	Information included in the manuscript?	In which section is the information available? (Reagents and Tools Table, Materials and Methods, Figures, Data Availability Section)
**Laboratory animals or Model organisms:** Provide species, strain, sex, age, genetic modification status. Provide accession number in repository **OR** supplier name, catalog number, clone number, **OR** RRID.	Yes	Materials and methods
**Animal observed in or captured from the field:** Provide species, sex, and age where possible.	Not Applicable	
Please detail **housing and husbandry conditions**.	Yes	Materials and methods

Plants and microbes	Information included in the manuscript?	In which section is the information available? (Reagents and Tools Table, Materials and Methods, Figures, Data Availability Section)
**Plants:** provide species and strain, ecotype and cultivar where relevant, unique accession number if available, and source (including location for collected wild specimens).	Not Applicable	
**Microbes:** provide species and strain, unique accession number if available, and source.	Yes	Materials and methods

Human research participants	Information included in the manuscript?	In which section is the information available? (Reagents and Tools Table, Materials and Methods, Figures, Data Availability Section)
If collected and within the bounds of privacy constraints report on age, sex and gender or ethnicity for all study participants.	Not Applicable	

Core facilities	Information included in the manuscript?	In which section is the information available? (Reagents and Tools Table, Materials and Methods, Figures, Data Availability Section)
If your work benefited from core facilities, was their service mentioned in the acknowledgments section?	Yes	Acknowledgments

Design

Study protocol	Information included in the manuscript?	In which section is the information available? (Reagents and Tools Table, Materials and Methods, Figures, Data Availability Section)
If study protocol has been **pre-registered, provide DOI in the manuscript**. For clinical trials, provide the trial registration number **OR** cite DOI.	Not Applicable	
Report the **clinical trial registration number** (at ClinicalTrials.gov or equivalent), where applicable.	Not Applicable	

Laboratory protocol	Information included in the manuscript?	In which section is the information available? (Reagents and Tools Table, Materials and Methods, Figures, Data Availability Section)
Provide DOI OR other citation details if **external detailed step-by-step protocols** are available.	Not Applicable	

Experimental study design and statistics	Information included in the manuscript?	In which section is the information available? (Reagents and Tools Table, Materials and Methods, Figures, Data Availability Section)
Include a statement about **sample size** estimate even if no statistical methods were used.	Yes	Materials and methods
Were any steps taken to minimize the effects of subjective bias when allocating animals/samples to treatment (e.g. **randomization procedure**)? If yes, have they been described?	Yes	Materials and methods
Include a statement about **blinding** even if no blinding was done.	Not Applicable	
Describe **inclusion/exclusion criteria** if samples or animals were excluded from the analysis. Were the criteria pre-established? If sample or data points were omitted from analysis, report if this was due to attrition or intentional exclusion and provide justification.	Not Applicable	
For every figure, are **statistical tests** justified as appropriate? Do the data meet the assumptions of the tests (e.g., normal distribution)? Describe any methods used to assess it. Is there an estimate of variation within each group of data? Is the variance similar between the groups that are being statistically compared?	Yes	Materials and methods

Sample definition and in-laboratory replication	Information included in the manuscript?	In which section is the information available? (Reagents and Tools Table, Materials and Methods, Figures, Data Availability Section)
In the figure legends: state number of times the experiment was **replicated** in laboratory.	Yes	Figures
In the figure legends: define whether data describe **technical or biological replicates**.	Yes	Figures

Ethics

Ethics	Information included in the manuscript?	In which section is the information available? (Reagents and Tools Table, Materials and Methods, Figures, Data Availability Section)
Studies involving **human participants**: State details of **authority granting ethics approval** (IRB or equivalent committee(s), provide reference number for approval.	Not Applicable	
Studies involving **human participants**: Include a statement confirming that **informed consent** was obtained from all subjects and that the experiments conformed to the principles set out in the WMA Declaration of Helsinki and the Department of Health and Human Services Belmont Report.	Not Applicable	
Studies involving **human participants: For publication of patient photos**, include a statement confirming that consent to publish was obtained.	Not Applicable	
Studies involving experimental **animals**: State details of **authority granting ethics approval** (IRB or equivalent committee(s), provide reference number for approval. Include a statement of compliance with ethical regulations.	Yes	Materials and methods
Studies involving **specimen and field samples:** State if relevant **permits** obtained, provide details of authority approving study; if none were required, explain why.	Not Applicable	

Dual Use Research of Concern (DURC)	Information included in the manuscript?	In which section is the information available? (Reagents and Tools Table, Materials and Methods, Figures, Data Availability Section)
Could your study fall under dual use research restrictions? Please check biosecurity documents and list of **select agents and toxins** (CDC): https://www.selectagents.gov/sat/list.htm	Not Applicable	
If you used a select agent, is the security level of the lab appropriate and reported in the manuscript?	Not Applicable	
If a study is subject to dual use research of concern regulations, is the name of the **authority granting approval and reference number** for the regulatory approval provided in the manuscript?	Not Applicable	

Reporting

The MDAR framework recommends adoption of discipline-specific guidelines, established and endorsed through community initiatives. Journals have their own policy about requiring specific guidelines and recommendations to complement MDAR.

Adherence to community standards	Information included in the manuscript?	In which section is the information available? (Reagents and Tools Table, Materials and Methods, Figures, Data Availability Section)
State if relevant guidelines or checklists (e.g., **ICMJE, MIBBI, ARRIVE, PRISMA**) have been followed or provided.	Not Applicable	
For **tumor marker prognostic studies,** we recommend that you follow the **REMARK** reporting guidelines (see link list at top right). See author guidelines, under 'Reporting Guidelines'. Please confirm you have followed these guidelines.	Not Applicable	
For **phase II and III randomized controlled trials**, please refer to the **CONSORT** flow diagram (see link list at top right) and submit the CONSORT checklist (see link list at top right) with your submission. See author guidelines, under 'Reporting Guidelines'. Please confirm you have submitted this list.	Not Applicable	

Data Availability

Data availability	Information included in the manuscript?	In which section is the information available? (Reagents and Tools Table, Materials and Methods, Figures, Data Availability Section)
Have **primary datasets** been deposited according to the journal's guidelines (see 'Data Deposition' section) and the respective accession numbers provided in the Data Availability Section?	Yes	Data Availability Section
Were **human clinical and genomic datasets** deposited in a public access-controlled repository in accordance to ethical obligations to the patients and to the applicable consent agreement?	Not Applicable	
Are **computational models** that are central and integral to a study available without restrictions in a machine-readable form? Were the relevant accession numbers or links provided?	Not Applicable	
If publicly available data were reused, provide the respective **data citations in the reference list**.	Not Applicable	
